# Bifunctional glycosphingolipid (GSL) probes to investigate GSL-interacting proteins in cell membranes

**DOI:** 10.1016/j.jlr.2024.100570

**Published:** 2024-05-23

**Authors:** Sayan Kundu, Rajendra Rohokale, Chuwei Lin, Sixue Chen, Shayak Biswas, Zhongwu Guo

**Affiliations:** 1 Department of Chemistry, University of Florida, Gainesville, FL, USA; 2 Department of Biology, Genetics Institute, University of Florida, Gainesville, FL, USA; 3 Department of Biology, University of Mississippi, Oxford, MS, USA

**Keywords:** glycolipids, glycosphingolipids, ceramides, chemical synthesis, proteomics, fluorescence microscopy, protein-lipid interaction, diazirine, photoactivated crosslinking, click reaction

## Abstract

Glycosphingolipids (GSLs) are abundant glycolipids on cells and essential for cell recognition, adhesion, signal transduction, and so on. However, their lipid anchors are not long enough to cross the membrane bilayer. To transduce transmembrane signals, GSLs must interact with other membrane components, whereas such interactions are difficult to investigate. To overcome this difficulty, bifunctional derivatives of II^3^-β-*N*-acetyl-D-galactosamine-GA2 (GalNAc-GA2) and β-*N*-acetyl-D-glucosamine-ceramide (GlcNAc-Cer) were synthesized as probes to explore GSL-interacting membrane proteins in live cells. Both probes contain photoreactive diazirine in the lipid moiety, which can crosslink with proximal membrane proteins upon photoactivation, and clickable alkyne in the glycan to facilitate affinity tag addition for crosslinked protein pull-down and characterization. The synthesis is highlighted by the efficient assembly of simple glycolipid precursors followed by on-site lipid remodeling. These probes were employed to profile GSL-interacting membrane proteins in HEK293 cells. The GalNAc-GA2 probe revealed 312 distinct proteins, with GlcNAc-Cer probe-crosslinked proteins as controls, suggesting the potential influence of the glycan on GSL functions. Many of the proteins identified with the GalNAc-GA2 probe are associated with GSLs, and some have been validated as being specific to this probe. The versatile probe design and experimental protocols are anticipated to be widely applicable to GSL research.

## Graphical abstract

**Figure:**
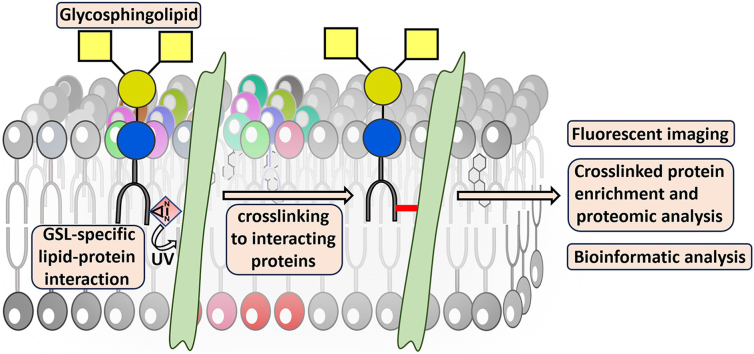

Glycosphingolipids (GSLs) are glycolipids consisting of a hydrophilic glycan as the head group and a hydrophobic ceramide (Cer) as the backbone, which are stitched together by a glycosidic bond (Fig. 1) (1). Their distinctive structures and biophysical properties, for example, being able to form specific microdomains in the cell membrane (2, 3, 4, 5), warrant GSLs a unique niche in cell biology. For example, GSLs are a principal and essential membrane constituent (6, 7, 8) functioning as receptors on the cell surface (9, 10); thus, many exoplasmic and cell surface proteins contain GSL-binding domains (11, 12, 13). As such, GSLs are involved in regulating various physiological and pathological events like signal transduction (14), cell differentiation and proliferation (15, 16, 17), cancer (18), Alzheimer's disease (19, 20, 21), and microbial infection (22, 23, 24). LcGg4 in Fig. 1 is an illustrative example of GSLs, representing the LcGg series core structure (25), and a leukemia-associated GSL antigen as well (26). It has been further demonstrated that the interactions between GSLs and their ligands are regulated by both their glycan moiety and their lipid anchor (9, 27).

**Fig. 1:**
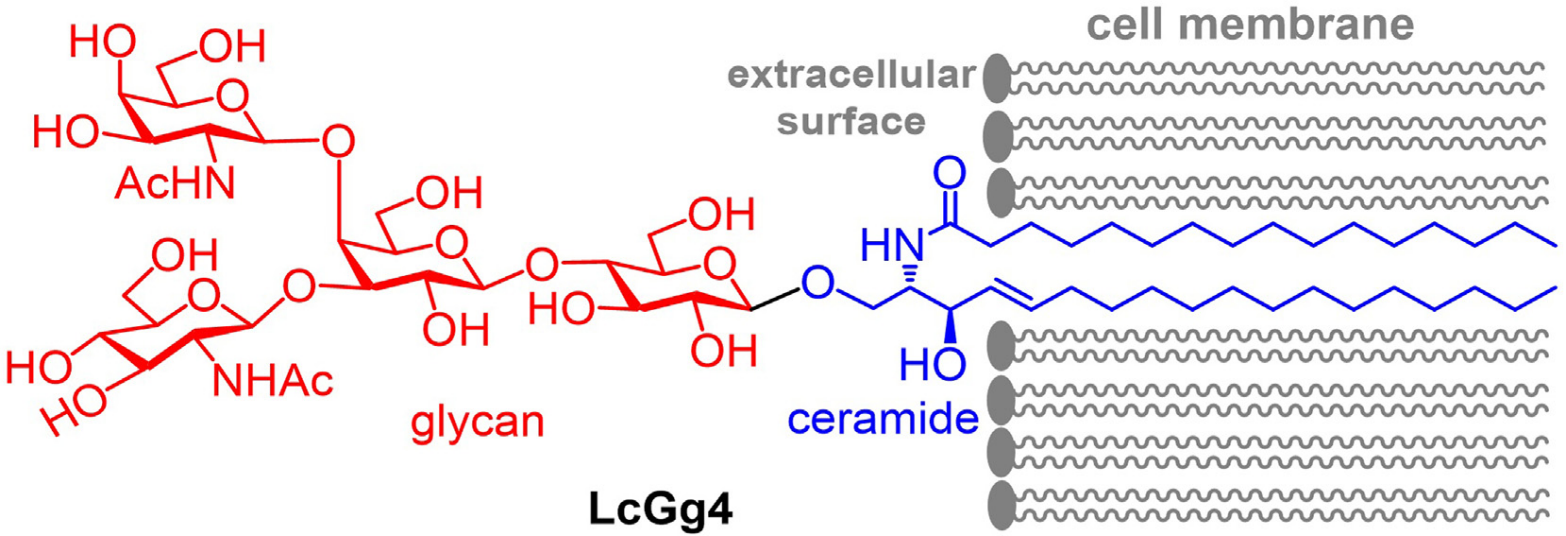
The chemical structure of LcGg4, a representative GSL and a cancer antigen, and its association with the cell membrane.

Despite the documented involvement of GSLs in various cellular activities, the exact mechanisms by which GSLs transduce binding signals through the cell membrane remain ambiguous. In general, it is believed that GSLs can generate specific microdomains in the membrane, such as the “lipid rafts,” which serve as platforms to recruit other biomolecules to facilitate their interactions with GSLs (28, 29, 30, 31). Although extracellular ligands can directly bind GSLs (31, 32), the lipid chains of GSLs are usually not long enough to cross the entire membrane bilayer. To transduce signals induced by GSL binding with extracellular ligands across the cell membrane, GSLs must communicate with other cell membrane components. Unfortunately, cell membrane components interacting with GSLs are difficult to identify due to the highly diverse, complex, and dynamic structures and nature of GSLs and the cell membrane.

To investigate lipid–protein interactions, several proteome-wide mapping methods have been explored, which include microarrays of various metabolites to profile lipid-binding proteins (33), proteome chip-based screening (34), affinity-based protein purification by columns or lipid immobilized magnetic beads (35, 36), and so on. Various model membranes (e.g., nanodiscs, liposomes, and supported bilayers) have also been employed to examine the interactions of GSLs with proteins by mass spectrometry (MS) and competitive ligand binding assays (37, 38). As a result, many proteins, such as Galectin (39), death receptor Fas (40), notch ligand delta-like 1 protein (41), Serotonin (42), insulin receptors (43), and numerous growth factor receptors (44, 45, 46, 47, 48, 49, 50, 51, 52), have been found to interact with GSLs. However, a main limitation of these methods is that the lipids or proteins immobilized onto solid supports are not in their natural state. Moreover, the most important mechanism of lipid-protein interactions, that is, hydrophobic matching between the lipid tails of GSLs and the hydrophobic domains of proteins is likely to be neglected. To address these problems, specially functionalized probes have drawn significant attention in recent years. Typically, such probes contain two functional groups, one crosslinking with target molecules and the other enabling the isolation and characterization of cross-linked molecules (53, 54, 55, 56, 57, 58). A trifunctional probe containing photocage, photoreactive diazirine, and clickable alkyne moieties was also developed to explore the signaling events of sphingosine and diacylglycerol (59).

The present research aimed to develop a practicable approach for the identification of membrane proteins interacting with GSLs. To this end, we have designed and synthesized two bifunctional GSL analogs that contain photoreactive diazirine to facilitate GSL crosslinking with interacting proteins and clickable alkyne to enable the introduction of an affinity tag for crosslinked protein isolation. These GSL analogs were used as demonstrating probes to explore GSL-interacting proteins in the membrane of HEK293 cells.

## Materials and methods

### General methods and materials

Chemicals and materials were purchased from commercial sources and used as received without further purification unless noted otherwise. Molecular sieves 4Å (MS 4Å) were flame-dried under a high vacuum and used immediately after being cooled to rt in an N_2_ atmosphere. Analytical thin layer chromatography (TLC) was carried out on silica gel 60 Å F254 plates with detection by a UV detector and/or by charring with 10% (v/v) H_2_SO_4_ in ethanol. Flash column chromatography was performed on silica gel 60 (230–400 Mesh). NMR spectra were acquired on a 400 or 600 MHz NMR spectrometer with chemical shifts reported in ppm (δ) referenced to CDCl_3_ (residual ^1^H NMR: δ 7.26 ppm, ^13^C NMR: δ 77.16 ppm) or CD_3_OD (residual ^1^H NMR: δ 3.31 ppm, ^13^C NMR: δ 49.0 ppm). Peak and coupling constant assignments are made based on ^1^H, ^1^H–^1^H COSY, ^1^H-^13^C HSQC, and ^1^H-^13^C HMBC experiments, and structural elucidations were made with additional information from gCOSY, gHSQC, and gHMBC experiments. Aluminum heating blocks were used for heating. Paraformaldehyde (PFA), copper sulfate (CuSO_4_), sodium ascorbate, poly-L-lysine, and tris(2-carboxyethyl) phosphine hydrochloride (TCEP) were from Sigma Aldrich. Fetal bovine serum (FBS), Dulbecco’s Modified Eagle’s Medium (DMEM), and penicillin-streptomycin solution were from the American Type Culture Collection (ATCC). Dulbecco’s phosphate buffer saline (DPBS), 4′,6-diamidino2-phenylindole (DAPI), streptavidin Agarose resin, Cy5-azide, bovine serum albumin (BSA), Pierce™ anti-DYKDDDDK magnetic agarose beads, and lipofectamine™ 2000 transfection reagent were from Thermo Fisher scientific. Biotin-azide (or Biotin-PEG3-Azide) and tris-(3-hydroxypropyltriazolylmethyl)amine (THPTA) were from Click Chemistry Tools. Streptavidin-A488 was from Santa Cruz Biotech. RPMI 1640 buffer was from Lonza. Fluorescent imaging was performed on an Olympus IX71 inverted system equipped with LED light source (Cool LED, PE-300), 20X 0.8 and 60X 1.25 NA plan apochromatic objectives (Olympus LUCPlanFl N objective), DAPI and Cy5 fluorescence channels, and Olympus DP23M color camera. Image analysis was performed using Olympus Cellsens Standard 3 and FIJI/ImageJ software. The cell lysis buffer contained 4% sodium dodecyl sulfate (SDS), 120 mM NaCl, and 50 mM triethanolamine. The cell permeabilization buffer is 1× DPBS with 0.1% Triton-X-100% and 2.5% BSA. For VPS36, SNX5, and RAB27A protein overexpression, VPS36, SNX5, and RAB27A cDNA ORF Clones, Human, Flag tag plasmids were purchased from Sino Biological. Organelle markers utilized in this work are listed in the Supplemental data with corresponding catalog numbers.

### (2S,3R)-2-(tert-Butyloxycarbonyl)amino-3-pivaloyloxypent-4-en-1-yl 3,4,6-tri-O-acetyl-2-deoxy-2-(2,2,2-trichloroethoxycarbonylamino)-β-D-glucopyranoside (6)

A mixture of **4** (0.61 g, 0.98 mmol), **5** (0.27 g, 0.89 mmol), and flame-dried MS 4Å (1.0 g) in dry dichloromethane (DCM, 10 ml) was stirred at room temperature (rt) under Argon for 30 min before cooling to −78°C. Trimethylsilyl triflate (TMSOTf, 8.2 μl, 0.05 mmol) was added, and the mixture was allowed to warm to −30°C and kept at this temperature for 1 h. TLC analysis confirmed the complete consumption of starting materials, and trimethylamine was added to quench the reaction. The mixture was filtered through a Celite pad, and the filtrate was concentrated in a vacuum. The product was purified by flash chromatography (Hexane/EtOAc 1/1) to afford **6** as a colorless syrup (0.60 g, 88%). TLC: R_f_ = 0.46 (Hexane/EtOAc 1:1). ^1^H NMR (600 MHz, CDCl_3_): δ 5.77 (ddd, J = 17.1, 10.6, 6.1 Hz, 1H, =CH), 5.40–5.26 (m, 3H, -C=CH_2_), 5.24–5.22 (m, 2H), 5.04 (t, J = 9.7 Hz, 1H,H3), 4.85 (d, J = 7.7 Hz, 1H,), 4.81 (d, J = 12.0 Hz, 1H, O-CH-CCl_3_), 4.61 (d, J = 3.6 Hz, 1H), 4.59 (d, J = 7.8 Hz, 1H, anomeric H), 4.21 (dd, J = 12.3, 4.8 Hz, 1H), 4.16–4.07 (m, 1H), 4.06–3.99 (m, 1H), 3.95 (dd, J = 9.9, 4.2 Hz, 1H), 3.72–3.60 (m, 2H), 3.56 (dd, J = 9.6, 3.7 Hz, 1H), 2.06 (s, 3H), 2.01 (s, 3H), 2.01 (s, 3H), 1.41 (s, 9H), 1.18 (s, 9H). ^13^C NMR (151 MHz, CDCl_3_): δ 177.0, 170.9, 170.7, 169.5, 155.5, 154.2, 133.2, 118.7, 100.8, 100.7, 95.5, 79.8, 74.6, 73.3, 73.3, 71.9, 71.9, 68.6, 68.0, 62.1, 60.5, 56.2, 52.0, 38.9, 28.5, 27.1, 20.8, 20.7, 20.7. HRMS (ESI-TOF) m/z: [M + H]^+^ Calcd for C_30_H_46_Cl_3_N_2_O_14_ 763.2009; Found 763.2022.

### (2S,3R,E)-2-(tert-Butyloxycarbonyl)amino-3-pivaloyloxyoctadec-4-en-1-yl 3,4,6-tri-O-acetyl-2-deoxy-2-(2,2,2-trichloroethoxycarbonylamino)-β-D-glucopyranoside (8)

To a solution of **6** (201 mg, 0.26 mmol) and pentadec-1-ene **7** (0.43 ml, 1.58 mmol) in dry DCM (65 ml) was added second-generation Hoveyda−Grubbs catalyst (16.5 mg, 0.026 mmol). The mixture was refluxed for 5 days, while a batch of **7** (0.43 ml, 1.58 mmol) and Hoveyda−Grubbs catalyst (5 mol%) was added at 24-h intervals. After the reaction was complete, 2 drops of dimethyl sulfoxide (DMSO) were added at rt, followed by stirring for another 2 h. The mixture was concentrated, and the product was purified by silica gel column chromatography to give **8** (206 mg, 83%) as a white solid. TLC: R_f_ = 0.7 (Hexane/EtOAc 2:3). ^1^H NMR (600 MHz, CDCl_3_): δ 5.77 (dt, J = 14.1, 6.7 Hz, 1H, C=CH-CH_2_), 5.35 (dd, J = 15.3, 7.4 Hz, 1H, -C=CH-C), 5.28–5.18 (m, 3H), 5.06 (t, J = 9.6 Hz, 1H), 4.94–4.71 (m, 2H), 4.64–4.64 (m, 2H), 4.22 (dd, J = 12.3, 4.7 Hz, 1H), 4.10 (dd, J = 12.3, 2.2 Hz, 1H), 4.01–3.95 (m, 1H), 3.93 (dd, J = 9.9, 4.3 Hz, 1H), 3.72–3.65 (m, 1H), 3.62 (q, J = 8.8 Hz, 1H), 3.59–3.53 (m, 1H), 2.07 (s, 3H), 2.03 (s, 3H), 2.02 (s, 3H), 2.01–1.96 (m, 2H), 1.42 (s, 9H), 1.38–1.22 (m, 22H), 1.17 (s, 9H), 0.87 (t, J = 7.0 Hz, 3H). ^13^C NMR (151 MHz, CDCl_3_): δ 176.9, 170.7, 169.5, 155.5, 154.2, 137.1, 124.6, 100.6, 95.5, 79.8, 74.6, 73.3, 72.0, 71.9, 68.6, 68.1, 62.1, 56.3, 52.2, 38.9, 32.4, 32.0, 29.8, 29.7, 29.7, 29.6, 29.4, 29.3, 29.0, 28.5, 27.1, 22.8, 20.8, 20.7, 20.7. HRMS (ESI-TOF) m/z: [M + H]^+^ Calcd for C_43_H_72_Cl_3_N_2_O_14_ 945.4062; Found 945.4044.

### (2S,3R,E)-2-(tert-Butyloxycarbonyl)amino-3-pivaloyloxyoctadec-4-en-1-yl 3,4,6-tri-O-acetyl-2-deoxy-2-(pent-4-ynamido)-β-D-glucopyranoside (9)

To a solution of **8** (39 mg, 0.041 mmol) in acetic acid (AcOH)/tetrahydrofuran (1:3, 2 ml) was added activated Zn (134.5 mg, 2.06 mmol). After the mixture was stirred at rt for 12 h, it was filtered through a Celite pad, and the filtrate was concentrated in vacuo. The product was washed with dry toluene (3 × 3 ml) and then dissolved in 1 ml of dry DCM. To this solution were added pent-4-yonic acid (20.1 mg, 0.204 mmol), 1-ethyl-3-(3-dimethylaminopropyl)carbodiimide (EDC, 39.2 mg, 0.204 mmol), and 4-dimethylaminopyridine (DMAP, 5 mg, 0.041 mmol) in 2 ml of dry DCM at 0°C. The mixture was stirred at rt overnight and diluted with DCM. The organic layer was washed with saturated NaHCO_3_, water, and brine, and dried over Na_2_SO_4_. The solution was concentrated under vacuum and the residue was separated by silica gel column chromatography to give **9** (24.8 mg, 71%) as colorless syrup. TLC: R_f_ = 0.74 (EtOAc/Hexane 4:1). ^1^H NMR (600 MHz, CDCl_3_): δ 5.88–5.70 (m, 2H), 5.35 (dd, J = 15.3, 7.4 Hz, 1H), 5.31–5.25 (m, 1H), 5.20 (t, J = 7.1 Hz, 1H), 5.05 (t, J = 9.6 Hz, 1H), 4.87 (d, J = 9.5 Hz, 1H, -CONH), 4.65 (d, J = 8.2 Hz, 1H, anomeric), 4.22 (dd, J = 12.3, 4.8 Hz, 1H), 4.10 (dd, J = 12.2, 2.2 Hz, 1H), 4.01–3.93 (m, 1H), 3.91 (dd, J = 9.9, 4.5 Hz, 1H), 3.83 (q, J = 8.5 Hz, 1H), 3.74–3.64 (m, 1H), 3.54 (dd, J = 9.8, 3.4 Hz, 1H), 2.53–2.46 (m, 2H), 2.42–2.33 (m, 2H), 2.07 (s, 3H), 2.03 (s, 4H, 3H × CH_3_ and 1H acetylene), 2.01 (s, 3H), 2.01–1.95 (m, 2H), 1.42 (s, 9H), 1.36–1.31 (m, 2H), 1.31–1.20 (m, 20H), 1.17 (s, 9H), 0.87 (t, J = 7.0 Hz, 3H). ^13^C NMR (151 MHz, CDCl_3_): δ 176.9, 171.3, 170.9, 170.6, 169.4, 155.4, 136.9, 124.5, 100.6, 82.8, 82.8, 79.5, 73.3, 72.0, 71.9, 69.5, 68.5, 67.7, 62.1, 54.7, 52.2, 38.7, 35.4, 32.3, 31.9, 29.6, 29.6, 29.6, 29.4, 29.3, 29.19, 28.9, 28.4, 27.0, 22.6, 20.7, 20.7, 20.6, 14.7, 14.1. HRMS (ESI-TOF) m/z: [M + H]^+^ Calcd for C_45_H_75_Cl_3_N_2_O_12_ 851. 5264; Found 851. 5281.

### (2S,3R,E)-2-[11-(3-Hexyl-3H-diazirin-3-yl)undecanamido]-3-(pivaloyloxy)-octadec-4-en-1-yl 3,4,6-tri-O-acetyl-2-deoxy-2-(pent-4-ynamido)-β-D-glucopyranoside (11)

Trifluoracetic acid (TFA, 107 μl, 1.4 mmol) was added to a stirred solution of **9** (17 mg, 0.02 mmol) in DCM (3 ml) at rt, and the mixture was stirred until the disappearance of **9**. The solvent was removed in vacuo, and the residue was dissolved in dry DCM (3 ml), followed by adding EDC (7.54 mg, 0.039 mmol), DMAP (1.4 mg, 0.011 mmol), and **10** (12.2 mg, 0.039 mmol) at 0°C. The mixture was stirred under Argon at rt for 12 h. After the completion of the reaction, water was added. The organic layer was separated and washed with saturated NaHCO_3_, water, and brine and dried over Na_2_SO_4_. The solvent was removed in vacuo, and the residue was purified by silica gel column chromatography to afford **11** (18.4 mg, 90%) as colorless syrup. TLC: R_f_ = 0.8 (EtOAc/Hexane, 4:1). ^1^H NMR (600 MHz, CDCl_3_): δ 6.96 (d, J = 8.7 Hz, 1H,-NH), 6.02 (d, J = 9.1 Hz, 1H, -NH), 5.86–5.82 (m, 2H), 5.80 (t, J = 7.2 Hz, 1H), 5.39–5.27 (m, 3H), 5.27–5.17 (m, 2H), 5.10–5.01 (m, 2H), 4.68 (d, J = 8.3 Hz, 1H, anomeric), 4.37–4.37 (m, 1H), 4.26–4.21 (m, 2H), 4.11 (dd, J = 12.3, 2.3 Hz, 1H), 3.98–3.91 (m, 1H), 3.91–3.81 (m, 1H), 3.84–3.78 (m, 1H), 3.74–3.65 (m, 3H), 2.58–2.39 (m, 2H), 2.41–2.28 (m, 2H), 2.08 (s, 3H), 2.04 (s, 3H), 2.03 (s, 5H, 3H × CH_3_ and 2H -COCH_2_-) 2.02–1.99 (m, 2H), 1.97 (t, J = 2.5 Hz, 1H, acetylene), 1.65–1.52 (m, 2H), 1.37–1.20 (m, 49H), 1.19 (s, 9H), 1.11–1.01 (m, 1H), 0.88 (t, J = 7.0 Hz, 6H). ^13^C NMR (151 MHz, CDCl_3_): δ 177.1, 171.8, 171.2, 170.7, 169.4, 157.2, 138.1, 123.7, 100.3, 82.7, 72.7, 72.3, 72.2, 69.5, 68.4, 65.4, 62.1, 54.5, 51.6, 39.0, 35.4, 33.0, 32.4, 32.0, 29.8, 29.8, 29.7, 29.6, 29.5, 29.5, 29.5, 29.2, 28.9, 27.1, 22.8, 20.8, 20.8, 20.7, 14.7, 14.2. HRMS (ESI-TOF) m/z: [M + H]^+^ Calcd for C_58_H_99_N_2_O_12_ 1043.7259; Found 1043.7269.

### (2S,3R,E)-2-[11-(3-hexyl-3H-diazirin-3-yl)undecanamido]-octadec-4-en-1-yl 2-deoxy-2-(pent-4-ynamido)-β-D-glucopyranoside (2)

To a solution of **11** (18 mg, 0.017 mmol) in dry MeOH/DCM (3:2, 2 ml) was added NaOMe in MeOH (4.5 M, 38.3 μl, 0.172 mmol) at 0°C. After the solution was stirred at rt for 2 days, the mixture was neutralized with Dowex 50W (H^+^) resin, filtered, and concentrated in vacuo. The product was purified by silica gel column chromatography to give **2** as a white solid (12.1 mg, 67%). TLC: R_f_ = 0.38 (CHCl_3_/MeOH 4:0.5). ^1^H NMR (600 MHz, MeOD:CDCl_3_ 1:3): δ 5.66 (dt, J = 14.2, 6.7 Hz, 1H), 5.38 (dd, J = 15.3, 7.7 Hz, 1H), 4.31 (d, J = 8.3 Hz, 1H, anomeric), 4.06 (t, J = 7.8 Hz, 1H), 3.98 (dd, J = 10.1, 4.1 Hz, 1H), 3.93–3.79 (m, 2H), 3.75–3.57 (m, 3H), 3.40 (dd, J = 10.2, 8.7 Hz, 1H), 3.36–3.29 (m, 1H), 3.25 (ddd, J = 9.4, 6.0, 2.6 Hz, 1H), 2.55–2.33 (m, 5H), 2.18–2.10 (m, 1H), 2.09–2.03 (m, 1H), 2.02 (bs, 1H, acetylene), 2.01–1.89 (m, 2H), 1.59–1.49 (m, 2H), 1.48–1.13 (m, 46H), 1.06–0.99 (m, 1H), 0.86–0.81 (m, 6H). ^13^C NMR (151 MHz, MeOD): δ 175.0, 173.9, 134.8, 129.5, 101.4, 82.9, 76.5, 75.0, 71.9, 71.3, 69.5, 68.1, 62.0, 56.3, 53.6, 45.4, 36.8, 35.5, 33.2, 32.7, 32.2, 31.9, 30.1, 30.0, 30.0, 29.9, 29.8, 29.8, 29.8, 29.8, 29.7, 29.7, 29.7, 29.6, 29.6, 29.6, 29.5, 29.2, 26.3, 24.1, 24.1, 22.9, 22.8, 15.1, 14.2. HRMS (ESI-TOF) m/z: [M + H]^+^ Calcd for C_47_H_85_N_4_O_8_ 833.6362; Found 833.6382.

### (2S,3R,E)-2-(tert-Butoxycarbonyl)amino-3-pivaloyloxyoctadec-4-en-1-yl 3,4,6-tri-O-acetyl-2-deoxy-2-(pent-4-ynamido)-β-D-galactopyranosyl-(1→3)-[3,4,6-tri-O-acetyl-2-deoxy-2-(pent-4-ynamido)-β-D-galactopyranosyl-(1→4)]-2,6-di-O-acetyl-β-D-galactopyranosyl-(1→4)-2,3,6-tri-O-acetyl-β-D-glucopyranoside (13)

Compound **13** (14.1 mg, 61%) was synthesized from **12** (27 mg, 0.016 mmol) by the same procedure and conditions employed for the synthesis of **9**. TLC: R_f_ = 0.7 (EtOAc). ^1^H NMR (600 MHz, CDCl_3_): δ 6.90 (d, J = 9.0 Hz, 1H, -NH), 5.89 (d, J = 7.8 Hz, 1H, -NH), 5.76 (dt, J = 14.3, 6.6 Hz, 1H, -C=CH), 5.39 (d, J = 3.0 Hz, 1H), 5.35 (d, J = 3.5 Hz, 1H), 5.33 (d, J = 7.3 Hz, 1H, -NH), 5.26 (d, J = 8.7 Hz, 1H, anomeric-H””), 5.21–5.16 (m, 2H), 5.11 (t, J = 9.5 Hz, 1H), 4.97–4.83 (m, 2H), 4.67 (d, J = 9.5 Hz, 1H, -NH), 4.55 (d, J = 8.0 Hz, 1H, anomeric-H’’’), 4.46–4.34 (m, 2H, anomeric-1H”), 4.30–4.08 (m, 8H, anomeic-1H), 4.09–3.99 (m, 3H), 3.99–3.81 (m, 4H), 3.70 (t, J = 9.5 Hz, 1H), 3.63 (dd, J = 10.0, 3.0 Hz, 1H), 3.61–3.58 (m, 1H), 3.57–3.54 (m, 1H), 3.47 (dd, J = 9.7, 4.4 Hz, 1H), 2.71–2.56 (m, 3H), 2.55–2.40 (m, 6H), 2.37–2.28 (m, 1H), 2.19 (s, 6H, 2 × CH_3_), 2.12 (s, 3H), 2.10 (s, 3H), 2.07 (s, 3H), 2.06 (s, 3H), 2.05 (s, 6H), 2.02 (s, 3H), 2.02 (s, 3H), 2.00 (s, 3H), 1.97–1.95 (m, 2H), 1.41 (s, 9H), 1.35–1.21 (m, 22H), 1.16 (s, 9H), 0.88 (t, J = 7.0 Hz, 3H). ^13^C NMR (151 MHz, CDCl_3_): δ 176.9, 174.0, 172.2, 172.1, 171.0, 170.9, 170.7, 170.6, 170.5, 170.3, 170.0, 169.8, 168.7, 155.3, 137.1, 124.6, 102.6, 101.2, 100.4, 100.1, 84.0, 83.2, 79.6, 79.1, 74.7, 73.2, 73.0, 72.4, 72.2, 71.8, 71.3, 71.2, 71.0, 70.6, 69.4, 69.3, 68.7, 68.6, 66.79, 66.6, 63.4, 62.3, 61.6, 61.0, 52.2, 51.5, 50.2, 38.9, 35.4, 35.0, 32.7, 32.4, 32.0, 29.8, 29.6, 29.62, 29.5, 29.3, 29.0, 28.4, 27.1, 22.8, 20.9, 20.9, 20.9, 20.8, 20.8, 20.7, 20.7, 14.9, 14.6, 14.3, 14.2. HRMS (ESI-TOF) m/z: [M + H]^+^ Calcd for C_84_H_125_N_3_O_36_ 1751.8116; Found 1751.8186.

### (2S,3R,E)-2-[11-(3-hexyl-3H-diazirin-3-yl)undecanamido]-3-pivaloyloxyoctadec-4-en-1-yl 3,4,6-tri-O-acetyl-2-deoxy-2-(pent-4-ynamido)-β-D-galactopyranosyl-(1→3)-[3,4,6-tri-O-acetyl-2-deoxy-2-(pent-4-ynamido)-β-D-galactopyranosyl-(1→4)]-2,6-di-O-acetyl-β-D-galactopyranosyl-(1→4)-2,3,6-tri-O-acetyl-β-D-glucopyranoside (14)

Compound **14** (8.2 mg, 62%) was synthesized from **13** (12 mg, 0.006 mmol) by the same procedure and conditions employed for the synthesis of **11**. TLC: R_f_ = 0.7 (EtOAc). ^1^H NMR (600 MHz, CDCl_3_): δ 6.90 (d, J = 9.0 Hz, 1H, -NH), 5.85 (d, J = 7.9 Hz, 1H, -NH), 5.75 (dt, J = 14.4, 6.7 Hz, 1H, -C=CH-), 5.63 (d, J = 9.3 Hz, 1H, -NH), 5.39 (d, J = 2.7 Hz, 1H), 5.36 (d, J = 2.6 Hz, 1H), 5.33 (d, J = 7.5 Hz, 1H), 5.25 (d, J = 8.6 Hz, 1H), 5.21 (t, J = 7.4 Hz, 1H), 5.19–5.15 (m, 2H), 5.12 (t, J = 9.5 Hz, 1H), 4.93–4.83 (m, 2H), 4.53 (d, J = 7.9 Hz, 1H, anomeric), 4.40 (d, J = 10.8 Hz, 1H), 4.38 (d, J = 7.8 Hz, 1H, anomeric), 4.35–4.30 (m, 1H), 4.29–4.10 (m, 8H), 4.08–3.99 (m, 3H), 3.94–3.86 (m, 3H), 3.70 (t, J = 9.6 Hz, 1H), 3.66–3.62 (m, 1H), 3.62–3.58 (m, 1H), 3.57–3.51 (m, 1H), 3.48 (dd, J = 4.5 Hz, 1H), 2.67–2.58 (m, 2H), 2.56–2.41 (m, 5H), 2.39–2.26 (m, 2H), 2.19 (s, 3H), 2.17 (s, 3H), 2.12 (s, 3H), 2.10 (s, 3H), 2.08 (s, 3H), 2.06 (s, 3H), 2.05 (s, 3H), 2.04 (s, 3H), 2.03 (s, 3H), 2.02 (s, 3H), 2.00 (s, 3H), 1.98–1.93 (m, 2H), 1.54–1.47 (m, 1H), 1.36–1.19 (m, 47H), 1.16 (s, 9H), 1.08–1.03 (m, 1H) 0.88 (t, J = 7.0 Hz, 6H). ^13^C NMR (151 MHz, CDCl_3_): δ 177.0, 172.6, 172.1, 172.0, 171.0, 170.9, 170.7, 170.4, 170.2, 170.0, 169.8, 168.7, 168.6, 159.4, 159.3, 137.2, 125.0, 102.7, 100.9, 100.4, 100.0, 84.0, 83.2, 79.1, 73.1, 72.4, 72.2, 71.3, 71.1, 70.6, 69.4, 68.7, 68.6, 67.7, 66.6, 63.4, 62.3, 61.6, 61.0, 51.5, 50.5, 50.1, 38.9, 36.9, 35.4, 35.0, 34.8, 33.0, 32.4, 32.0, 31.7, 31.0, 29.8, 29.6, 29.5, 29.3, 29.3, 29.1, 29.0, 28.5, 27.1, 25.8, 25.4, 24.0, 22.8, 22.8, 21.2, 20.9, 20.9, 20.9, 20.8, 20.7, 20.7, 14.9, 14.6, 14.2, 14.1. HRMS (ESI-TOF) m/z: [M + H]^+^ Calcd for C_97_H_149_N_5_O_35_ 1944.0106; Found 1944.0197.

### (2S,3R,E)-2-[11-(3-hexyl-3H-diazirin-3-yl)undecanamido]-3-hydroxyoctadec-4-en-1-yl 2-deoxy-2-(pent-4-ynamido)-β-D-galactopyranosyl-(1→3)-[2-deoxy-2-(pent-4-ynamido)-β-D-galactopyranosyl-(1→4)]-β-D-galactopyranosyl-(1→4)-β-D-glucopyranoside (1)

Compound **1** (4.1 mg, 69%) was prepared from **14** (8.2 mg, 0.004 mmol) by the same procedure and conditions used for the synthesis of **2**. TLC: R_f_ = 0.4 (MeOH:CHCl_3_ 1:1). ^1^H NMR (600 MHz, MeOD:CDCl_3_ 1:1): δ 5.66 (dt, J = 14.3, 6.8 Hz, 1H), 5.42 (dd, J = 15.3, 7.6 Hz, 1H), 4.89 (d, J = 8.6 Hz, 1H, anomeric), 4.50 (d, J = 8.4 Hz, 1H, anomeric), 4.33 (d, J = 7.6 Hz, 1H, anomeric), 4.29 (d, J = 2.5 Hz, 1H), 4.25 (d, J = 7.7 Hz, 1H, anomeric), 4.16 (dd, J = 9.9, 4.1 Hz, 1H), 4.05 (t, J = 7.8 Hz, 1H), 3.96–3.90 (m, 3H), 3.86–3.76 (m, 7H), 3.73–3.66 (m, 2H), 3.64 (dd, J = 11.6, 5.6 Hz, 1H), 3.62–3.57 (m, 2H), 3.57–3.46 (m, 8H), 3.34 (ddd, J = 10.2, 4.9, 2.4 Hz, 1H), 3.29–3.26 (m, 1H), 2.56–2.38 (m, 8H), 2.14 (t, J = 7.6 Hz, 2H), 2.10–2.06 (m, 2H), 2.02–1.96 (m, 2H), 1.60–1.51 (m, 2H), 1.43–1.14 (m, 46H), 1.08–1.00 (m, 2H), 0.85 (t, J = 7.0 Hz, 3H). ^13^C NMR (151 MHz, MeOD): δ 174.7, 154.8, 154.7, 134.9, 129.9, 104.2, 104.0, 103.5, 102.3, 83.6, 83.4, 79.2, 75.8, 75.7, 75.5, 75.15, 74.5, 73.9, 73.1, 72.5, 72.4, 70.3, 69.6, 69.5, 69.2, 69.1, 68.9, 62.3, 62.2, 61.0, 60.5, 54.2, 53.9, 53.8, 53.4, 36.9, 35.5, 33.3, 32.8, 32.4, 30.1, 30.1, 30.0, 29.9, 29.8, 29.7, 29.3, 28.6, 26.4, 24.32, 24.2, 23.1, 22.9, 15.1, 14.2. HRMS (ESI-TOF) m/z: [M + H]^+^ Calcd for C_70_H_119_N_5_O_23_ 1398.8369; Found 1398.8416.

### Cell culture

HEK293 cells were cultured in high glucose DMEM supplemented with 10% (V/V) FBS, 100 μg/ml streptomycin, and 100 U/ml penicillin at 37°C in a 5% CO_2_ incubator maintaining a water-saturated atmosphere. HEK293 cells of passage four were used for various studies.

### Fluorescence imaging of cells

HEK293 cells (50 × 10^3^) were seeded in poly-L-lysine (1% solution in DPBS)-coated 35 mm dish and were allowed to grow to ∼60% confluence. Cells were washed three times with DPBS and incubated with RPMI buffer (1 ml) containing 50 μM of **2** (24.43 μl from 2.05 mM stock solution in DMSO) or **1** (18.94 μl from 2.64 mM stock solution in DMSO), respectively. For the negative control, cells were incubated with DPBS only. After 3 h of incubation, cells were washed with DPBS (0.5 ml) three times, and DPBS (1 ml) was added to each dish. Cells were exposed to UV irradiation (365 nm wavelength) at 4°C for 15 min using a Spectroline UV lamp (Spectroline, ENF-280C, 120 V, 60 Hz, 0.20 Amps), which was followed by washing. Cells were incubated with 4% PFA in DPBS at rt for 15 min and rinsed with DPBS (3 × 1 ml). The fixed cells were treated with click master mix (50 μM Biotin-Azide, 50 mM THPTA, 4.75 mM sodium ascorbate, and 2 mM CuSO_4_) at rt for 1 h as described in the literature (60). Cells were washed with DPBS (3 × 500 μl), 500 mM aq. NaCl solution (3 × 500 μl), and double-distilled water. Cells were incubated with streptavidin-A488 (1:1,000 dilution of 1 mg/ml stock) in 1 ml of DPBS for 30 min in the dark. After washing with DPBS, cells were incubated with DAPI (50 nM, 1 ml for each dish) at rt for 5 min. Finally, the cells were washed with DPBS and subjected to fluorescent imaging.

For organelle localization study, HEK293 cells were treated with streptavidin-A488 by the same procedure. The cells were washed, fixed, and permeabilized with cell permeabilization buffer for 15 min, followed by washing and incubation with fluorophore-conjugated organelle antibody markers (in a final concentration of 2 μg/ml for each antibody) in 1 ml of DPBS containing 1.5% BSA at rt for 1 h with gentle shaking. Finally, the coverslip containing cells was washed and mounted on the microscopic glass slide using the mounting media.

### Flow cytometry analysis of cells

After treatment with **1** or **2** (200 μM), using DPBS as control, for different periods (1, 2, 3, 4, 6, and 12 h), cells were pelleted by centrifugation (600 *g*) at 4°C for 8 min, suspended in DPBS (1×, 200 μl) containing 100 μM of biotin-azide, and incubated on ice for 45 min as mentioned above. Cells were washed with ice-cold PBS (1.0 ml) and pelleted by centrifugation, which was repeated three times. The cells were suspended in PBS (100 μl) and incubated with A488-streptavidin (1:500 dilution) on ice in the dark for 30 min. The cells were centrifuged and washed with ice-cold DPBS (500 μl), which was repeated three times. Finally, the cells were resuspended in ice-cold PBS (200 μl) and subjected to FACS analysis using a blue (488 nm) excitation laser and 530 nm emission filter. Data were analyzed using the Attune NXT software.

### Labeling proteins in live HEK293 cells using 1 and 2

HEK293 cells (0.8 × 10^6^) were seeded onto a 100 mm tissue culture dish as mentioned above and were allowed to grow to 90% confluence. The cells were harvested, pelleted, and resuspended in serum-free media (7 ml) with a final cell count of ∼4.7 × 10^6^. Cells were equally divided into three centrifuge tubes, washed with DPBS three times, replenished with fresh serum-free media containing 200 μM of **1**, **2**, or PBS (negative control), and transferred onto a 35 mm tissue culture dish. Following incubation at 37°C for 4 h, cells were washed with DPBS, resuspended in DPBS (1 ml), and exposed to 365 nm UV light as described. Thereafter, cells were pelleted through centrifugation (800 *g*, 4°C, 6 min), washed with cold DPBS (2×), and aspirated. Cell pellets were either stored at −80°C until use or directly applied to the next step.

### Western blot analysis of labeled proteins

These experiments followed our previous protocols (60, 61). In short, the cell pellets obtained above were lysed in ice-cold lysis buffer (500 μl) containing 5.0 μM protease inhibitor (Halt protease inhibitor cocktail, Thermo Scientific) on a Qsonica probe sonicator (6 pulses, 60% duty cycle, 30 s each, Amp 10). The whole cell lysate (WCL) was subjected to protein precipitation by adding cold MeOH (2 ml), CHCl_3_ (0.5 ml), and water (3.5 ml) (4/1/7, v/v/v), followed by mixing and centrifuging at 21,000 *g* for 20 min. The supernatant was discarded, and this precipitation process was repeated two more times. The resulting protein pellet was dried at rt, resuspended in PBS, and analyzed to determine protein concentration using a bicinchoninic acid (BCA) protein assay kit (Thermo Scientific) following the manufacturer’s instructions. An aliquot of ca. 50 μg proteins was put in a 1.5 ml centrifuge tube and mixed with freshly prepared click master mix (50 μM Biotin-Azide, 50 mM THPTA, 4.75 mM sodium ascorbate, and 2 mM CuSO_4_) at rt for 1 h. Each reaction mixture was made up to 50 μl of final volume by adding DPBS and mixed by vortexing. The reaction was kept at rt for 1 h before being quenched with 50 μl ice-cold MeOH. Cold DPBS (50 μl) was added to the mixture, followed by cold MeOH (150 μl), CHCl_3_ (50 μl), and water (300 μl). The cloudy solution was vortexed and then centrifuged (21,000 *g*, 4°C, 20 min) to separate proteins from the aqueous and organic layers, and the protein fraction was washed with cold MeOH (3×). The pelleted proteins were dried at rt, resuspended in SDS lysis buffer (100 μl), and sonicated in a water bath until they were dissolved. Protein concentration was measured using a BCA protein assay kit. Proteins (25 μg/gel lane) were mixed with SDS loading buffer (4× stock), boiled at 95°C, loaded in SDS-PAGE gels containing 10% acrylamide, and developed. The protein gel was transferred onto the PVDF membrane that was washed with PBS, incubated with blocking solution (10 ml) at rt for 1 h, washed with PBS again, and then incubated with streptavidin-alkaline phosphatase (AP) (1 μg/ml in PBS) at rt for 45 min with gentle shaking. The membrane was washed with PBS, and the protein bands were detected by incubating with 5-bromo-4-chloro-3-indolyl phosphate (BCIP)/nitro blue tetrazolium (NBT) chromogenic substrate (1 tablet dissolved in 7 ml of PBS) for 5 min before being photographed.

### MS/MS-based proteomic analysis

Cell lysis, tagging of the labeled proteins with biotin using biotin-azide instead of Cy5-azide for the click reaction, and protein isolation from cell lysate followed the above-described protocols. The protein fractions were washed with MeOH, pelleted, and then resuspended in a freshly prepared pre-equilibrated solution of streptavidin Agarose resin in DPBS (300 μl) at rt for 2 h with end-to-end rotation. The streptavidin beads were separated by centrifugation (1,500 *g*, 2 min) and washed with 0.2% SDS in DPBS (3 × 2 ml) and H_2_O (3 × 2 ml). The beads were finally applied to an MS/MS-based proteomic study. MS sample preparation and analysis conditions are the following. *1*) On-bead trypsin digestion of proteins. Protein-loaded beads obtained above were diluted with 50 mM ammonium bicarbonate and then treated with 4 mM dithiothreitol (DTT) at 65°C for 15 min, 10 mM chloroacetamide (CAA) at rt for 30 min in the dark. Next, the beads were treated with trypsin (500 ng) at 37°C overnight. Tryptic peptides were desalted with ZipTip following the manufacture's protocol (Millipore Sigma). The peptides were lyophilized at 160 mBar with a speed vac and resuspended in 0.1% formic acid (FA) for liquid chromatography-tandem mass spectrometry (LC-MS/MS) analysis. Two valid replicates for each genotype were prepared for proteomic study. *2*) LC-MS/MS-based proteomic study. Proteomic data acquisition was achieved on an EASY-nLC™ 1200 System coupled with Orbitrap Fusion™ Mass Spectrometers (Thermo Fisher Scientific). Samples were loaded to a PepMap® 100 C18 trapping column (75 μm i.d. × 2 cm, 3 μm, 100 Å) and separated on a PepMap® C18 analytical column (75 μm i.d. × 25 cm, 2 μm, 100 Å). The flow rate was set at 250 nl/min with solvent A (0.1% FA in water) and solvent B (0.1% FA and 80% acetonitrile in water) as the mobile phases. Separation was conducted using the gradient of 2%–35% of B over 0–70 min; 35%–80% of B over 70–75 min; 80%–98% of B over 75–76 min, and isocratic at 98% of B over 76–90 min. For MS data acquisition, the full MS1 scan (m/z 350–1,800) was performed on the Orbitrap with a resolution of 120,000. The automatic gain control (AGC) target is 2e5 with a maximum injection time of 50 ms. Peptides bearing +2–6 charges were selected with an intensity threshold of 1e4. Dynamic exclusion of 30 s was used to prevent resampling the high abundance peptides, and the quadrupole isolation window was 1.3 Th. Fragmentation of the top 10 selected peptides by collision-induced dissociation was done at 35% of normalized collision energy. The MS2 spectra were acquired at the Ion Trap with AGC target as 1e4 and maximum injection time as 35 ms.

### Analysis of MS/MS data

Proteome DiscovererTM (version 2.5, Thermo Scientific) was used to search the MS/MS spectra from the protein samples. The SEQUEST algorithm in the Proteome Discoverer was used to process raw data. Spectra were searched using the Uniprot *Homo sapiens* protein database with the following parameters: 10 ppm mass tolerance for MS1 and 0.6 for MS2, two maximum missed tryptic cleavage sites, a fixed modification of carbamidomethylation (+57.021) on cysteine residues, and dynamic modifications of oxidation of methionine (+15.996). Search results were filtered at 1% false discovery rate (FDR) and at least two unique peptides per protein for protein identification. Relative protein abundance in the samples was measured using label-free quantification, and proteins identified and quantified in all biological samples were used. No imputation was performed. Peptides in samples were quantified as areas under the chromatogram peak. FDR cutoffs for both peptide and protein identification were set as 1%.

### Experimental validation of candidate GSL-binding proteins

HEK293 cells (∼0.8 × 10^6^) were seeded in a poly-L-lysine-coated 60 mm tissue culture dish with 5 ml of cell culture media and grown until ∼80% confluence for transfection. The transfection reagent was prepared according to the manufacturer’s protocols using the recommended concentration of lipofectamine agent. In short, on the day of transfection, 2.5 μg of plasmid DNA was diluted in 600 μl of OptiMEM media, and 10 μl of lipofectamine 2000™ transfection reagent was separately diluted in 600 μl of OptiMEM media containing enhancer reagent (2 μl). The two solutions were mixed and incubated at rt for 45 min with occasional mixing using a pipette. In the meantime, cells were washed with OptiMEM media (1 ml) three times and incubated with the DNA/lipofectamine reagent mixture in 2.5 ml of optiMEM media for 5 h. Thereafter, 2.5 ml of serum-containing cell culture media was added to the cells. After 12 h of incubation, the cell culture media was discarded and replenished with 5 ml of serum-containing media for 36 h. The transfection efficiency was validated and optimized by analyzing the proteins extracted from the WCL using SDS-PAGE as mentioned above and blotting against the anti-protein antibody or anti-FLAG tag antibody. The generated transfected cells were incubated with **1**, **2**, or PBS (control) and then exposed to UV irradiation following the protocol mentioned above. Cells were collected by centrifugation, washed with cold DPBS, and lysed by resuspending the pellet in 1 ml of cell lysis buffer containing 10 μl of protease inhibitor cocktail using a Qsonica probe sonicator (6 pulses, 60% duty cycle, 30 s each, Amp10). Cell lysate was subjected to protein extraction following the above protocol. Protein pellet was resuspended in DPBS, and protein concentration was measured using a BCA assay kit. Proteins (∼500 μg) were placed in a separate centrifuge tube and subjected to click reaction with biotin-azide as mentioned above. The reaction was quenched by adding cold methanol, and the proteins were extracted. The protein pellet was resuspended in DPBS with protein concentration measured with a BCA assay kit. Thereafter, ∼250 μg of proteins was diluted in cell lysis buffer (final volume of 300 μl) and subjected to anti-FLAG magnetic agarose bead-mediated purification according to the manufacturer’s protocol. Thus, 50 μl of the magnetic bead slurry (25% slurry stock dissolved in DPBS containing 0.01% Tween-20% and 0.02% sodium azide, pH 7.2) was transferred into a 1.5 ml centrifuge tube and washed with cell lysis buffer (450 μl) three times. The tube was placed on a magnetic stand to collect the beads while discarding the supernatant. This bead-washing process was repeated four more times. Next, protein samples (300 μl) were added to the washed beads, followed by vortexing and incubation at rt for 45 min. The beads were collected with a magnetic stand and washed with cell lysis buffer three times and protein washing buffer (1× DPBS containing 150 mM NaCl, pH 7.2) three times. Thereafter, 50 μl of SDS-PAGE buffer containing 2.5 μl of 2M DTT was added to each tube, and the sample was heated at 95°C for 15 min. The magnetic beads were separated from the solution using a magnetic stand. An aliquot of the supernatant was subjected to SDS-PAGE and Coomassie blue staining for visualization, whereas another aliquot of the supernatant (∼25 μl) was subjected to SDS-PAGE and fluorescence analysis employing Cy5-streptavidin conjugate solution (1 μg/ml in DPBS) according to conventional protocols.

### Statistical analysis

Statistical analysis of data was achieved with the GraphPad Prism 9.0 software. Results are presented as the mean ± standard deviation and are compared with a two-tailed student’s *t* test. ∗*P* <0.05, ∗∗*P* <0.01, ∗∗∗*P* <0.001, and ∗∗∗∗*P* <0.0001 show different levels of statistical significance.

## Results

### Research design

**Fig. 2:**
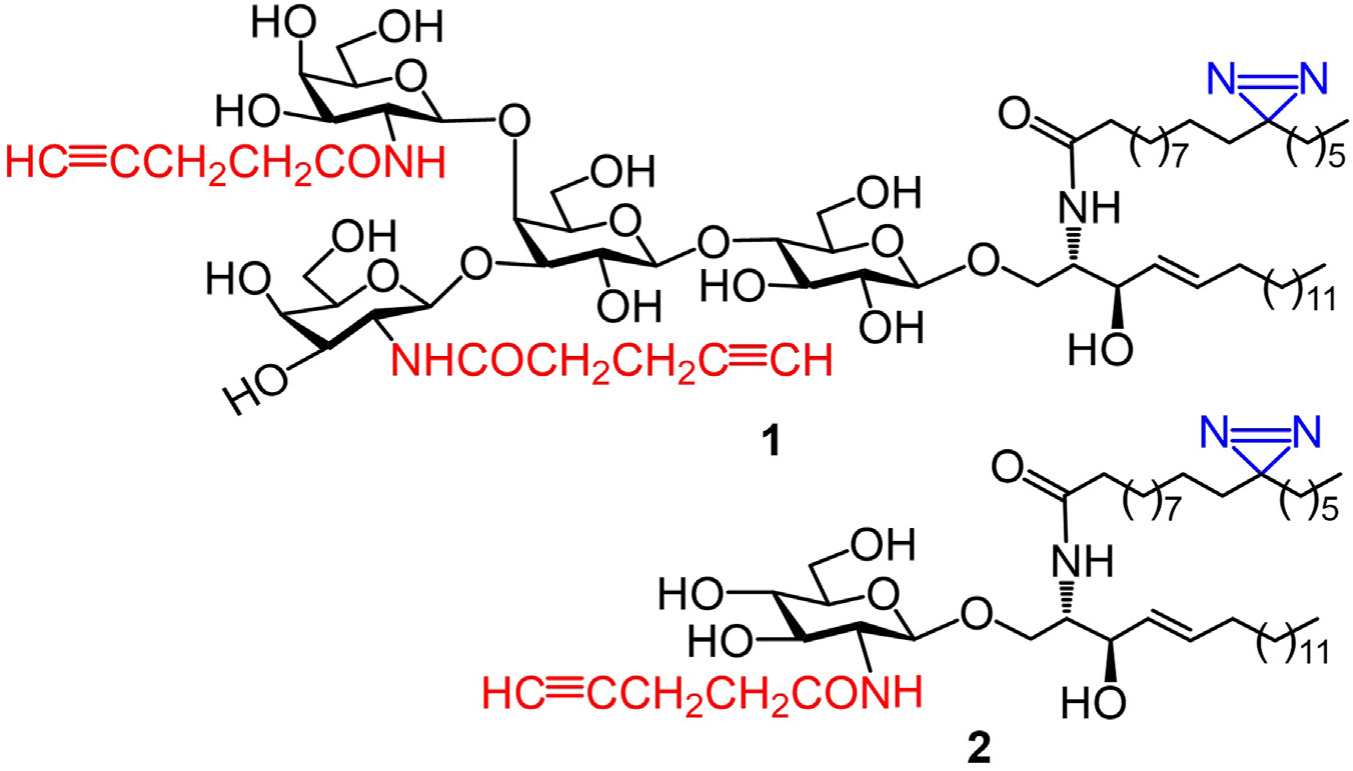
Structures of the designed GSL probes **1** and **2**.

To study GSL-cell membrane interactions and identify GSL-binding membrane proteins, it is necessary to have probes that not only label GSL-binding proteins but also help isolate them. In this context, we have designed and synthesized bifunctional derivatives **1** and **2** (Fig. 2) of II^3^-β-(*N*-acetyl-D-galactosamine)-GA2 (GalNAc-GA2), which is an epimer of LcGg4, and β-(*N*-acetyl-D-glucosamine)-ceramide (GlcNAc-Cer), respectively. The latter is utilized as a negative control because GlcNAc-Cer linkage is not found in mammalian GSLs yet. However, these two probes contain the same lipid tail, which carries a photoreactive diazirine in the fatty acyl moiety that can be activated with 365 nm UV light to yield a carbene to react with proximal molecules and generate a covalent linkage. This will enable **1** and **2** attachment to transmembrane proteins close to or interacting with their lipid moiety and, hence, label the targeted proteins. Photo-affinity labeling has proved to be a powerful tool to investigate protein-protein/lipid interactions in live cells (62, 63, 64, 65, 66). The C18:0 stearic group in **1** and **2** is an abundant fatty acyl chain in the Cer moiety of mammalian GSLs, while its C-12, where the diazirine is located, would be in the outer leaflet of the cell membrane. In the meantime, both **1** and **2** also contain alkynes in the glycan, which can react with azides via click reactions under mild conditions for the introduction of an affinity tag to facilitate the rapid isolation of crosslinked proteins and subsequent proteomic studies. Probe **1** was designed to contain an alkyne group in each GalNAc unit, as its synthesis is easier than that of probes with alkyne on a single GalNAc residue, which needs to differentiate the two sugar units. The diazirine and alkyne groups are used to modify the fatty acyl chain of Cer and the GalNAc N-acetyl group of glycans, respectively, because they are small and are expected to have a minimal impact on the GSL structure. Thus, **1** and **2** are anticipated to be effective probes to explore GSL-membrane interaction, while the exact impact of the pent-4-ynoic group on GSL-membrane interactions can be studied by comparing **1** to probes containing unmodified glycans. Moreover, probes **1** and **2** are designed to have the diazirine and alkyne moieties separately located in the lipid and glycan. As a result, even if **1** and **2** are metabolized and their metabolites are incorporated by cells into the salvage biosynthetic pathways of GSLs and other biomolecules like lipids or glycoproteins, it is unlikely that both functional groups would end up in the same biosynthetic products for being labeled and pulled down simultaneously. Additionally, because **1** and **2** contain the same Cer moiety but different glycans, comparing the proteins captured by these probes can provide additional information. Firstly, the results will verify the feasibility of these probes to label and identify GSL-associated membrane proteins. Secondly, the results will demonstrate how the glycan structure in GSLs influences their interactions with cell membranes and other functions.

**Fig. 3:**
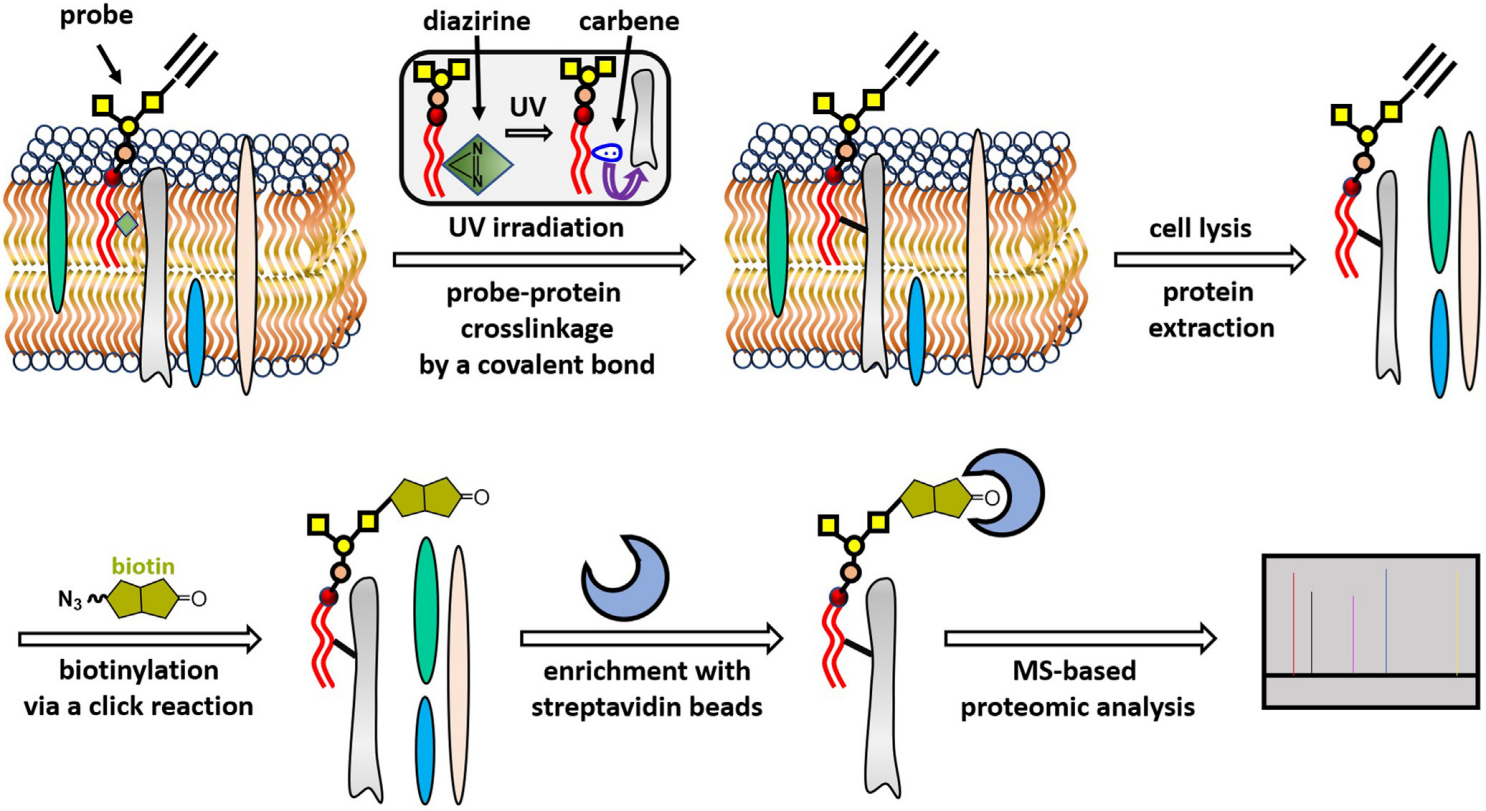
An illustration of the experimental procedures designed to label and isolate GSL-interacting proteins in the cell membrane for proteomic study. Synthetic GSL probes are spontaneously integrated into the cell membrane after incubation with cells. Upon UV radiation of the cells, a reactive carbene is generated in the lipid chain of the probe, which reacts with proteins nearby to form a covalent bond. Click reaction between the alkyne group in the probe and azide-modified biotin labels the crosslinked proteins with a biotin tag to facilitate their isolation utilizing streptavidin-modified beads. The bead-bound proteins are subjected to MS/MS-based proteomic analysis according to established protocols.

Our experimental procedures/protocols for the labeling, pull-down, and identification of GSL-interacting membrane proteins are depicted in Fig. 3. Synthetic probes **1** and **2** will be added to the cell culture for cellular incorporation into membranes, which has been verified with many synthetic glycolipids (60, 61, 67, 68). Next, the cells will be subjected to UV irradiation to initiate the cross-linkage between probes and adjacent or GSL-interacting membrane proteins, thereby labeling the targeted proteins. Subsequently, total proteins will be extracted from the cell lysates and applied to a click reaction to attach a biotin tag to the crosslinked proteins. Finally, the biotinylated proteins will be isolated with streptavidin-beads and subjected to MS/MS-based proteomic analysis by conventional protocols.

### Chemical synthesis of probes 1 and 2

**Scheme 1:**
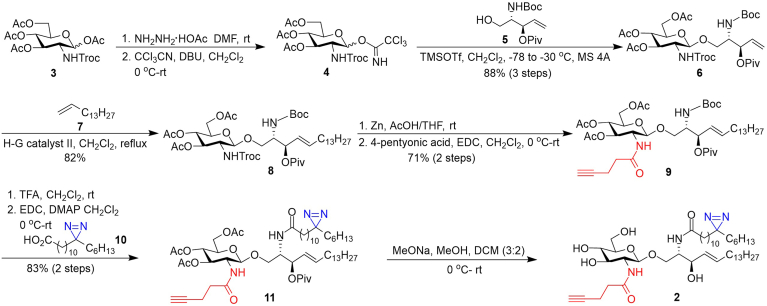
Synthesis of probe **2**.

The synthesis of probe **2**, as outlined in Scheme 1, commenced with the conversion of a fully protected glucosamine derivative **3** into imidate **4** as a glycosyl donor in two steps, including regioselective anomeric de-O-acetylation using hydrazine acetate and reaction of the resultant hemiacetal with trichloroacetonitrile in the presence of 1,8-diazabicyclo[5.4.0]undec-7-ene (DBU) under conventional conditions. Glycosylation of the sphingosine precursor **5** with **4** under the promotion of TMSOTf gave an excellent overall yield (88% for three steps) of the glycolipid precursor 6. The newly formed *β*-glycosidic linkage in **6** was confirmed by the large coupling constant (*J* = 7.5 Hz) of the anomeric ^1^H signal (δ 4.59 ppm) in its ^1^H NMR spectrum. Thereafter, **6** was subjected to lipid remodeling and protecting group manipulation. Firstly, cross-metathesis of **6** and *n*-pentadecene **7** in the presence of the second-generation Hoveyda-Grubbs catalyst (3 mol%) in DCM gave the desired *Z*-olefin **8** (82%), which was confirmed by the coupling constant (*J* = 15.3 Hz) of its vinyl protons. This reaction was slow, taking approximately 5 days to complete, but clean. Next, the *N*-2,2,2-trichloroethoxycarbonyl (Troc) group in 8 was selectively removed using Zn/AcOH, which was followed by selective N-acylation of the resultant amine using 4-pentynoic acid and EDC to provide **9** in a 71% yield (two steps). The successful incorporation of an alkyne group in 9 was verified by its ^1^H NMR spectrum, showing the terminal alkyne proton as a triplet (*J* = 2.5 Hz) at δ 1.99 ppm. Subsequently, the *N-tert*-butyloxycarbonyl (Boc) group in **9** was removed with 4% TFA in DCM, and the resultant amine was acylated using C-12-diazirine-modified stearic acid **10** (67, 68), EDC, and DMAP to provide **11** in an 83% yield. Finally, all *O*-acyl groups in **11** were removed with NaOMe (5 M) to afford 2 in a 67% yield. The diazirine moiety was proved to be stable to the deprotection conditions.

**Scheme 2:**
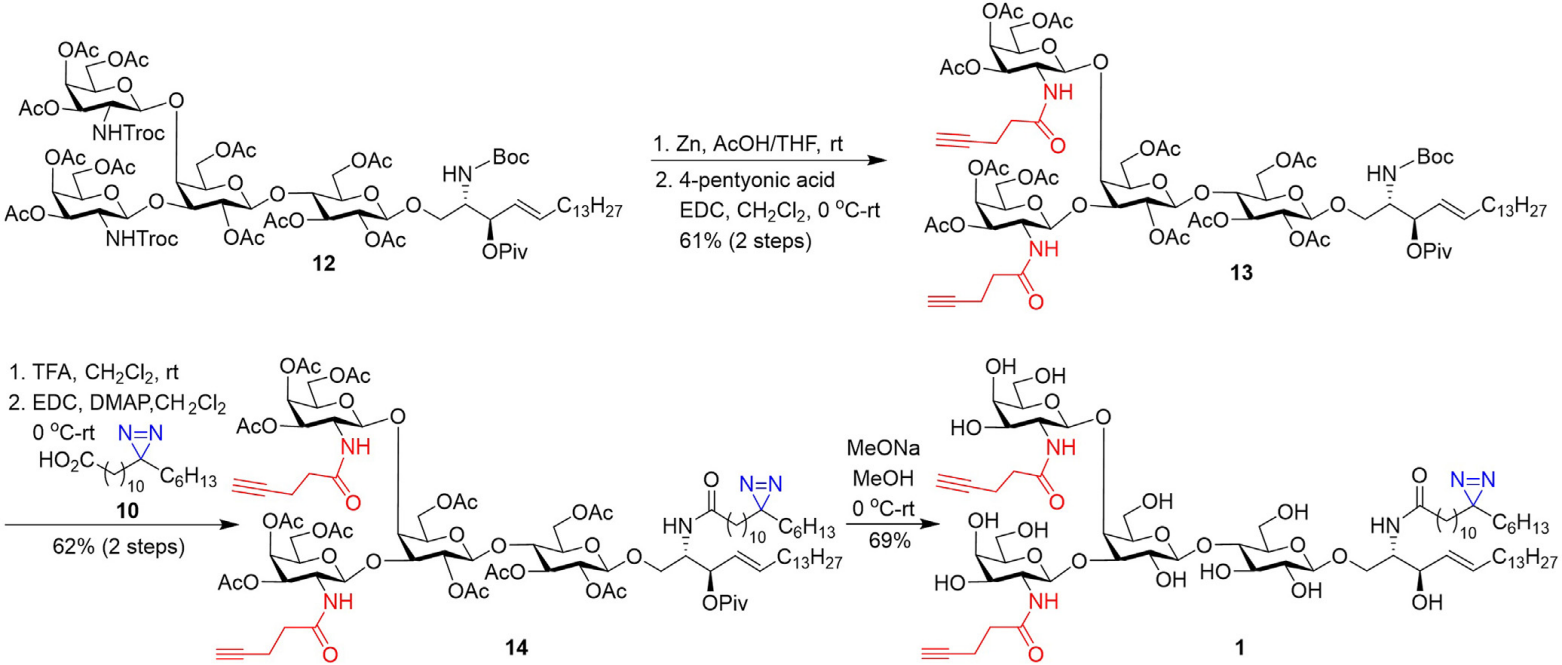
Synthesis of probe **1**.

Probe 1 was synthesized by the procedure outlined in Scheme 2. First, we prepared the key intermediate **12** according to a method developed for the synthesis of LcGg4 and its derivatives (69). Next, as described above, the *N*-Troc groups in **12** were selectively removed using Zn/AcOH, which was followed by N-acylation using 4-pentynoic acid, EDC, and DMAP to provide **13** in a 61% yield. Similarly, the diazirine-modified fatty acyl chain was attached to the lipid moiety using **10** in the presence of EDC and DMAP, after the Boc group in **13** was removed with TFA, to afford **14**. Finally, global deprotection of **14** with NaOMe provided **2** in a good yield (69%). Both **1** and **2**, as well as the synthetic intermediates, were fully characterized with NMR and HRMS data.

### Cell incorporation of probes 1 and 2

**Fig. 4:**
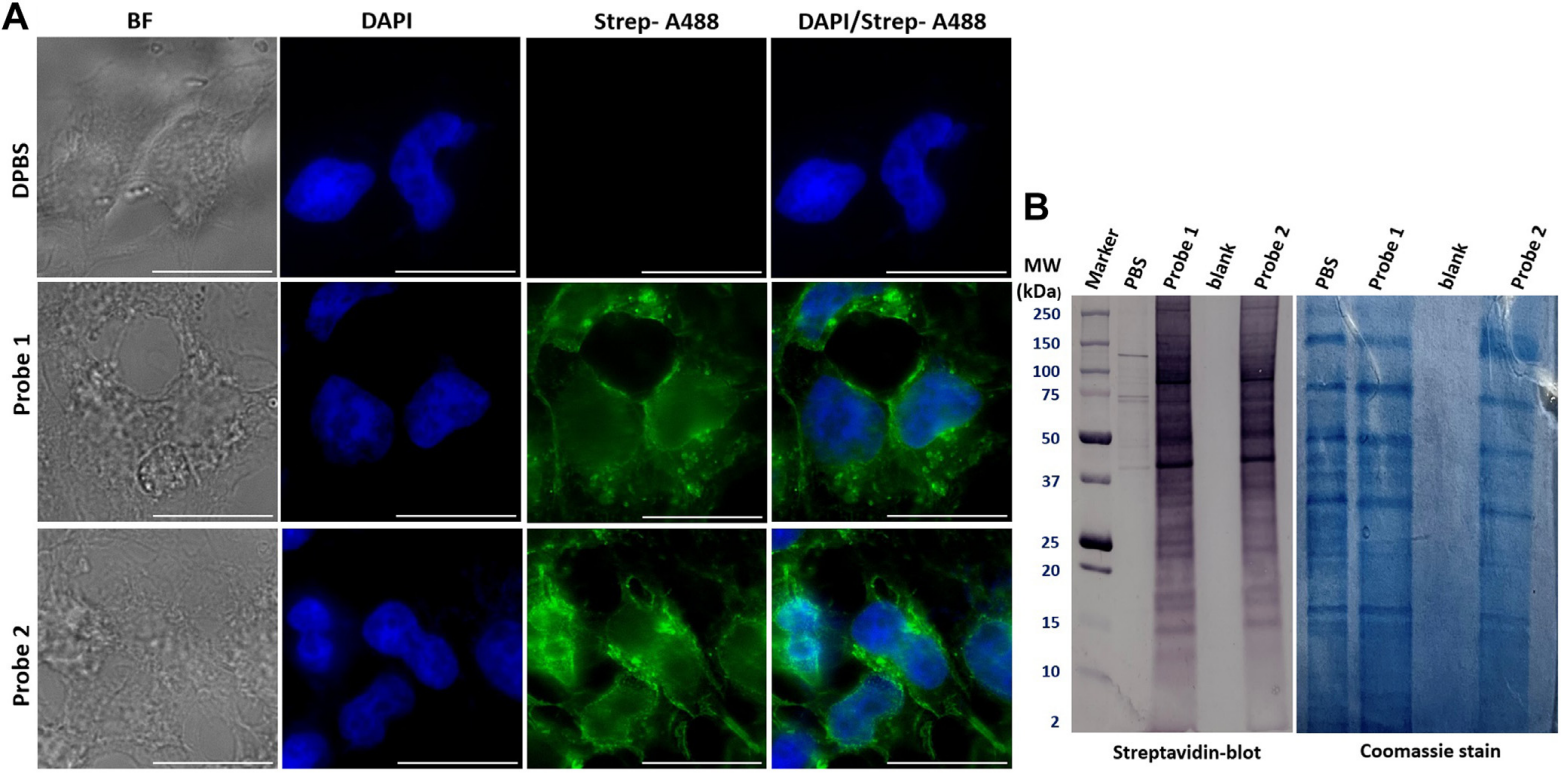
A: Bright-field (BF), 4′,6-diamidino-2-phenylindole (DAPI), streptavidin-A488 (Strep-A488), and Strep-A488/DAPI overlay fluorescent images of HEK293 cells treated with DPBS (negative control), **1**, and **2**, respectively, followed by treatments with azide-modified biotin, Streptavidin-A488, and finally DAPI to stain DNAs in the cell nucleus. The scale bar is 20 μm. B: Western blot (left) and Coomassie blue staining (right) results of proteins extracted from HEK293 cells treated with **1**, **2**, or DPBS buffer (negative control), then with biotin-azide, and finally run by SDS-PAGE and stained with streptavidin-AP and then with BCIP/NBT (left) or Coomassie blue (right).

The human embryonic kidney (HEK) 293 cell was used in this study since it has been commonly used for protein overexpression and related studies and its proteomic information is easily accessible (70). For example, currently, there are several reports about quantitative and qualitative proteomics (71, 72) and GSL analyses (73) of HEK293 cells. To verify the effective incorporation of **1** and **2** in HEK293 cell membranes, we performed fluorescence labeling and flow cytometry (FACS) study of cells. In these experiments, HEK293 cells were incubated with **1** and **2** for different periods (1–12 h) and washed. Then, the cells were sequentially treated with azide-modified biotin for biotinylation by copper-catalyzed alkyne-azide cycloaddition (CuAAC), a click reaction, and streptavidin-A488 (a green fluorophore). Finally, fluorescence intensities of the treated cells were analyzed with FACS to prove that **1** and **2** were efficiently incorporated by HEK293 cells within 2–3 h (supplemental Fig. S1). No further significant increase in the fluorescence intensity was observed after 12 h of incubation, which agreed with previous reports (61, 74). We also utilized fluorescence microscopy to validate the incorporation of **1** and **2** by HEK293 cells. To this end, cells were incubated with the probes for 3 h, washed, exposed to UV lights for 15 min, and then treated with azide-modified biotin and streptavidin-A488 as described. Finally, the labeled cells were analyzed using a fluorescence microscope. The results (Fig. 4A) clearly show strong fluorescent labeling of cells treated with both **1** and **2**, in contrast to control cells treated with PBS instead. Therefore, both the FACS and fluorescence microscopy results indicate the efficient incorporation of **1** and **2** into the membranes by HEK293 cells.

Previous studies by our group and others indicate that besides the plasma membrane, glycolipids can also translocate into cells to interact with intracellular organelle membranes (60, 61, 75, 76, 77). To determine the locations of **1** and **2** in organelles, we studied their cellular distributions using fluorescence labeling. After cells were incubated with **1** or **2** for 1, 2, 3, and 4 h and labeled with azide-biotin and streptavidin-A488, they were fixed, permeabilized, and treated with A647-tagged anti-calreticulin, GM130, EEA1, RAB7, and LAMP1 antibodies to stain the endoplasmic reticulum (ER), Golgi, early endosome, late endosome, and lysosome (supplemental Table S1), respectively. Next, the cells were analyzed with a fluorescent microscope using the A488 and Cy5 channels to find the correlation between the two fluorescent labels. The results (supplemental Figs. S2–S11) reveal a time-dependent co-localization of **1** or **2** with intracellular organelles. Within 1 h, both **1** and **2** showed a significant overlap with ER fluorescence, suggesting their rapid incorporation into ER, which is the main destination in GSL recycling. However, such correlation declines with elongated time, possibly due to probe metabolism and redistribution to other organelles. Indeed, the correlation of **1** or **2** with other organelles is low at **1** h but increases gradually until 3 h, followed by a generally declining trend thereafter, especially for **1**. Interestingly, the overlap of both **1** and **2** with lysosome is low. These findings are further validated by the results of Pearson correlation coefficient analysis (supplemental Figs. S12 and S13). Moreover, **1** and **2** show different distributions in specific organelles, suggesting their potentially different transport and metabolism pathways due to their different glycans. Since the two probes show the most similar localization patterns at 3 h, this condition was selected for subsequent experiments. Clearly, upon incubation with cells, at least a part of **1** and **2** is localized in intracellular organelles, which is expected due to their involvement in glycolipid metabolism and recycling (77, 78). Thus, crosslinking of these probes with proteins in intracellular membranes is also expected in the following experiments. In addition, we cannot eliminate the possibility that some fluorescent signals are from other biomolecules that have incorporated alkyl GlcNAc in the metabolites of **1** and **2** through salvage pathways, but unlike **1** and **2**, they are not bifunctional to achieve simultaneous cross-linkage with membrane components and biotinylation in subsequent experiments.

### Analysis of GSL-interacting membrane proteins in live cells using probes 1 and 2

Next, we conducted experiments to investigate whether **1** and **2** could crosslink with proteins in live cells for protein pull-down and analysis. Following the procedure outlined in Fig. 3, we incubated HEK293 cells with **1** or **2** for 3 h and then exposed the cells to UV lights to allow for probe-protein crosslinking. Thereafter, the cells were lysed, and the proteins were extracted. An aliquot of the protein sample was subjected to a click reaction with azide-modified biotin to introduce a biotin tag to the probes. Finally, the proteins were precipitated to remove excess biotin and applied to sodium dodecyl sulfate-polyacrylamide gel electrophoresis (SDS-PAGE). The developed gels were treated with streptavidin-AP and BCIP/NBT for the detection of biotinylated proteins. The results (Fig. 4B) clearly indicate that both probes can label and pull down many proteins, which contrasts cells treated with only DPBS (negative control), suggesting the feasibility of using **1** and **2** to investigate GSL-membrane interactions.

Analysis of GSL-interacting proteins in the membranes of HEK293 cells was performed according to the procedure depicted in Fig. 3, using the optimized conditions established by the SDS-PAGE study. After incubation of cells with the probes and UV irradiation, cells were lysed, and the cell lysates were subjected to protein precipitation, click reaction with biotin-azide, and then protein precipitation as described earlier. The proteins were dissolved in SDS buffer and incubated with streptavidin beads. The beads were isolated, washed, and finally subjected to MS/MS-based proteomic analysis following well-established protocols. Each experiment was repeated three times to verify the results.

**Fig. 5:**
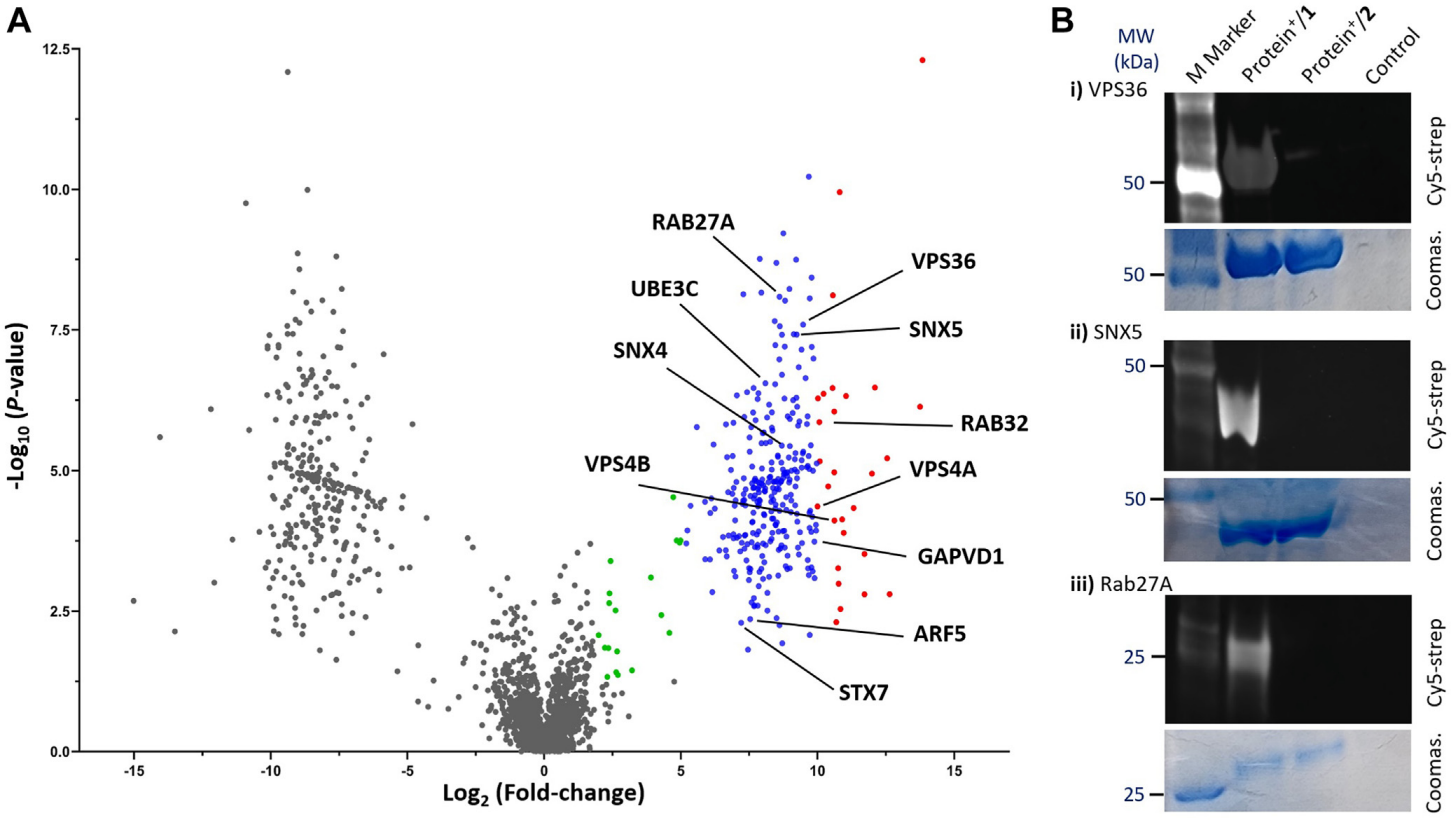
A: Volcano plot showing the distribution of proteins identified with **1**, presented in log_2_FC and log_10_ P, using proteins pulled down by **2** as controls. Color dots indicate significantly (*P* < 0.05) enriched proteins by ≥10 (red), 5 (blue), and 2 (green) folds. Some GSL-related proteins reported in the literature are specially marked. B: Cy5-streptavidin blot (top of each panel) and corresponding Coomassie staining (bottom of each panel) images of anti-FLAG resin-isolated proteins from *i*) FLAG-VPS36, *ii*) FLAG-SNX5, and *iii*) FLAG-Rab27A overexpressing HEK293 cells incubated with **1** (protein^+^/**1**) and **2** (protein^+^/**2**) or from wild-type cells (control) after UV light-mediated protein crosslinking and then biotinylation via a click reaction, to validate the specific interaction between **1** and VPS36, SNX5, or Rab27A proteins. The whole gel images are presented in supplemental Fig. S16.

Our proteomic results reveal 2,584 proteins (supplemental Fig. S14) identified with 1 (2,272 proteins) and 2 (2,284 proteins). The volcano plot of proteins pulled down by **1**, using proteins pulled down by 2 as references, is depicted in Fig. 5A. Among the 2,584 proteins, 1,972 are common for **1** and **2**, and 312 are unique for **1**. Of the 312 unique proteins (supplemental Table S2), 238 were observed in all three experiments, while the other 74 proteins were observed in any two experiments (supplemental Fig. S15). In addition to the unique proteins, we have also identified 65 proteins significantly enriched with **1** (supplemental Table S3). In the meantime, we have identified 300 proteins specific for **2** (i.e., not crosslinking to 1), of which 105 were observed in all three experiments (supplemental Table S4). Overall, many proteins are crosslinked with probes **1** and **2**. Nonetheless, each probe can selectively interact with a specific set of proteins, even though the two probes have the same lipid moiety. These results suggest the significant influence of the glycan structure of GSLs on their interaction with the cell membrane. The unique and highly enriched proteins identified with **1** and **2** should be useful targets for further in-depth investigation of GSLs as well as their signaling and metabolic pathways.

The identities of the 238 unique proteins pulled down by **1**, along with their cellular locations and potential biological functions, were analyzed in detail (supplemental Table S1). According to the Uniprot data, most of these proteins are associated with membranes, either plasma or intracellular organelle membranes. As anticipated, **1** and **2** target not only the plasma membrane but also the ER, Golgi, and nuclear membranes, because glycolipids can be internalized by cells via varied mechanisms. Optimizing the time of cell incubation with probes may help gain certain selectivity, according to the results in supplemental Figs. S2–S13. Nevertheless, cell internalization does not affect **1** and **2** as useful probes to explore GSL-cell membrane interactions; instead, it can broaden the application scope of these probes, for example, for the characterization of proteins related to GSL biosynthesis, transport, recycling, and trafficking, which is one of our future research directions.

More importantly, a series of the unique proteins pulled down by **1** (Table 1) are reported or predicted to be related to GSLs, including GSL endocytosis, trafficking, and GSL-mediated signaling. For example, the vacuolar protein sorting-associated proteins (VPSs) are associated with the endosomal sorting complex required for transport (ESCRT) that plays a role in transporting lipids from the early endosome to Golgi via the multivesicular body pathway (MVP) (114, 115). Their extracellular binding to GSLs mediates their clustering on cells, which is sensed by proteins within the lipid rafts to trigger endocytosis in clathrin-dependent and independent manners(116). GSL trafficking from the plasma membrane to the Golgi network is assisted by a retromer protein complex, which contains different VPS and SNARE proteins of the Syntaxin family. For example, various VPS proteins (such as VPS4A, VPS4B, VPS18, VPS36, and GAPVD1) participate in the retromer complex-mediated sphingolipid translocation from the membrane to the endosome. Categorically, VPS4 and VPS4A are required for retromer complex formation, and VPS4B plays a role in ESCRT-III complex disassembly in an ATP-driven manner. VPS18 and VPS36 contain a ubiquitin-binding domain, regulating membrane trafficking of ubiquitinated proteins via ESCRT-II complex formation (117). VPS18 and VPS36 association with ubiquitinated molecules also supports the presence of ubiquitin ligases or related components (e.g., UBE3C, UCHL3, IRF2BPL, supplemental Table S1) as unique proteins pulled down by **1**. Syntaxin 7 (STX7), as well as other syntaxin family proteins such as STX5, STX6, and STX16, are engaged in protein or lipid trafficking from plasma membrane to early endosome or other endocytic organelles, thus STX7 is expected to be involved in cellular translocation of GSLs. Sorting nexin proteins 4 and 5 (SNX4 and SNX5) are membrane proteins from the nexin protein family, possessing lipid-binding domains. Other nexins like SNX1 and SNX2 are reported to be present in the retromer complex and required for efficient retrograde transport of GSLs from early endosome to trans-Golgi network. Although there is no report directly linking SNX4 and SNX5 to GSLs, we anticipate that they are also involved in regulating GSL trafficking, because they share a similar sequence as SNX1 and SNX2. Ras-related proteins Rab-27A and Rab-32 are small GTPases, which act as binary on/off switches to control signal transduction. Other Rab proteins (such as Rab-7 and Rab-9) are known to be important mediators of the Golgi transport and trafficking of caveolae-internalized GSLs (87). Therefore, Rab-27A and Rab-32 are expected to participate in GSL trafficking as well. PALS2 is a protein localized at the lateral membrane of HEK293 cells as a component of mLin-7 complex, which regulates GSL and GPI endocytosis (106).

**Table 1:** GSL-related unique proteins identified with probe **1**

Proteins	Uniprot ID	Literature Reported Functions and Associations with GSLs	Ref.
Q9UN37	VPS4A	Vacuolar protein sorting (VPS) associated proteins, which are known to promote early endosome to Golgi transport of cellular proteins and lipids. GSL-binding Shiga toxins (STXs) requires retromer complex (SNX1 and SNX2, VPS) in their translocation process from cell membrane to trans-Golgi network.	(79, 80, 81, 82, 83)
O75351	VPS4B		
Q9P253	VPS18		
Q86VN1	VPS36		
Q14C86	GAPVD1		
O95219 Q9Y5X3	SNX4 SNX5	Sorting nexin proteins, which possess lipid binding domains. Various SNX proteins along with VPS proteins are key components of the mammalian retromer complex, which regulates GSL transport from endosome to trans-Golgi network.	(82, 83, 84, 85, 86)
P51159 Q13637	RAB27A RAB32	Rab proteins, which constitute the largest family of small GTPases that regulate cellular protein and lipid transport along the different stages of endocytic pathway. Rabs play an important role in GSL transport from late endosome to trans-Golgi network.	(87, 88, 89, 90, 91, 92, 93)
Q8N6T3	ARFGAP1	A GTP-ase activating protein for ADP-ribosylation factor 1, which mediates COPI vesicle formation from Golgi membrane. Binding of ARFGAP1 to the GSL fatty acid chain initiates the membrane bending, which acts as a starting point for different signaling pathway.	(94, 95, 96)
Q68EM7	ARHGAP17	A Rho-GTPase activating protein, which participates in Ca2+-dependent regulation of endocytosis and exocytosis of membrane proteins and glycolipids.	(97, 98, 99, 100)
P84085	ARF5	An ADP-ribosylation factor GTPase, which regulates vesicle trafficking. ARF5 regulates the biosynthesis of clathrin-independent endocytosis (CLIC) at the plasma membrane, required for GSL assembly with other signaling molecules.	(101, 102, 103)
Q15386	UBE3C	A ubiquitin-protein ligase, which interacts with a particular GSL called N5, to regulate the endocytosis of different notch receptors present on the cell surface. Ubiquitin-protein ligases act as mediators for the interaction between notch receptor and GSLs.	(104, 105)
Q9NZW5	PALS2	A scaffolding protein localized at the lateral membrane of kidney cells. As a component of mLin-7 complex, it interacts with GSLs or GPI anchors to regulate the endocytosis of transmembrane proteins.	(106, 107)
O15400	STX7	A protein from the membrane integrated SNARE protein family. It interacts with GPI-anchored proteins and GSLs to mediate protein trafficking to early endosome.	(108, 109, 110)
P84095	RHOG	A Rho-related GTP-binding protein, which interacts with glycolipid transfer proteins to regulate GSL distribution in different intracellular membranes.	(111, 112)
Q13823	GNL2	A nucleolar GTP-binding protein, which acts as GTPase involved in GSL biosynthesis.	(113)

We also analyzed the unique proteins identified with **2** and attempted to associate them with GSL-related processes but failed to find obvious correlations. This may be due to the unique structure of **2** with a GlcNAc residue directly linked to Cer. This linkage form has not been discovered in mammalian GSLs. Therefore, **2** may not have specific binding targets in the cell.

**Fig. 6:**
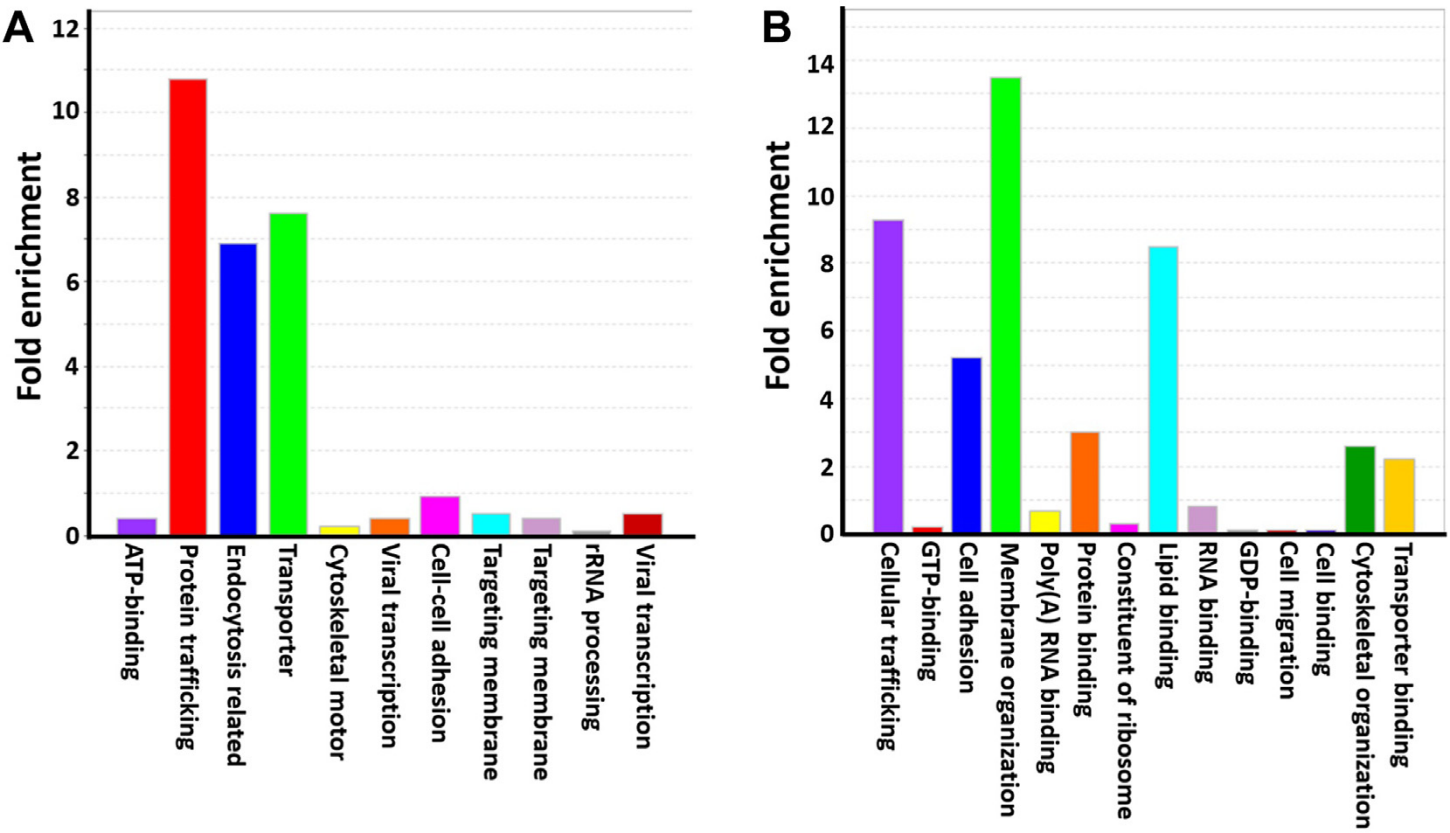
Gene ontology analysis of the 312 unique proteins pulled down by **1** with respect to (A) biological processes and (B) biological functions. The Y axis shows the folds of enrichment of specific proteins pulled down by probe **1**.

To further profile the unique proteins identified with **1**, we conducted bioinformatics studies. Gene ontology analysis of the 238 proteins observed in all three experiments indicates that the highly enriched proteins are related to protein and lipid trafficking, endocytosis, and transport (Fig. 6A). This is not surprising because the first step for GSL participation in various signaling pathways starts with extracellular ligand binding and then endocytosis and redistribution to ER, Golgi, and endosomes. Our analysis of the cellular functions of the unique proteins (Fig. 6B) results in similar conclusions. Functional analysis further reveals proteins that are associated with cytoskeletal rearrangement, cell adhesion, and ATP binding, which is also expected since GSLs are believed to initiate signaling processes via conspicuous cytoskeletal rearrangements (118, 119, 120). The remaining proteins are associated with RNA processing and RNA splicing, which is likely caused by probe incorporation into the cytoplasm and nucleus membranes.

### Validation of the proteomic results

To verify the proteomic results and prove that the interactions of **1** with the unique proteins pulled down by 1 were specific, we selected three of these proteins, VPS36, SNX5, and Rab27A, as models to perform further in-depth investigation. VPS36 was reported to assist ESCRT in regulating the retrograde transport of multivesicular endosomal (MVE) cargos (121). However, there is no report of direct interactions between VPS36 and GSLs, although GSLs have been found to play a critical role in MVE formation (122, 123, 124). SNX5 is mainly involved in intracellular protein trafficking via the retromer complex by facilitating cargo retrieval from the endosome to the trans-Golgi network. Although SNX proteins often possess GSL-binding domains (82, 83, 84, 85, 86), there is no report of GSL binding to SNX5 yet. Rab27A is a membrane-bound protein as an essential component of the melanosome receptor potentially involved in protein, GSL, and small GTPase-mediated signal transduction (125). Here, we aimed to validate that the observed interactions between 1 and VPS36, SNX5, or Rab27A were specific.

In these experiments, first, we overexpressed the target proteins carrying a DYKDDDDK (FLAG) tag using HEK293 cells and commercially available plasmids by established protocols. Next, the cells were incubated with 1 and 2 to label proteins interacting with them, following the procedure described above. Subsequently, the labeled proteins were extracted and applied to the click reaction to introduce a biotin tag. Finally, the target proteins were isolated using anti-FLAG resins and subjected to SDS-PAGE analysis by streptavidin-Cy5 blot. If the target protein is labeled with **1** or **2**, it should exhibit Cy5 fluorescence signals; otherwise, the target protein is not labeled with the probe. Our results from protein-overexpressing cells (Fig. 5B and supplemental Fig. S16) clearly indicate that VPS36, SNX5, and Rab27A were labeled by **1** but not by **2**, although cells treated with **1** and **2** expressed the same levels of FLAG-tagged VPS36, SNX5, and Rab27A proteins (see Coomassie blue staining results presented in Fig. 5B and supplemental Fig. S16). These results prove that VPS36, SNX5, and Rab27A crosslinked with **1** specifically, which is in accordance with our proteomic results. Considering that **1** and **2** contain the same lipid moiety and are different only in the glycans, these results are especially interesting, as they demonstrate the great influence of glycans of GSLs on their interaction with cell membranes and biomolecules on cells.

## Discussion

To facilitate the investigation of GSL-interacting membrane proteins, we have designed and synthesized bifunctional GSL probes **1** and **2**, which contain photoreactive diazirine and clickable alkyne. This design enables the cross-coupling of probes **1** and **2** with target membrane proteins upon UV irradiation and the labeling of crosslinked proteins with an affinity tag by click reaction to facilitate their isolation and identification, respectively. The design to have the diazirine and alkyne moieties separately located in the lipid and glycan can enhance the specificity of the probes because even if they are metabolically incorporated by cells into other GSLs and biomolecules, the two functional groups are unlikely to be present in the same biosynthetic products to act as bifunctional probes. We anticipate this probe design, as well as the relevant protocols, to be widely applicable to various GSLs. Eventually, we employed different techniques to validate that both probes **1** and **2** were effectively incorporated into the plasma and intracellular organelle membranes and pulled down specific proteins.

Using **1**, we have identified a series of unique proteins, many of which were previously reported as being related to GSL biosynthesis, trafficking, recycling, cytoskeletal rearrangement, and signaling. Triplicate experiments gave similar results, suggesting the reliability and reproducibility of the method. In addition, we have conducted experiments to further verify that VPS36, SNX5, and Rab27A, which are among the unique proteins pulled down by **1**, indeed interact with **1** specifically. Direct interactions of VPS36, SNX5, and Rab27A with GSLs have not been reported previously. Therefore, we have demonstrated that **1** and **2** are useful probes for studying GSL-membrane interaction and revealing new GSL-binding proteins. Moreover, although the embryonic kidney cell line HEK293 was used for the current work, the method and protocols described here should also be useful for studying other cell lines.

Another discovery of this research is that **1** and **2**, which have the same lipid moiety but different glycans, crosslink to different sets of membrane proteins. This directly proves the decisive influence of the glycan structure of GSLs on their properties or behaviors on the cell surface and their interaction with cellular membranes. It will also be interesting to study if probe 1 and the similar bifunctional derivative of LcGg4, which are epimers differing only in the stereochemistry of one carbon atom (Figs. 1 and 2), bind to the same membrane proteins or not. We further anticipate that the lipid tail of GSLs can also significantly affect the properties of GSLs and their interaction with membranes, which is another topic of our future research. This problem can be studied using similar GSL probes with different lipid moieties. While disclosing GSL-binding proteins in the plasma membrane is the key to understanding the functional roles and mechanisms of GSLs, identifying GSL-interacting proteins in intracellular membranes is also important, especially for in-depth investigation of the processes related to GSL biosynthesis, metabolism, cellular trafficking, and recycling. In this regard, the proteomic dataset generated herein is useful, although more accurate results can be obtained by modifying the experimental conditions, such as the duration of cellular treatment with the probe, because this can affect the probe distribution within cells. Therefore, the GSL probes developed here can be employed to study these problems as well. Finally, another future direction of this project is to study the identified proteins using techniques like genetic engineering to reveal their functions in GSL-regulated biological and pathological processes.

## Data availability

The datasets presented in this study can be found in Supplemental data of this paper and online repositories. The names of the repositories and the accession number(s) are as follows. The proteomic raw data and search results have been deposited to the ProteomeXchange Consortium via the PRIDE partner repository with the data set identifier PXD030528.

## Supplemental data

This article contains supplemental data (89, 106, 113, 126, 127, 128, 129, 130, 131, 132, 133, 134, 135, 136, 137, 138, 139, 140, 141, 142, 143, 144, 145, 146, 147, 148, 149, 150, 151, 152, 153, 154, 155, 156, 157, 158, 159, 160, 161, 162, 163, 164, 165, 166, 167, 168, 169, 170, 171, 172, 173, 174, 175, 176, 177, 178, 179, 180, 181, 182, 183, 184, 185, 186, 187, 188, 189, 190, 191, 192, 193, 194, 195, 196, 197, 198, 199, 200, 201, 202, 203, 204, 205, 206, 207, 208, 209, 210, 211, 212, 213, 214, 215, 216, 217, 218, 219, 220, 221, 222, 223, 224, 225, 226, 227, 228, 229, 230, 231, 232, 233, 234, 235, 236, 237, 238, 239, 240, 241, 242, 243, 244, 245, 246, 247, 248, 249, 250, 251, 252, 253, 254, 255, 256, 257, 258, 259, 260, 261, 262, 263, 264, 265, 266, 267, 268, 269, 270, 271, 272, 273, 274, 275, 276, 277, 278, 279, 280, 281, 282, 283, 284, 285, 286, 287, 288, 289, 290, 291, 292, 293, 294, 295, 296, 297, 298, 299, 300, 301, 302, 303, 304, 305, 306, 307, 308, 309, 310, 311, 312, 313, 314, 315, 316, 317, 318, 319, 320, 321, 322, 323, 324, 325, 326, 327, 328, 329, 330, 331, 332, 333, 334, 335, 336, 337, 338, 339, 340, 341, 342, 343, 344, 345, 346, 347, 348, 349).

## Conflict of interest

The authors declare no conflict of interest.
