# Supplementary material for: Bifunctional glycosphingolipid (GSL) probes to investigate GSL-interacting proteins in cell membranes
Source: J Lipid Res. 2024 May 23;65(7):100570. doi: 10.1016/j.jlr.2024.100570 (PMC11261293; doi:10.1016/j.jlr.2024.100570)
Supplement: Supplemental data [file mmc1.pdf]

Supporting Information

Bifunctional Glycosphingolipid (GSL) Probes to Investigate GSL-Interacting Proteins in Cell Membranes

Sayan Kundu,[1‡] Rajendra Rohokale,[1‡] Chuwei Lin,[2] Sixue Chen,[2,3] Shayak Biswas,[1] and Zhongwu Guo[1*]

[1]*Department of Chemistry, University of Florida, Gainesville, FL 32611, USA*

[2]*Department of Biology, Genetics Institute, University of Florida, Gainesville, FL 32611, USA*

[3]*Department of Biology, University of Mississippi, Oxford, MS 38677, USA*

[‡]These authors contributed equally to the current work.

*Corresponding author email: zguo@chem.ufl.edu

Table of Contents

I. Additional Results of Biological Studies (Figure S1-S16 and Tables S1-S4)……………..….SI-2

II. NMR and MS Spectra of All New Compounds (Figures S17-S41)..………………………SI-44

I. Additional Results of Biological Studies

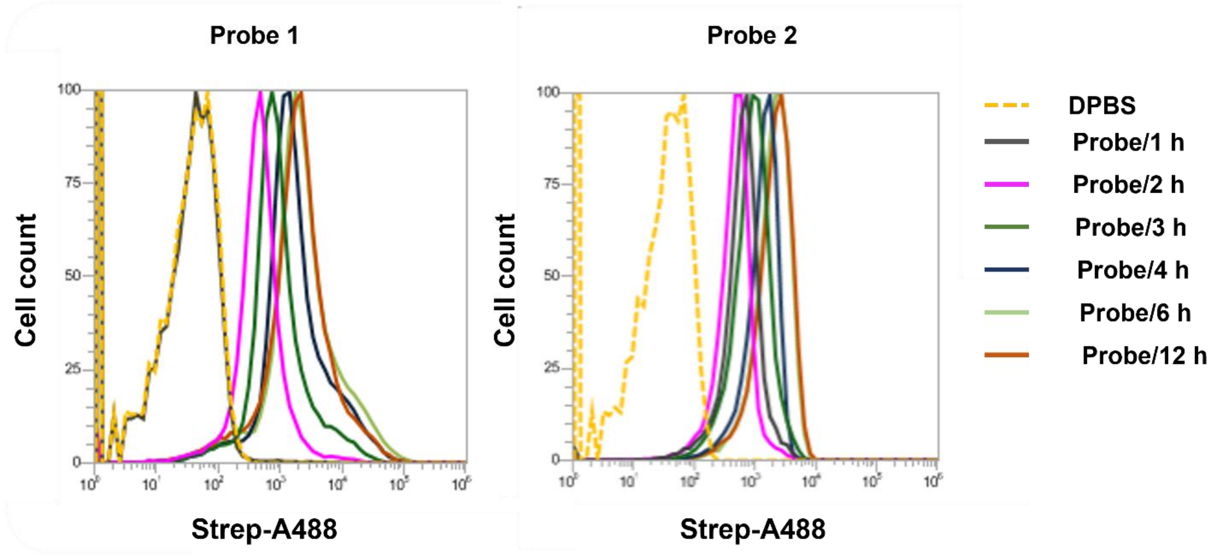

Figure S1. FACS results showing the incorporation of probes **1** and **2** by HEK293 cells. After the cells were treated with **1** or **2** (200 μM) for 1, 2, 3, 4, 6, and 12 h, they were successively incubated with biotin-azide (100 μM) for 45 min and A488-streptavidin (1:500 dilution) for 30 min, which was followed by FACS analysis using an Attune NXT flow cytometer.

Table S1: List of antibodies used to stain organelles for the study of organelle localization of **1** and **2**.

Target organelle	Antibody used	Provider: Catalogue number
Endoplasmic reticulum (ER)	Alexa fluorophore® 647 Anti-Calreticulin [EPR3924]	Abcam: ab196159
Golgi	Alexa fluorophore® 647 Anti-GM130 [EP892Y]	Abcam: ab195303
Early endosome	Alexa fluorophore® 647 Anti-EEA1 [EPR4245]	Abcam: ab196186
Late endosome	Alexa fluorophore® 647 Anti-RAB7 [EPR7588(B)]	Abcam: ab310133
Lysosome	Alexa fluorophore® 647 Anti-LAMP1 [EPR21026]	Abcam: ab237303

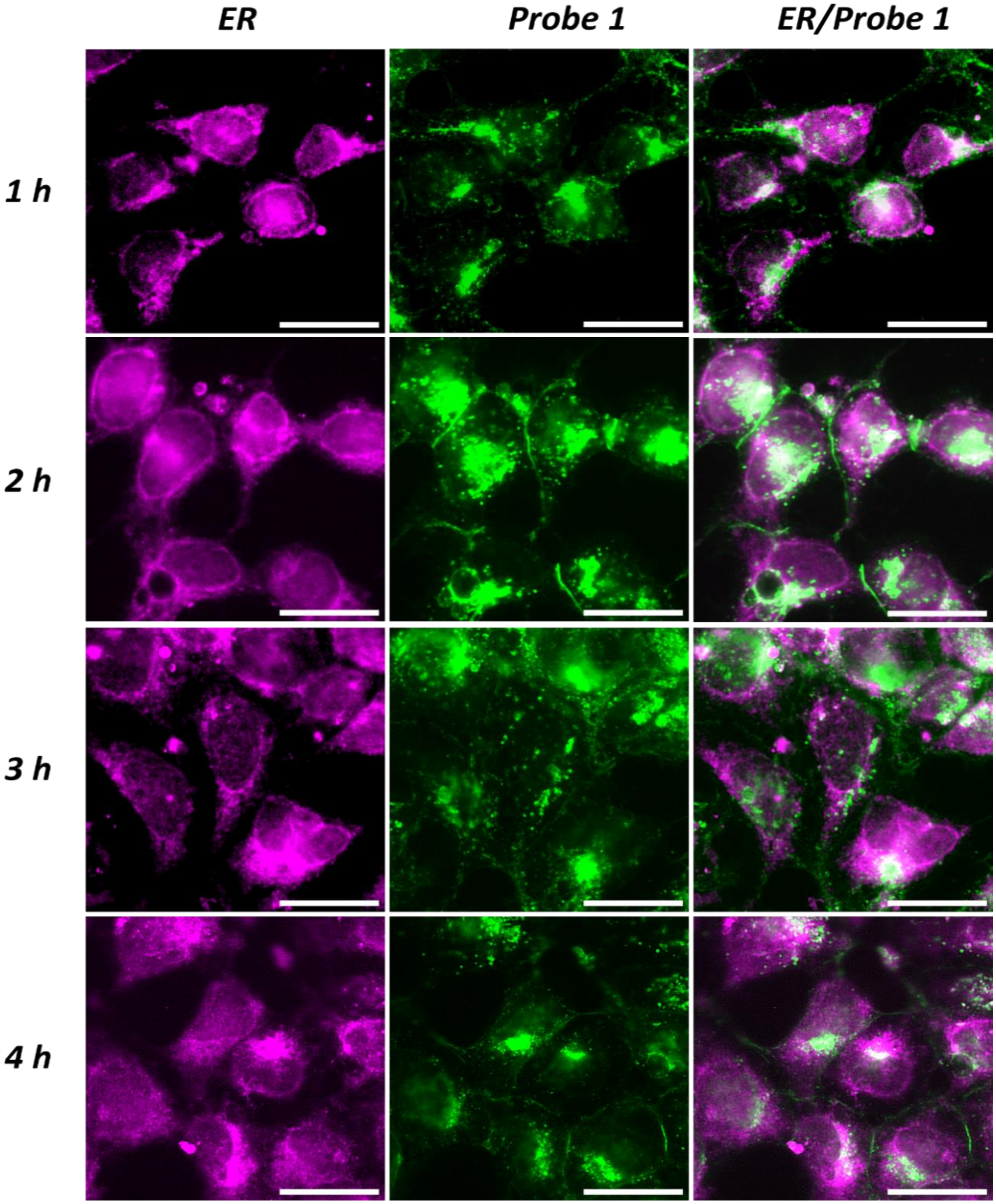

Figure S2: Fluorescent images of HEK293 cells showing the time-dependent changes in the co-localization of **1** and ER. After HEK293 cells were incubated with **1** (50 µM) for 1, 2, 3 or 4 h and then biotin-azide and streptavidin-A488 (green) to label **1**, these cells were fixed, permeabilized, and finally treated with A647 (purple)-anti Calreticulin antibody to stain ER, which was followed by fluorescence imaging. The scale bars are 20 µm.

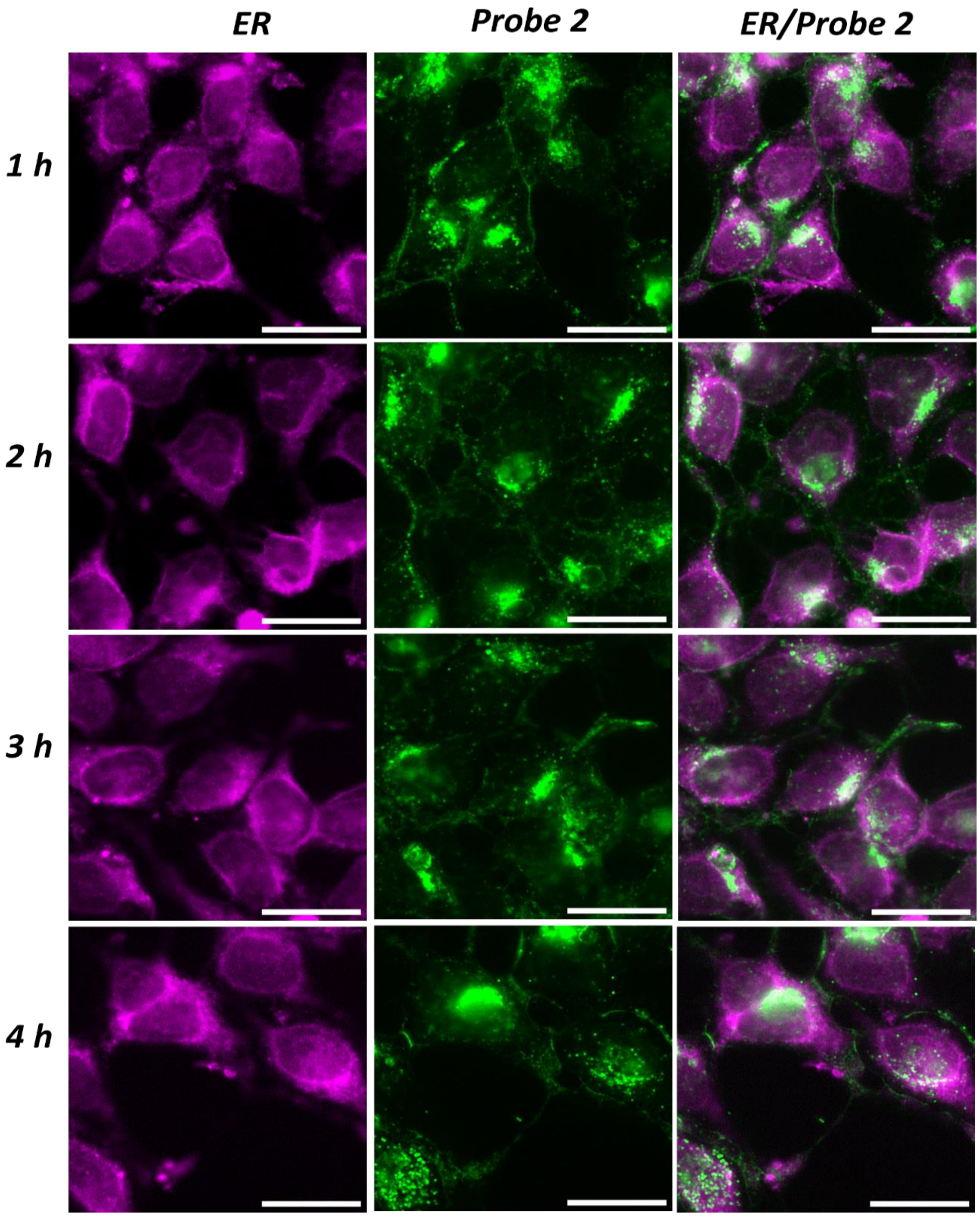

Figure S3: Fluorescent images of HEK293 cells showing the time-dependent changes in the co-localization of **2** and ER. After HEK293 cells were incubated with **2** (50 μM) for 1, 2, 3 or 4 h and then biotin-azide and streptavidin-A488 (green) to label **2**, these cells were fixed, permeabilized, and finally treated with A647 (purple)-anti Calreticulin antibody to stain ER, which was followed by fluorescence imaging. The scale bars are 20 μm.

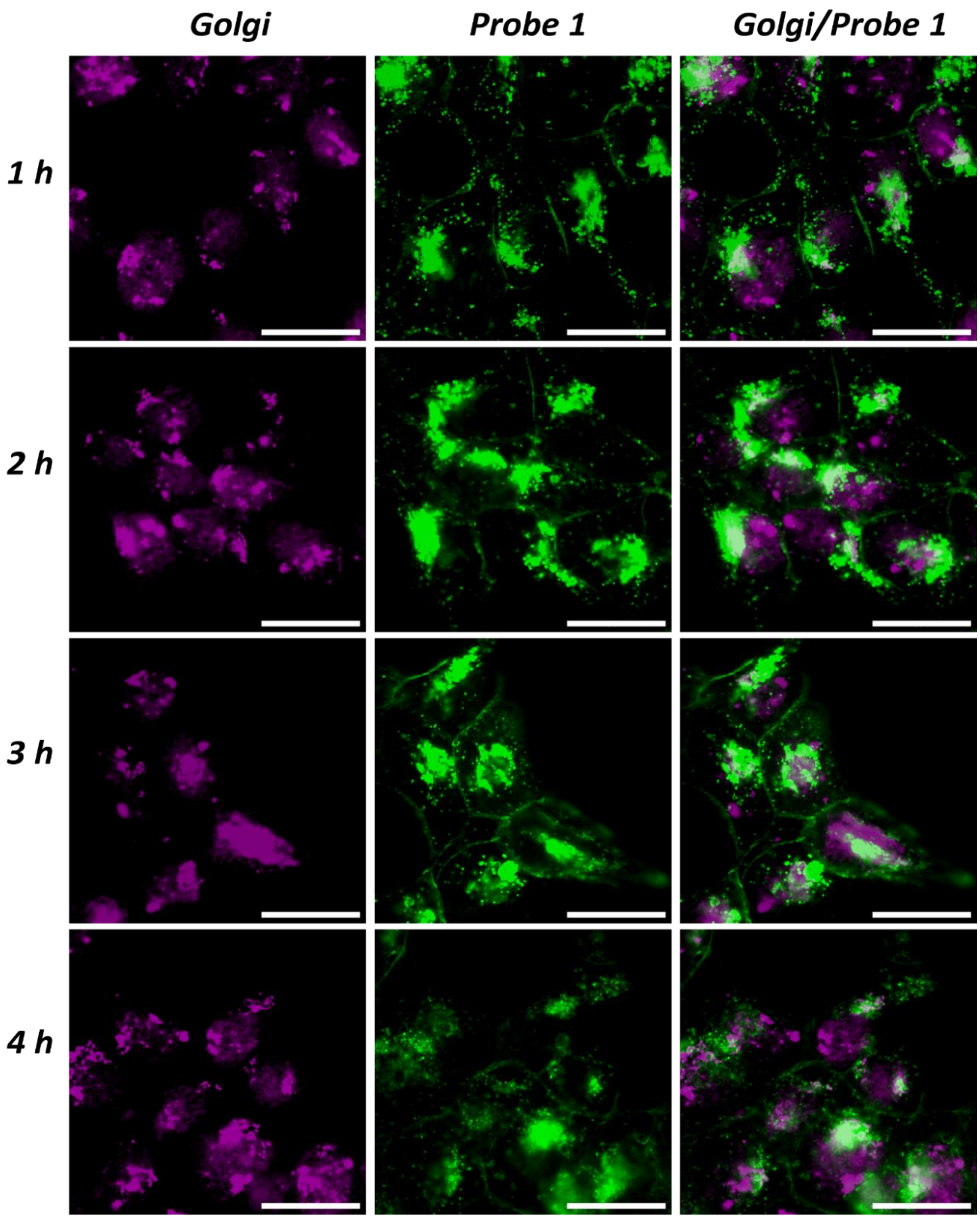

Figure S4: Fluorescent images of HEK293 cells showing the time-dependent changes in the co-localization of **1** and Golgi. After HEK293 cells were treated with **1** (50 μM) for 1, 2, 3 or 4 h and then biotin-azide and streptavidin-A488 (green) to label **1**, these cells were fixed, permeabilized, and finally treated with A647 (purple)-anti GM130 antibody to stain Golgi, which was followed by fluorescence imaging. The scale bars are 20 μm.

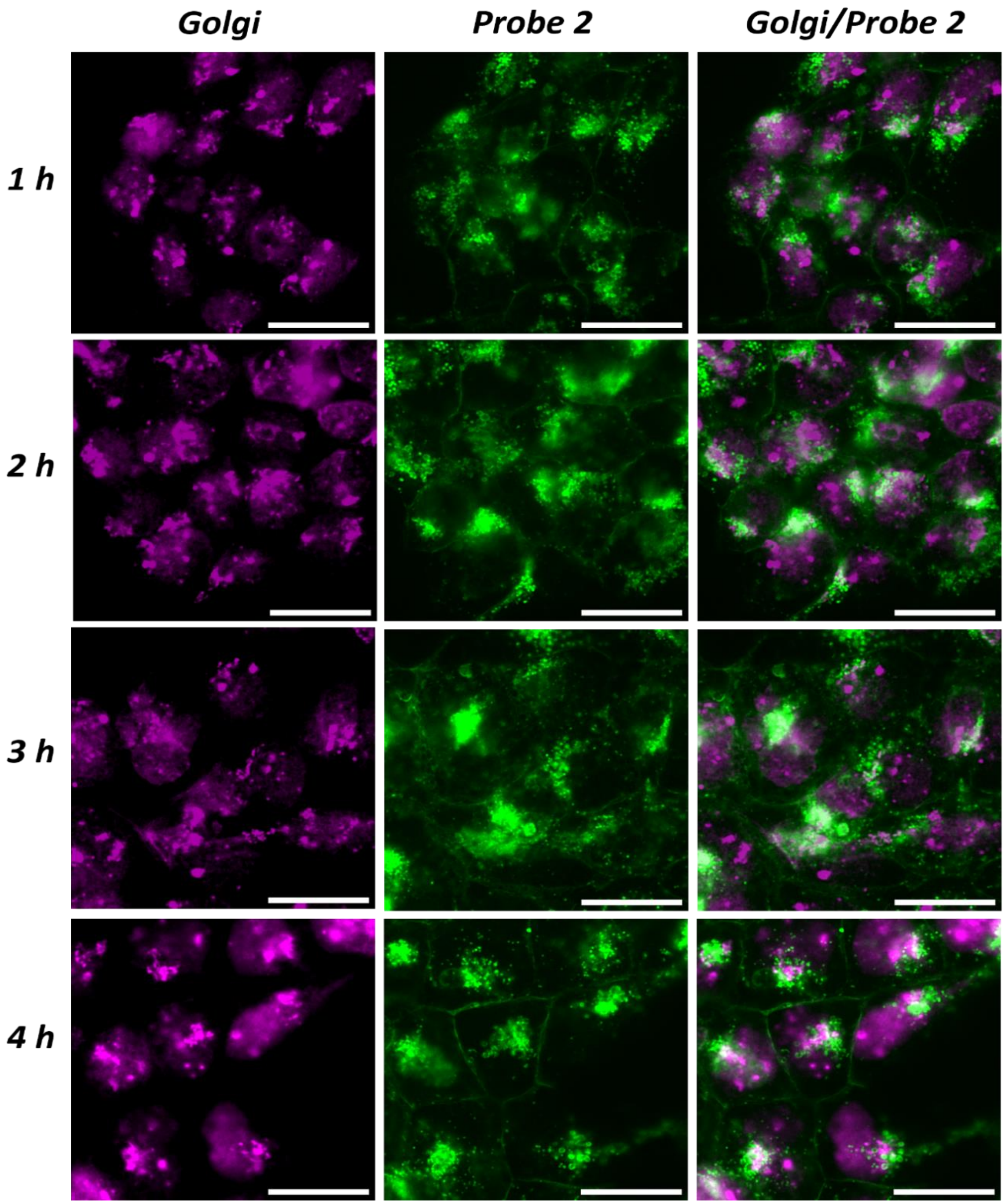

Figure S5: Fluorescent images of HEK293 cells showing the time-dependent changes in the co-localization of **2** and Golgi. After HEK293 cells were treated with **2** (50 μM) for 1, 2, 3 or 4 h and then biotin-azide and streptavidin-A488 (green) to label **2**, these cells were fixed, permeabilized, and finally treated with A647 (purple)-anti GM130 antibody to stain Golgi, which was followed by fluorescence imaging. The scale bars are 20 μm.

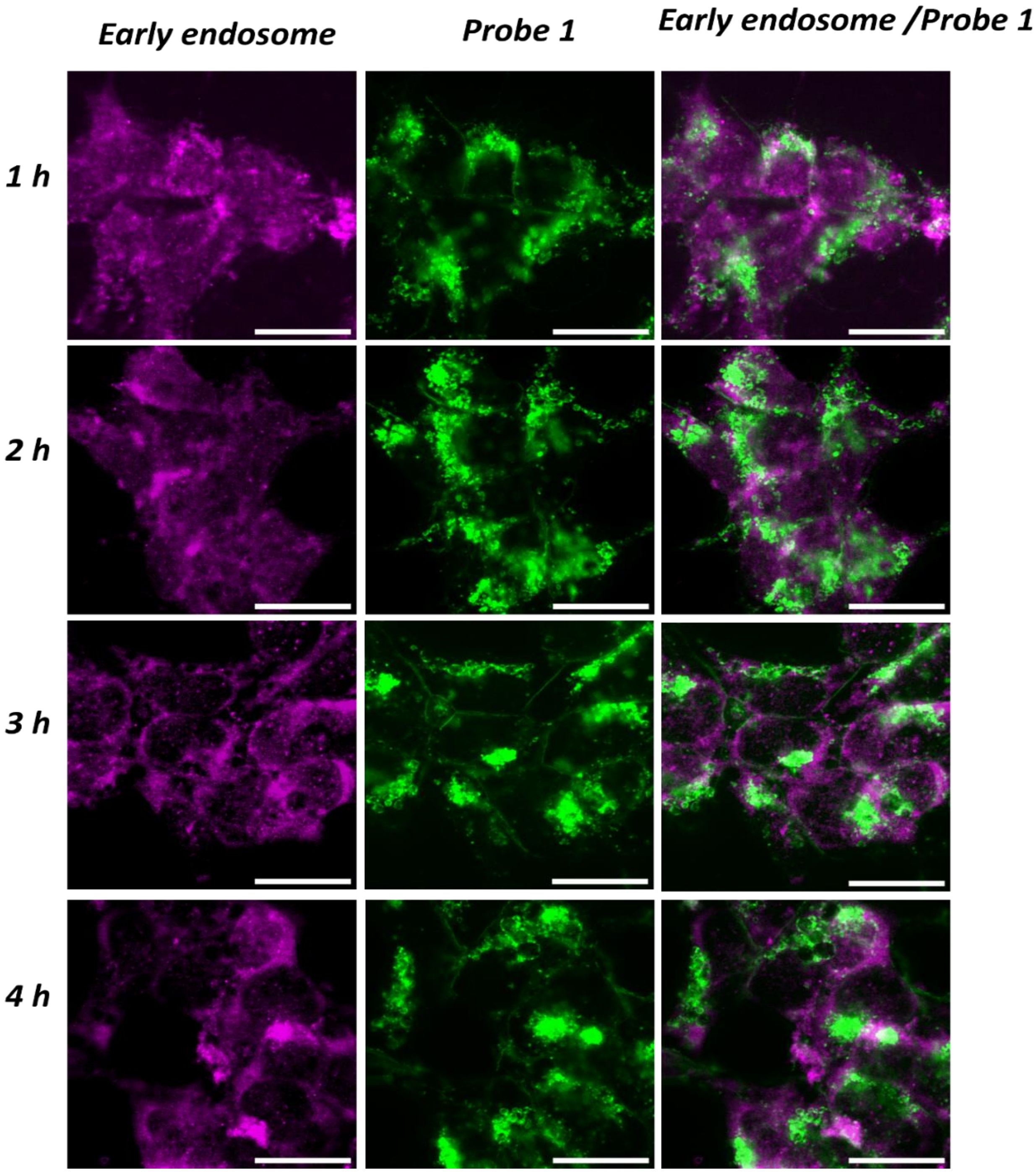

Figure S6: Fluorescent images of HEK293 cells showing the time-dependent changes in the co-localization of **1** and early endosome. After HEK293 cells were treated with **1** (50 µM) for 1, 2, 3 or 4 h and then with biotin-azide and streptavidin-A488 (green) to label **1**, these cells were fixed, permeabilized, and finally treated with A647 (purple)-anti EEA1 antibody to stain early endosome, which was followed by fluorescence imaging. The scale bars are 20 µm.

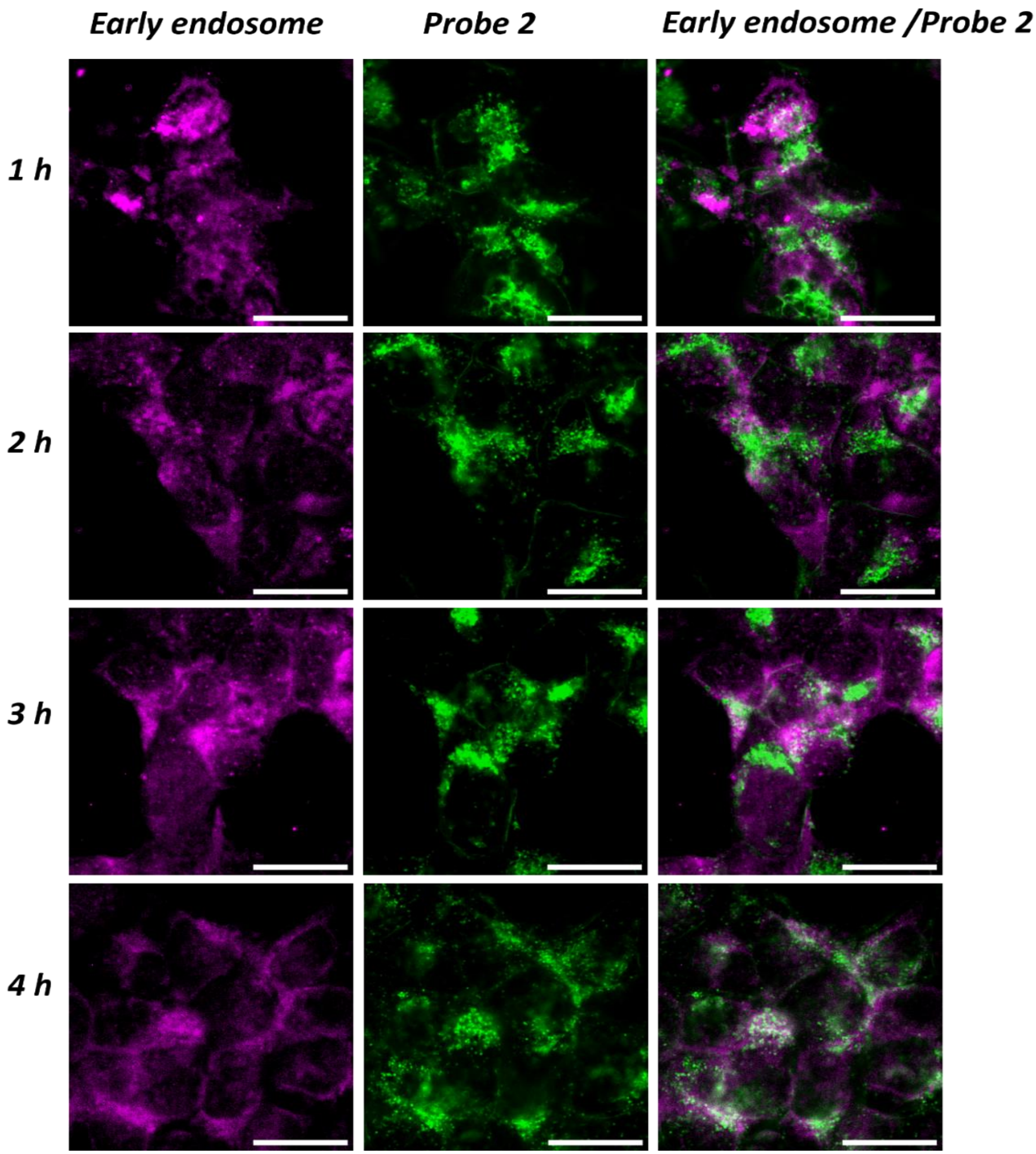

Figure S7: Fluorescent images of HEK293 cells showing the time-dependent changes in the co-localization of **2** and early endosome. After HEK293 cells were treated with **2** (50 μM) for 1, 2, 3 or 4 h and then with biotin-azide and streptavidin-A488 (green) to label **2**, these cells were fixed, permeabilized, and finally treated with A647 (purple)-anti EEA1 antibody to stain early endosome, which was followed by fluorescence imaging. The scale bars are 20 μm.

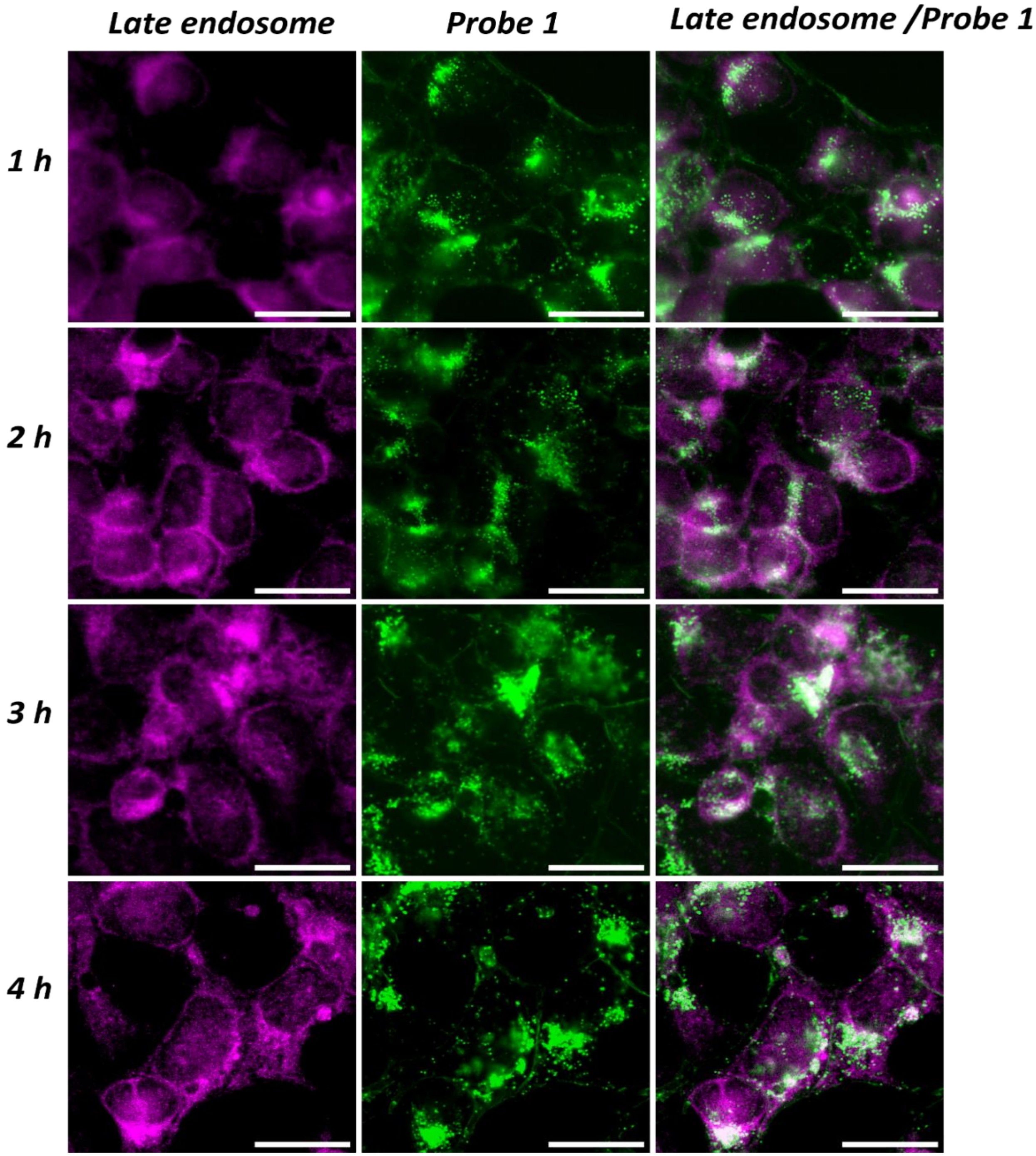

Figure S8: Fluorescent images of HEK293 cells showing the time-dependent changes in the co-localization of **1** and late endosome. After HEK293 cells were treated with **1** (50 µM) for 1, 2, 3 or 4 h and then with biotin-azide and streptavidin-A488 (green) to label **1**, these cells were fixed, permeabilized, and finally treated with A647 (purple)-anti RAB7 antibody to stain late endosome, which was followed by fluorescence imaging. The scale bars are 20 µm.

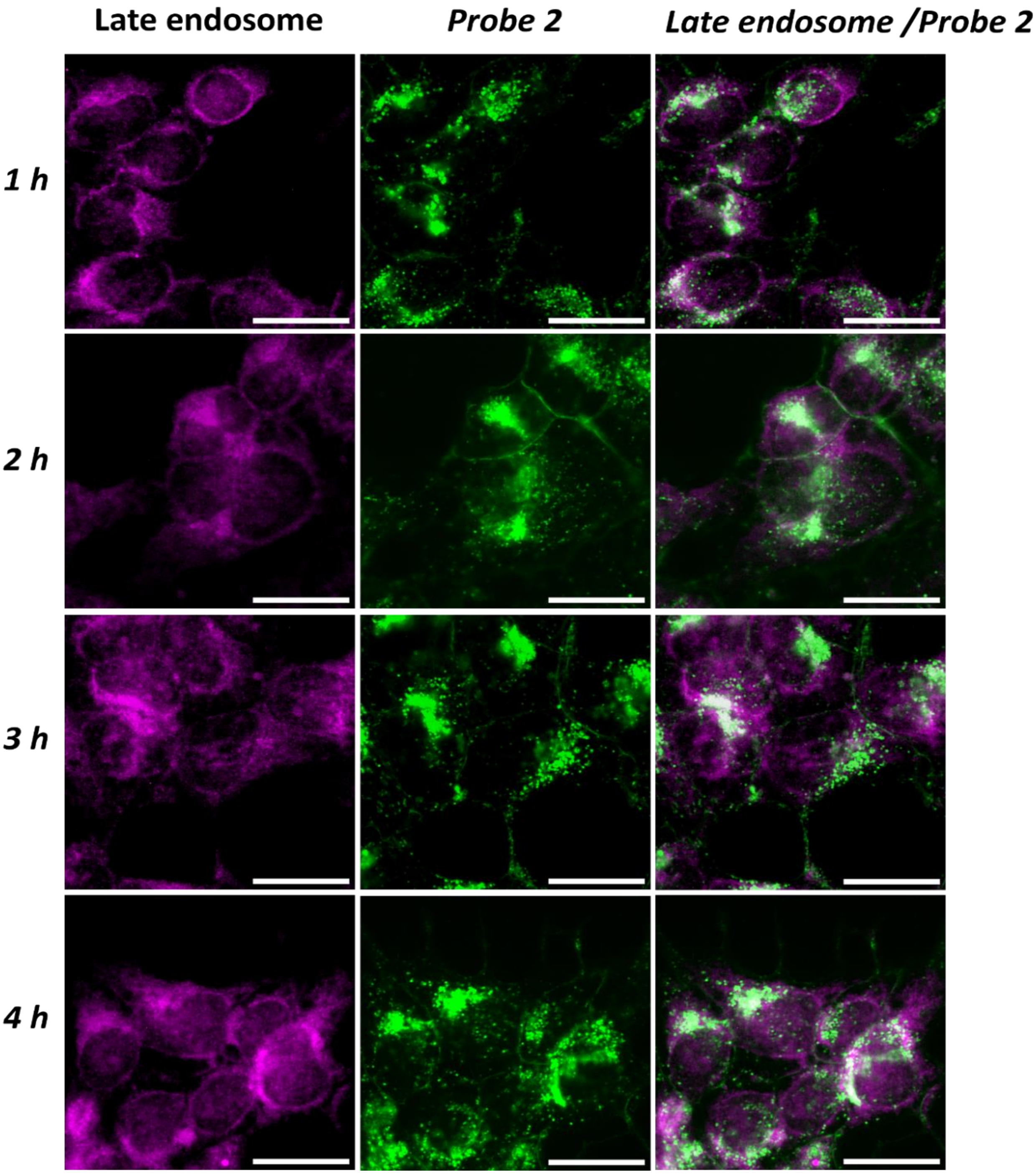

Figure S9: Fluorescent images of HEK293 cells showing the time-dependent changes in the co-localization of **2** and late endosome. After HEK293 cells were treated with **2** (50 μM) for 1, 2, 3 or 4 h and then with biotin-azide and streptavidin-A488 (green) to label **2**, these cells were fixed, permeabilized, and finally treated with A647 (purple)-anti RAB7 antibody to stain late endosome, which was followed by fluorescence imaging. The scale bars are 20 μm.

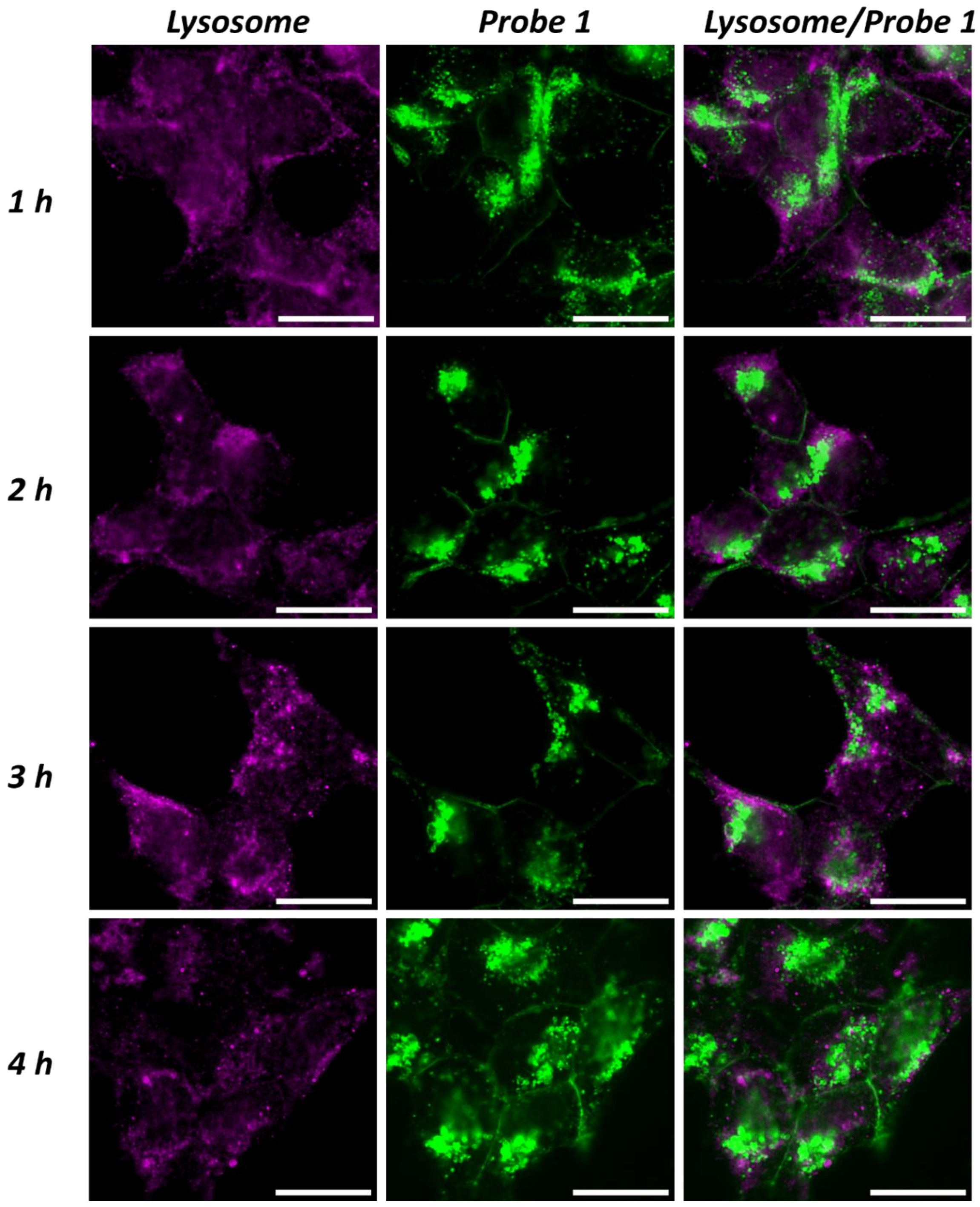

Figure S10: Fluorescent images of HEK293 cells showing the time-dependent changes in the co-localization of **1** and lysosome. After HEK293 cells were treated with **1** (50 µM) for 1, 2, 3 or 4 h and then biotin-azide and streptavidin-A488 (green) to label **1**, the cells were fixed, permeabilized, and finally treated with A647 (purple)-anti LAMP1 antibody to stain lysosome, which was followed by fluorescence imaging. The scale bars are 20 µm.

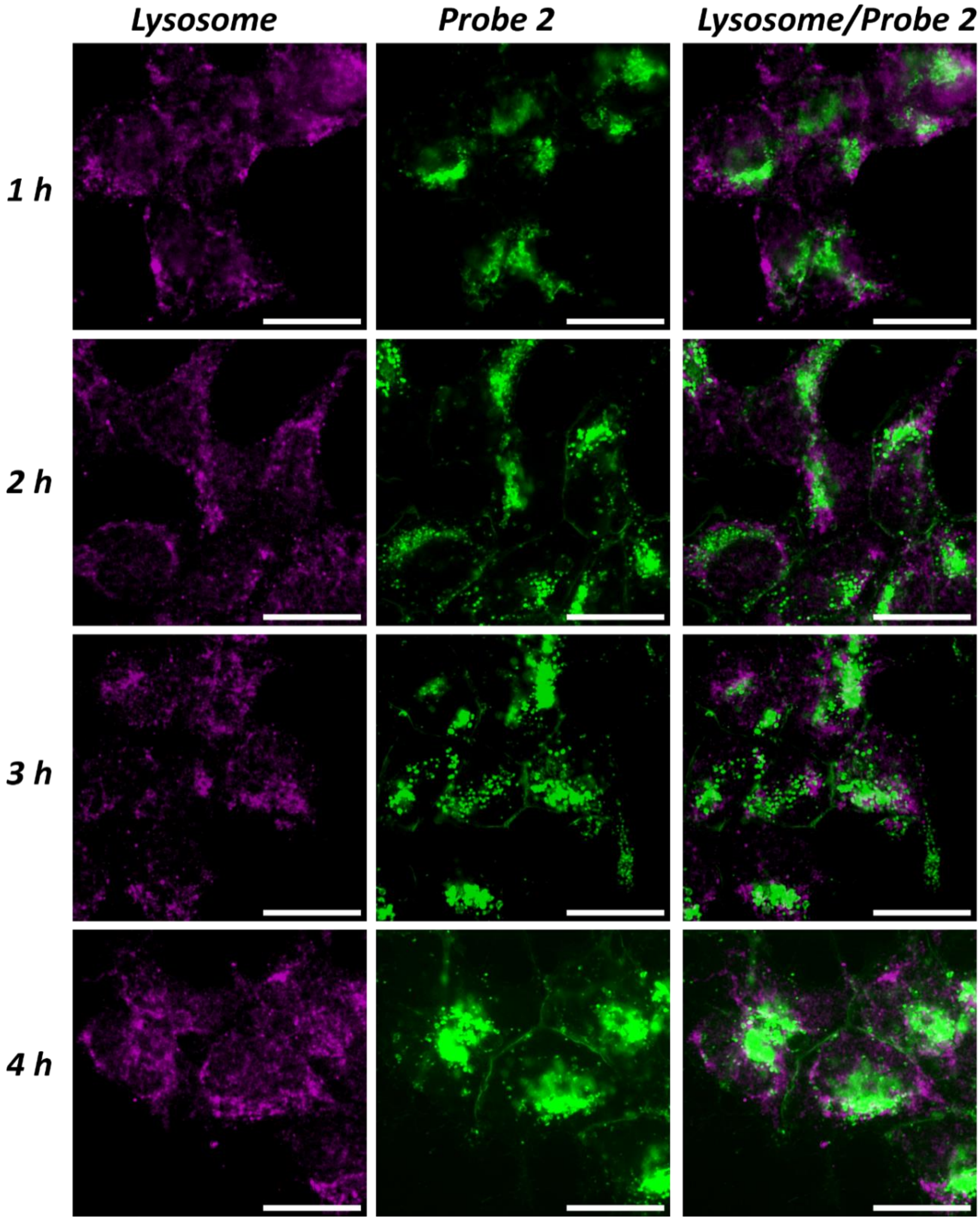

Figure S11: Fluorescent images of HEK293 cells showing the time-dependent changes in the co-localization of **2** and lysosome. After HEK293 cells were treated with **2** (50 μM) for 1, 2, 3 or 4 h and then biotin-azide and streptavidin-A488 (green) to label **2**, the cells were fixed, permeabilized, and finally treated with A647 (purple)-anti LAMP1 antibody to stain lysosome, which was followed by fluorescence imaging. The scale bars are 20 μm.

a)

b)

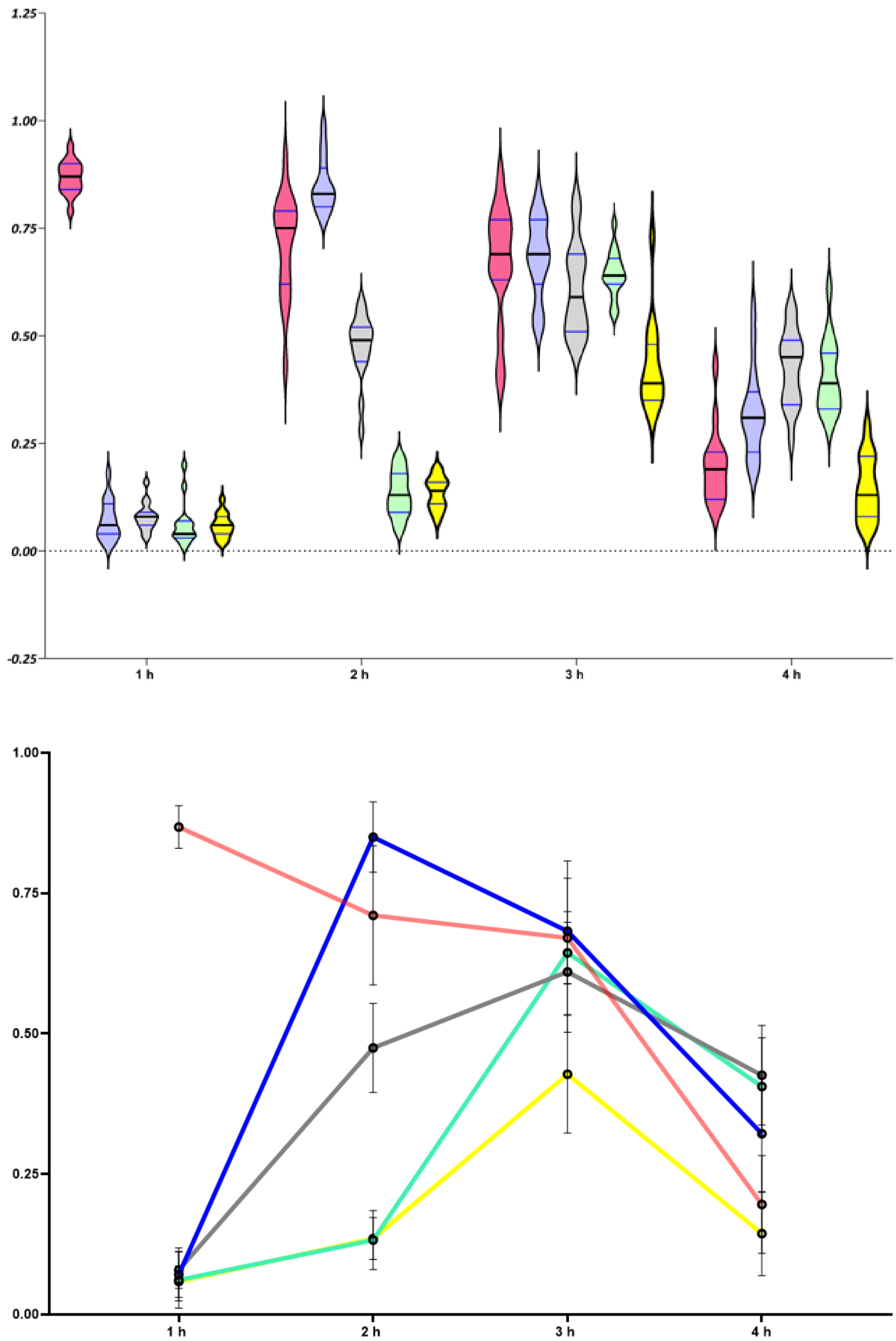

Figure S12: a) Violin and **b**) line plots to show the trends of Pearson correlation coefficients for 15 stained HEK293 cells from five independent fluorescent micrographs, demonstrating the co-localization of the fluorescence of **1** with that of ER (red), Golgi (blue), early endosome (grey), late endosome (green), and lysosome (yellow) at different duration (1, 2, 3, and 4 h; shown in X-axis) of incubation with probe **1**.

a)

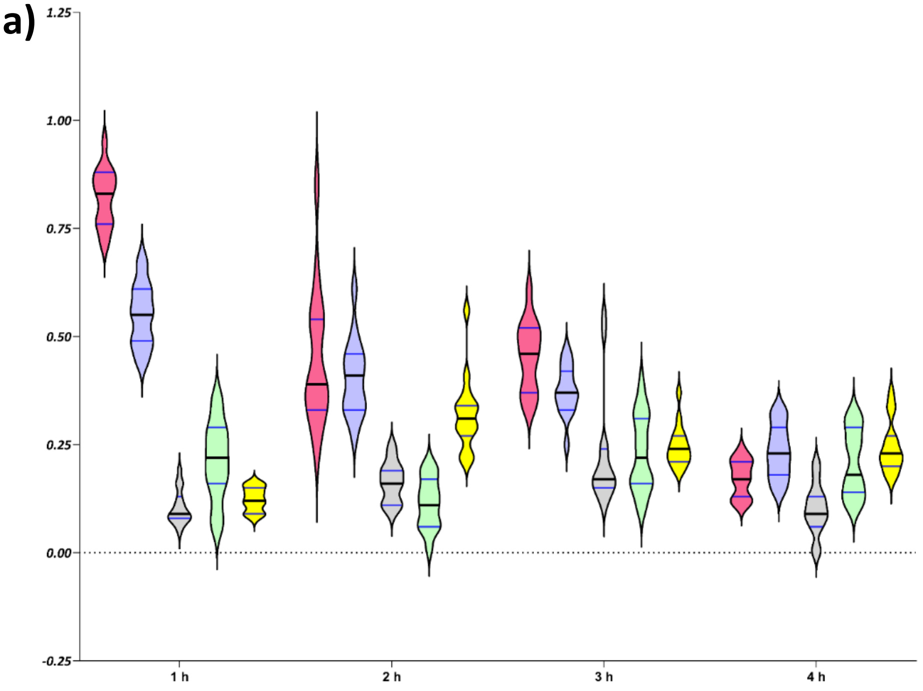

b)

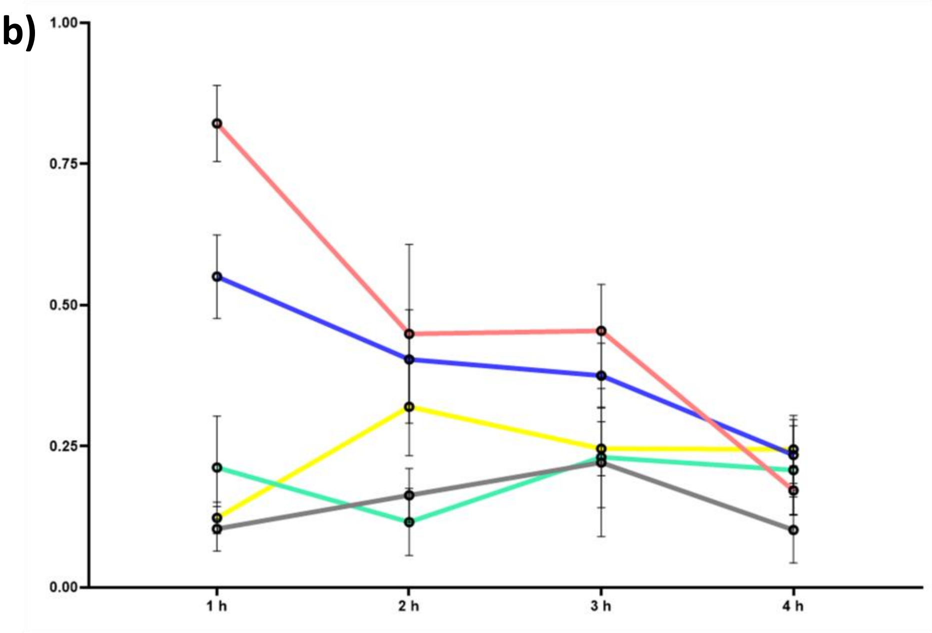

Figure S13: a) Violin and **b**) line plots to show the trends of Pearson correlation coefficients for 15 stained HEK293 cells from five independent fluorescent micrographs, demonstrating the co-localization of the fluorescence of **2** with that of ER (red), Golgi (blue), early endosome (grey), late endosome (green), and lysosome (yellow) at different duration (1, 2, 3, and 4 h; shown in X-axis) of incubation with probe **2**.

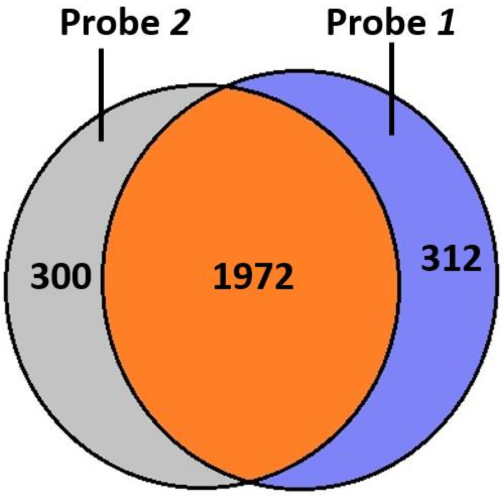

Figure S14. Total numbers of proteins pulled down by probes **1** and **2**, respective, as well as the numbers of overlapped and unique proteins among all proteins identified.

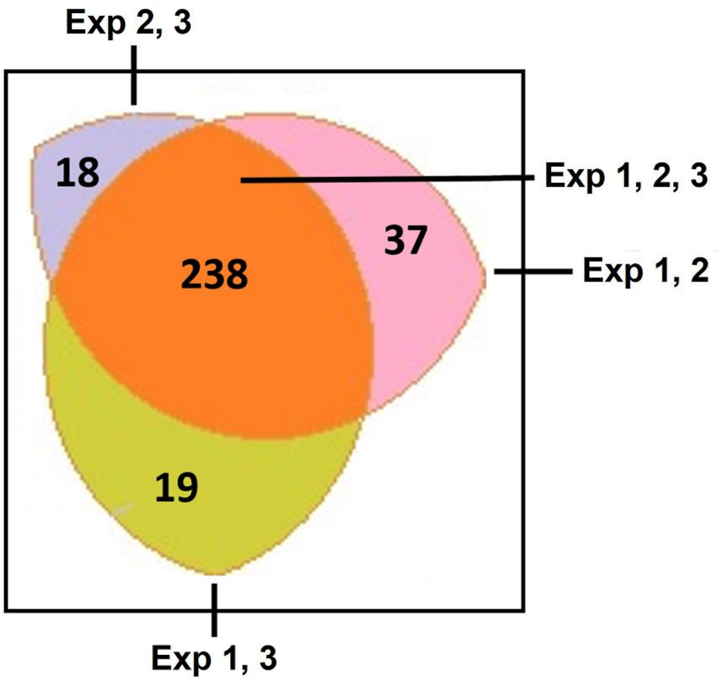

Figure S15. Schematic representation of the unique proteins identified with probe **1** as observed in three independent experiments. Among these proteins, 238 (shown in orange) were discovered in each of the three experiments.

Table S2. Unique proteins pulled down by probe **1** from HEK293 cells in all three experiments.

Proteins	Genes	Cellular locations	Log_2 (FC)	Protein names and reported or predicted biological functions	Ref
**Q8IX12**	CCAR1	Cytoplasm	13.85	Cell division cycle and apoptosis regulator protein 1: It acts as a coactivator protein recruited by nuclear receptors and thus plays a role in activating the transcription process for many cell surface proteins.	[1, 2]
**Q13765**	NACA	Cytoplasm	13.75	Nascent polypeptide-associated complex subunit alpha: It plays a role in preventing inappropriate targeting of the non-secretory peptide to the ER.	[2-4]
**Q9BVC6**	TMEM109	Nuclear membrane, ER membrane	12.55	Transmembrane protein 109: It is an ER membrane protein that mediates cellular response against DNA damage. It is believed to play a role in voltage-gated ion channel activity.	[2]
**Q6NZI2**	CAVIN1	Cell membrane	12.1	Caveolae-associated protein 1: A cell membrane protein, which plays an important role in caveolae formation and organization. Caveolae formation depends on its interaction with PtdIns in the lipid raft.	[2, 5]
**P04899**	GNAI2	Cytoskeleton, Cell membrane	12	Guanine nucleotide-binding protein G(i) subunit alpha-2: G proteins are involved in the different transmembrane signaling pathways.	[2, 6]
**Q9UN37**	VPS4A	Late endosome membrane, Cytoplasm	11.32	Vacuolar protein sorting-associated protein 4A: A protein involved in the later step of the endosomal pathway for cellular trafficking of membrane proteins and lipids. It is closely associated with lipid droplets, indicating their roles in lipid metabolism.	2, 7
**O60869**	EDF1	Cytoplasm, Nucleus	11.05	Endothelial differential-related factor 1: It is a transcriptional coactivator protein playing a role in lipid metabolism.	[2, 8]
**P84095**	RHOG	Cell membrane	10.96	Rho-related GTP-binding protein RhoG: Rho family GTPases play a role in pathway related to cytoskeletal rearrangement and activate Cdc42 and Rac1 proteins.	[2, 9]
**P00918**	CA2	Cytoplasm, Cell membrane	10.91	Carbonic anhydrase 2: A protein that facilitates cellular uptake of carbon dioxide by catalyzing the hydration of CO_2.	[2]
**P62891**	RPL39	Cytoplasm	10.84	60S ribosomal protein L39: Subunit of ribonucleoprotein complex required for protein synthesis in the cells and thus participates in cell proliferation.	[2, 10]

**O15126**	SCAMP1	Clathrin-coated vesicle, Plasma membrane	10.82	Secretory carrier-associated membrane protein (SCAMP) 1: Integral membrane protein involved in membrane protein trafficking.	[2, 11]
**P49773**	HINT1	Cytoplasm, cytoskeleton	10.78	Adenosine 5'-mono phosphor amidase HINT1: It hydrolyzes purine nucleotide phosphoramidates and thus participates in cell signaling.	[2]
**Q8WZ42**	TTN	Cytosol, Extracellular exosome	10.76	Titin: It plays a role in structural stability of cellular structure.	[2]
**P20810**	CAST	Cytoplasm, Endoplasmic reticulum	10.69	Calpastatin: It acts as an inhibitor of calcium-dependent cysteine protease.	[2, 12]
**Q9Y5X3**	SNX5	Early endosome, cell membrane	10.62	Sorting nexin-5: Involved in different steps of intracellular trafficking and interacting with phosphatidylinositol phosphates.	[2, 13]
**Q9HC35**	EML4	Cytoplasm, Cytoskeleton, microtubule	10.62	Echinoderm microtubule-associated protein-like 4: Involved in the function and stability of microtubules. It plays a role in formation of cytoplasmic droplets and thus initiates many signaling pathway.	[2, 14]
**Q86U42**	PABPN1	Nucleus, Cytoplasm	10.61	Polyadenylate-binding protein 2: Involved in RNA binding and RNA processing.	[2]
**Q9BYD6**	MRPL1	Mitochondria	10.56	39S ribosomal protein L1, mitochondrial: It helps protein synthesis in mitochondria.	[2]
**P13995**	MTHFD2	Mitochondrial matrix	10.56	Bifunctional methylenetetrahydrofolate dehydrogenase: A folate-coupled enzyme, which has NAD specific hydrolase activity.	[2, 15]
**Q14914**	PTGR1	Cytoplasm	10.39	Prostaglandin reductase 1: A NAD(P)H dependent oxidoreductase, responsible for fatty acid and lipid metabolism.	[2]
**P63000**	RAC1	Cell membrane	10.23	Ras-related C3 botulinum toxin substrate 1: A plasma membrane-associated Rho family small GTPase protein that cycles between GTP and GDP and thus initiates different signaling pathway.	[2, 16]
**Q16740**	CLPP	Mitochondrial matrix	10.1	ATP-dependent Clp protease proteolytic subunit: A component of mitochondrial Clp protease complex, which plays a role in protein quality control.	[2, 17]
**Q96S44**	TP53RK	Cytoplasm	10.07	EKC/KEOPS complex subunit TP53RKL: A typical protein kinase that phosphorylates serine residue of P53 oncogenes.	[2]

**P15374**	UCHL3		10.02	Ubiquitin carboxyl-terminal hydrolase isozyme L3: A protein playing a role in controlling the level of cellular ubiquitin through processing of ubiquitin precursors.	[2]
**Q02127**	DHODH	Cytoplasm	10	Dihydroorotate dehydrogenase: A mitochondrial membrane protein playing a major role in electron transport chain in mitochondria.	[2, 18]
**Q14432**	PDE3A	Plasma membrane	9.95	cGMP-inhibited 3',5'-cyclic phosphodiesterase 3A: A phosphodiesterase that hydrolyzes cyclic GMP (important secondary messenger) and thus participates in different signaling pathways. Different phosphodiesterases are reported to interact with caveolae (sphingolipid enriched lipid raft).	[2, 19, 20]
**P52943**	CRIP2	Cell cortex	9.95	Cysteine-rich protein 2: An adaptor protein playing a role in metal ion binding activity and regulates cell proliferation.	[2, 21]
**O60343**	TBC1D4	Cytoplasm	9.90	TBC1 domain family member 4: It acts as an activator of Rab family GTPase proteins. TBC domain containing proteins participate in cell signaling by interacting with ARF family of G proteins.	[2, 22]
**Q14C86**	GAPVD1	Plasma membrane, Endosome	9.89	GTPase-activating protein and VPS9 domain-containing protein 1: A guanine nucleotide exchange factor for small GTPases, responsible for protein endocytosis of Ras related proteins.	[2, 23]
**P46976**	GYG1	Cytoplasm	9.85	Glyogenin-1: An enzyme involved in the biosynthesis of glycogen.	[2]
**Q9Y371**	SH3GLB1	Cytoplasm	9.83	Endophilin-B1: An endophilin class of protein playing a role in clathrin-independent endocytosis of membrane proteins.	[2, 24]
**O60925**	PFDN1	Cytoplasm	9.83	Prefoldin subunit 1: A protein regulating the correct folding status of the cytosolic proteins.	[2, 25]
**P29144**	TPP2	Cytoplasm	9.82	Tripeptidyl-peptidase 2: A serine protease enzyme, which removes tripeptide from amino terminus and thus activates different signaling pathway.	[2, 26]
**P27144**	AK4	Mitochondrial matrix	9.79	Adenylate kinase 4: A protein to maintain the homeostasis of nucleotides, thus playing a role in regulating cellular ATP levels.	[2, 27]
**P62070**	RRAS2	Plasma membrane	9.78	Ras-related protein R-Ras2: A Ras family of GTPase protein with GTP binding activity, to control multiple signaling pathway.	[2, 28]
**O75351**	VPS4B	Endosome membrane	9.76	Vacuolar protein sorting-associated protein 4B: A protein involved in the later step of endosomal pathway, and participating in	[2, 29]

				membrane associated ESCRT-II complex mediated cargo transport.	
**P49005**	POLD2	Nucleus	9.74	DNA polymerase delta subunit 2: A protein involved in DNA polymerase activity.	[2]
**Q15428**	SF3A2	Nucleus	9.72	Splicing factor 3A subunit 2: Involved in mRNA slicing activity.	[2]
**Q9BTE6**	AARSD1	Nucleus	9.72	Alanyl-tRNA editing protein Aarsd1: It plays a role in the correction of incorrectly placed tRNA.	[2]
**Q8WVC0**	LEO1	Nucleus	9.71	RNA polymerase-associated protein LEO1: It plays a role in regulation of transcription and thus is involved in different signaling pathway.	[2, 30]
**Q96CX2**	KCTD12	Cell membrane	9.68	BTB/POZ domain-containing protein KCTD12: A potassium channel receptor that promotes cell cycle by regulating the PI3K-AKT signaling pathway.	[2, 31]
**Q6Y7W6**	GIGYF2	ER membrane, Cytoplasm	9.68	GRB10-interacting GYF protein 2: A component protein of multiprotein complex regulating initiation of translation.	[2]
**Q9Y6V0**	PCLO	Cytoskeleton	9.68	Protein piccolo: A scaffold protein playing a role in vesicle clustering, membrane fusion, and endocytosis.	[2, 32]
**Q9Y4P3**	TBL2	ER membrane	9.66	Transducin beta-like protein 2: A protein in the ER membrane regulating cell survival.	[2]
**P26885**	FKBP2	ER	9.64	Peptidyl-prolyl cis-trans isomerase FKBP2: A PPIase protein playing a role in proper folding of proteins.	[2]
**Q8IXM2**	BAP18	Nucleus	9.64	Chromatin complexes subunit BAP18: A component of chromatin complex, playing a role in DNA modification.	[2, 33]
**Q9HCG8**	CWC22	Cytoplasm	9.62	Pre-mRNA-splicing factor CWC22 homolog: Involved in mRNA splicing, and thus regulating spliceosome complex.	[2, 34]
**Q9BYD1**	MRPL13	Mitochondrial inner membrane	9.56	39S ribosomal protein L13: A protein involved in protein synthesis in mitochondria, and thus participating in PI3K-AKT pathway.	[2, 35]
**P55957**	BID	Cytoplasm, Mitochondrial membrane	9.48	BH3-interacting domain death agonist: It plays a crucial role in receptor-mediated apoptosis in many cells.	[2, 36]
**P49589**	CARS1	Cytoplasm	9.46	Cysteine tRNA ligase: It plays a role in ATP-dependent ligation of cysteine to tRNA.	[2]
**P49770**	EIF2B2	Cytoplasm	9.43	Translation initiation factor eIF-2B subunit beta: It acts as GEF for eukaryotic initiation factor 2, and thus regulates different signaling pathways.	[2]

**P13647**	KRT5	Cytoplasm	9.42	Keratin, type II cytoskeletal 5: A component of keratin protein, which constructs structural framework of the cells.	2
**Q9HBK9**	AS3MT	Cytoplasm	9.34	Arsenite methyltransferase: A protein that catalyzes the transfer of methyl group to form methyl arsonate.	2
**Q9BYG3**	NIFK	Cytoplasm, Nucleus	9.32	MKI67 FHA domain-interacting nucleolar phosphoprotein: An NIFK protein binding to Ki-7 protein to mediate kinase activities of different downstream proteins to regulate the cell cycle.	2, 37, 38
**Q8IWA0**	WDR75	Nucleus	9.32	WD repeat-containing protein 75: A ribosomal protein responsible for RNA processing.	2, 39
**Q9NPD3**	EXOSC4	Cytoplasm	9.30	Exosome complex component 4: A component protein of RNA exosome complex, which plays a role in RNA processing and degradation.	2, 40
**Q8TEA8**	DTD4	Nucleus, Cytoplasm	9.30	D-aminoacyl-tRNA deacylase 1: An ATPase protein involved in replication of DNA.	2
**Q9H1B7**	IRF2BPL	Nucleus	9.2	E3 ubiquitin-protein ligase IRF2BP: An E3 ubiquitin ligase, responsible for regulating targeted protein degradation. It also regulates Wnt/β-catenin signaling pathway.	2, 41
**Q9UGP8**	SEC63	Plasma membrane, ER	9.2	Translocation protein SEC63 homolog: SEC protein complex (SEC61/62/63) mediates post translational transport of certain polypeptide chains across ER.	2, 42
**Q6UW68**	TMEM205	Membrane	9.25	Transmembrane protein 205: A multi-pass transmembrane protein, playing an important role in progression of cancer cells.	2, 43
**Q9BPX5**	ARPC5L	Cytoskeleton	9.25	Actin-related protein 2/3 complex subunit 5-like protein: A component protein of Arp complexes, which are involved in actin polymerization.	2, 44
**P51159**	RAB27A	Plasma membrane	9.2	Ras-related protein Rab-27A: A small GTPase protein responsible for regulating late endocytic pathway. Different Rab GTPase proteins are responsible for sphingolipid endocytosis and trafficking.	2, 45, 46
**P36915**	GNL1	Nucleus	9.2	Guanine nucleotide-binding protein-like 1: It plays a role in selective internalization of proteins from the plasma membrane. GNL1 also plays a role in diverse function in vesicle coat formation as well as vesicle-cytoskeletal interaction.	2, 47, 48
**Q9NY12**	GAR1	Nucleus	9.22	H/ACA ribonucleoprotein complex subunit 1: A component of small nucleolar riboprotein	2

				complex, which catalyzes pseudouridylation of rRNA.	
**Q5TDH0**	DDI2	cytoplasm	9.20	Protein DDI1 homolog 2: A protein playing a crucial role in regulating proteasome activity towards the ubiquitinated substrate.	[2, 49]
**Q9NRR5**	UBQLN4	Cytoplasm, ER	9.19	Ubiquilin-4: A protein mediating targeted protein degradation by ubiquitinating the target proteins.	[2, 50]
**P61011**	SRP54	Cytoplasm, ER	9.18	Signal recognition particle 54 kDa protein: An important component of signal recognition particle complex involved in targeting of secretory and membrane proteins to the ER.	[2, 51]
**P04637**	TP53	cytoplasm	9.18	Cellular tumor antigen P53: It acts as an important tumor suppressor protein by regulating cell division.	[2, 52]
**O95989**	NUDT3	Cytoplasm, Nucleus	9.13	Diphosphoinositol polyphosphate phosphohydrolase 1: It is involved in the hydrolysis of different inositol phosphates, and thus regulate different signaling pathway.	[2, 53]
**Q6IAN0**	DHRS7B	ER membrane	9.12	Dehydrogenase/reductase SDR family member 7B: An NAD(P)(H) dependent reductase, which plays a crucial role in carbohydrate and lipid metabolism.	[2, 54]
**A6NDG6**	PGP	cytoplasm	9.09	Glycerol-3-phosphate phosphatase: It regulates the cellular level of glycerol-3-phosphate, and thus participates in lipid metabolism.	[2]
**Q9P2X0**	DPM3	ER membrane	9.05	Dolichol-phosphate mannosyltransferase subunit 3: A protein that stabilizes Dolichol-phosphate-mannose (DPM) synthase complex at the ER, and thus regulates post translational modification of proteins.	[2, 55]
**Q92879**	CELF1	Nucleus, Cytoplasm	9.03	CUGBP Elav-like family member 1: An RNA-binding protein, which regulates different post transcriptional events, e.g., mRNA splicing, mRNA translation etc.	[2, 56]
**P82970**	HMGN5	Nucleus	9.03	High mobility group nucleosome-binding domain-containing protein 5: A member of HMGN protein family. It affects transcription of different proteins and thus plays a role in cell proliferation and invasion.	[2, 57]
**O95302**	FKBP9	ER	8.99	Peptidyl-prolyl cis-trans isomerase FKBP9: A PPIase proteins, which plays a role in proper folding of protein during protein synthesis.	[2]
**Q5SY16**	NOL9	Nucleus	8.99	Polynucleotide 5'-hydroxyl-kinase NOL9: It plays a role in rRNA processing.	[2]
**Q99986**	VRK1	Cytoplasm	8.99	Serine/threonine-protein kinase VRK1: A serine/threonine kinase protein playing a role	[2]

				in cell cycle and many other different cellular pathway.	
**Q9Y4C8**	RBM19	Nucleus, Cytoplasm	8.98	Probable RNA-binding protein 19: A protein playing a role in RNA recognition and thus participating in ribosomal RNA processing.	[2, 58]
**Q13425**	SNTB2	Plasma membrane	8.96	Beta-2-syntrophin: Aan adaptor protein that binds and thus determines the cellular localization of membrane proteins. SNTB2 also acts as a linker protein for various membrane proteins with cytoskeleton.	[2, 59]
**O95563**	MPC2	Mitochondrial inner membrane	8.95	Mitochondrial pyruvate carrier 2: It mediates pyruvate uptake in mitochondria.	[2]
**P51452**	DUSP3	Nucleus	8.92	Dual specificity protein phosphatase 3: It shows phosphatase activity for tyrosine and serine protein phosphate and participates in different cellular signaling.	[2, 60]
**O94855**	SEC24D	Cytoplasmic vesicles	8.90	Protein transport protein Sec24D: A component of coat protein complex II (COPII), which plays a role in formation of transport vesicles from ER.	[2, 61]
**Q9GZT8**	NIF3L1	Cytoplasm	8.87	NIF3-like protein 1: It acts as transcriptional corepressor by interacting with COPS2, and thus regulates COP9 signaling pathway.	[2, 62]
**P18583**	SON	Nucleus	8.86	Protein SON: A nuclear protein with RNA binding domain; it participates in mRNA splicing.	[2, 63]
**Q14696**	MESD	ER, Plasma membrane	8.86	LRP chaperone MESD: A chaperone protein, which assists proper folding of cell surface receptor protein.	[2]
**Q7Z5L9**	IRF2BP2	Cytoplasm, Nucleus	8.84	Interferon regulatory factor 2-binding protein 2: It acts as transcriptional corepressor.	[2]
**Q92541**	RTF1	Nucleus	8.82	RNA polymerase-associated protein RTF1 homolog: A component of protein complex that associates with RNA polymerase.	[2]
**Q9UNQ2**	DIMT1	Nucleus	8.81	Probable dimethyladenosine transferase: A protein involved in rRNA processing.	[2]
**P32321**	DCTD	Cytoplasm	8.80	Deoxycytidylate deaminase: It regulates the deamination reaction of deoxy-cytidine monophosphate (dcMP) to deoxy-uridine monophosphate (dUMP).	[2, 64]
**Q9NQT8**	KIF13B	Cytoplasm, cytoskeleton	8.80	Kinesin-like protein KIF13B: Playing a role in organization of cytoskeleton, and thus involved in protein trafficking from plasma membrane to cytoplasm.	[2, 65]

**Q8WX93**	PALLD	Actin cytoskeleton, Actin filament	8.79	Palladin: A cytoskeleton protein playing a role in cytoskeleton organization and participating in cell-extracellular matrix organizations.	[2, 66]
**Q9H3S7**	PTPN23	Cytoplasm, Endosome	8.75	Tyrosine-protein phosphatase non-receptor type 23: It plays a role in sorting of endocytic cargo by interacting with endosomal sorting complex.	[2, 67]
**Q13685**	AAMP	Plasma membrane, Cytoplasm	8.74	Angio-associated migratory cell protein: Playing a role in cell migration by regulating RhoA pathway.	[2, 67]
**O00193**	SMAP	Plasma membrane, Cytoskeleton	8.73	Small acidic protein: An Arf-specific GTPase protein, which binds to clathrin heavy chains and thus is involved in protein trafficking.	[2, 68]
**Q96TC7**	RMDN3	Mitochondrial membrane, Cytoplasm	8.73	Regulator of microtubule dynamics protein 3: A protein showing microtubule binding and involved in calcium homeostasis and other cellular pathways.	[2, 69]
**O43665**	RGS10	Cytoplasm, Nucleus, membrane	8.72	Regulator of G-protein signaling 10: Regulating G protein-coupled receptor signaling pathway by increasing GTPase activity of G_α subunit.	[2, 70]
**P02461**	COL3A1	ER matrix	8.71	Collagen alpha 1(III) chain: One of the most abundant cellular proteins, which helps to maintain mechanical properties and organization of cellular cytoskeleton.	[2, 71]
**Q02818**	NUCB1	Golgi membrane	8.70	Nucleobindin-1: A Golgi resident protein that has a major role in calcium homeostasis. It also acts as non-receptor GEF to activate G proteins, and thus regulates various signaling pathways.	[2, 72]
**Q96EK5**	KIFBP	Cytoskeleton	8.70	KIF-binding protein: A kinesin binding protein involved in the organization of microtubules and thus membrane protein trafficking.	[2, 73]
**Q5T6F2**	UBAP2	Cytoplasm, Nucleus		Ubiquitin-associated protein 2: It regulates ubiquitination for RNA polymerase proteins.	[2, 74]
**Q9Y2Q9**	MRPS28	Mitochondrial inner membrane	8.68	28S ribosomal protein S28, mitochondrial: A component of mitochondrial ribosomal proteins, which plays a role in oxidative phosphorylation.	[2, 75]
**Q69YN2**	CWF19L1	Post-mRNA release splicosomal complex	8.65	CWF19-like protein 1: A protein having predicted role in cell cycle control and endosomal trafficking.	[2, 76]
**Q86VN1**	VPS36	Cytoplasm, Endosome, and late endosome	8.63	Vacuolar protein-sorting-associated protein 36: A component of endosomal sorting complex (ESCRT-II), which is required for endosomal cargo protein trafficking.	[2, 77]

**Q14789**	GOLGB1	Cytoplasm, ER-Golgi network	8.62	Golgin subfamily B member 1 or Giantin: Present in the Golgi matrix, participating in the membrane trafficking of the proteins.	[2, 78]
**Q8WVY7**	UBLCP1	Nucleus	8.61	Ubiquitin-like domain-containing CTD phosphatase 1: A proteasome phosphatase that regulates the nuclear proteasome activity.	[2, 79]
**Q96B26**	EXOSC8	Cytoplasm, Nucleus	8.61	Exosome complex component RRP43: A component of RNA-exosome complex, required for RNA degradation and processing.	[2, 80]
**Q9H2P9**	DPH5	Cytoplasm	8.61	Diphthine methyl ester synthase: It acts as methyltransferase for histidine residue of the target proteins.	[2]
**Q08AM6**	VAC14	Early endosome membrane	8.58	Protein VAC14 homolog: It acts as a scaffold protein component of phosphatidylinositol 3,5-biphosphate (PtdIns(3,5)P2). It also plays a role in the biogenesis of endosome carrier vesicles and thus transport of protein to the early endosome.	[2, 81]
**Q01658**	DR1	Nucleus	8.57	Protein Dr1: It plays a role in transcription related cellular process.	[2, 82]
**Q9NYH9**	UTP6	Nucleus	8.56	U3 small nucleolar RNA-associated protein 6 homologs: It is involved in Nuclear RNA processing.	[2]
**Q8WXA9**	SREK1	Nucleus	8.56	Splicing regulatory glutamine/lysine-rich protein 1: It plays a role in mRNA processing and splicing.	[2]
**P09417**	QDPR	Cytoplasm, exosome	8.55	Dihydropteridine reductase: It plays a role in electron transfer activity.	[2]
**O43148**	RNMT	Nucleus	8.52	mRNA cap guanine-N7 methyltransferase: It plays a role in RNA binding and methyl transferase activity to RNA.	[2]
**P43155**	CRAT	Cytoplasm, ER	8.52	Carnitine O-acetyltransferase: It plays a role in acyltransferase activity.	[2]
**P84085**	ARF5	Plasma membrane	8.51	ADP-ribosylation factor 5: A GTP binding protein, playing a role in protein trafficking from plasma membrane to Golgi.	[2, 22]
**Q9NW13**	RBM28	Nucleus	8.5	RNA-binding protein 28: A component of ribonucleoprotein complex, involved in mRNA processing and mRNA splicing.	[2, 83]
**Q8IX01**	SUGP2	Nucleus	8.5	SURP and G-patch domain-containing protein 2: It plays a role in mRNA splicing.	[2]
**Q9BUN8**	DERL1	ER membrane	8.49	Derlin-1: It plays a role in ER-mediated degradation of unfolded proteins and protein transport from ER lumen to the cytoplasm.	[2, 84]

**Q9Y241**	HIGD1A	Mitochondrial inner membrane	8.47	HIG1 domain family member 1A, mitochondrial: It plays role in electron transport chain.	[2, 85]
**Q13951**	CBFB	Nucleus	8.46	Core-binding factor subunit beta: A component of the protein complex that binds to DNA and controls the activity of the gene in cellular developmental process.	[2, 86]
**P14635**	CCNB1	Cytoplasm, Nucleus, Cytoskeleton	8.45	G2/mitotic-specific cyclin-B1: It plays an essential role in mitosis transition of cell cycle.	[2]
**Q96KG9**	SCYL1	Cytoskeleton, Microtubule organizing center	8.44	N-terminal kinase-like protein: It plays a role in COPI mediated protein trafficking and transport of protein between Golgi and endoplasmic reticulum.	[2, 87]
**Q9H0C8**	ILKAP	Cytoplasm	8.43	Integrin-linked kinase-associated serine/threonine phosphatase 2C: A protein phosphatase that plays a role in cell cycle progression.	[2]
**Q9ULT8**	HECTD1	Nucleus	8.40	E3 ubiquitin-protein ligase HECTD1: A ubiquitin protein ligase, which transfers ubiquitin to the target protein and thus helps in protein degradation pathway.	[2, 88]
**P42785**	PRCP	Lysosome, Plasma membrane	8.39	Lysosomal Pro-X carboxypeptidase: A serine type carboxypeptidase, which plays a role in balancing electrolyte in the cell membrane.	[2, 89]
**Q9H2W6**	MRPL46	Mitochondria	8.39	39S ribosomal protein L46, mitochondrial: A mammalian mitochondrial ribosomal protein, which is involved in protein biosynthesis in mitochondria.	[2, 90]
**O43808**	SLC25A17	Cytoplasm, membrane	8.37	Peroxisomal membrane protein PMP34: It plays a role in peroxisomal transport of different cofactor proteins and enzymes.	[2, 91]
**O43847**	NRDC	Cytoplasm, Mitochondria	8.35	Nardilysin: It acts as a hydrolase that cleaves peptide substrate on the N-terminal of arginine.	[2]
**P48729**	CSNK1A1	Cytoplasm, Cytoskeleton	8.34	Casein kinase I isoform alpha: A kinase protein which phosphorylates a large variety of acidic proteins and thus regulates different signaling pathways. It is also involved in the Wnt signaling pathway.	[2, 92]
**Q99797**	MIPEP	Mitochondria	8.33	Mitochondrial intermediate peptidase: It cleaves proteins and thus imports cytoplasmic proteins to the mitochondria to their mature size.	[2, 93]
**Q9GZP4**	PITHD1	Cytoplasm	8.33	PITH domain-containing protein 1: Proteasome interacting molecule, which plays role to regulate protein homeostasis.	[2, 94]

**O95219**	SNX4	Cytoplasm	8.33	Sorting nexin 4: It plays an important role in endocytosis and different steps of intracellular trafficking and recycling.	[2, 95]
**Q5QJE6**	DNTTIP2	Nucleus	8.33	Deoxynucleotidyltransferase terminal-interacting protein 2: It plays a role in transcription regulation.	[2]
**Q9Y6D9**	MAD1L1	Cytoplasm	8.32	Mitotic spindle assembly checkpoint protein MAD1: It plays a role in cell cycle.	[2]
**P19404**	NDUFV2	Mitochondrial inner membrane	8.28	NADH dehydrogenase flavoprotein 2: It plays a role in electron transport chain.	[2]
**O15400**	STX7	Early endosome	8.28	Syntaxin-7: membrane integrated protein: Involved in protein trafficking from plasma membrane to early endosome.	[2, 96]
**Q8N0X7**	SPART	Cytoplasm	8.23	Spartin: It is called microtubule interacting and trafficking molecule regulating endosomal trafficking.	[2, 97]
**Q92599**	SEPTIN8	Cytoplasm, Cytoskeleton	8.23	Septin-8: A cytoskeletal GTPase involved in cytoskeletal organization, protein assembly and other interaction with membrane proteins.	[2, 98, 99]
**P40261**	NNMT	Cytoplasm	8.17	Nicotinamide N-methyltransferase: A methyltransferase that regulates cellular methylation.	[2]
**O43865**	AHCYL1	Cell membrane, ER	8.17	S-adenosylhomocysteine hydrolase-like protein 1: Involved in regulation of mRNA processing.	[2]
**Q8N6T3**	ARFGAP1	Cytoplasm, Golgi apparatus	8.16	ADP-ribosylation factor GTPase-activating protein 1: A GTPase activating protein, which activates ARF1, and thus participates in trafficking of membrane proteins.	[2, 22]
**Q08752**	PPID	Cytoplasm, Nucleus	8.11	Peptidyl-prolyl cis-trans isomerase D: A PPIase protein that helps cis/trans isomerization of proline containing peptide bonds, which regulates protein folding.	[2, 100]
**Q15386**	UBE3C	Proteasome complex	8.10	Ubiquitin-protein ligase E3C: A ubiquitinate to target protein substrates, and thus regulate different cellular processes.	[2]
**P48651**	PTDSS1	ER membrane	8.09	Phosphatidylserine synthase 1: It catalyzes the conversion of phosphatidylcholine or phosphatidylethanolamine to phosphatidylserine.	[2]
**Q9NR33**	POLE4	Nucleus	8.08	DNA polymerase epsilon subunit 4: It regulates DNA repair and chromosomal DNA replication.	[2]
**Q8IXS6**	PALM2	Cell membrane	8.07	Paralemmin-2: It binds to the regulatory subunit of protein kinase A (PKA), and thus is involved in transduce transmembrane signal.	[2]

**P49840**	GSK3A	Cytoplasm	8.06	Glycogen synthase kinase-3 alpha: It regulates glucose homeostasis and participates in Wnt signaling pathway.	[2]
**P53680**	AP2S1	Clathrin coated vesicles	8.06	AP-2 complex subunit sigma: An adaptor protein complex present in cell membrane, which regulates protein transport by clathrin coated cargo formation.	[2]
**Q13617**	CUL2	Nucleus	8.04	Cullin-2: A component of E3 ubiquitin protein ligase complex, which ubiquitinates the target proteins.	[2]
**Q9BZV1**	UBXN6	Cytoplasm, Cytoskeleton, membrane	8.04	UBX domain-containing protein 6: It plays a role in ER associated protein degradation, and thus regulates various signaling pathway.	[2]
**P78316**	NOP14	Nucleus	8.01	Nucleolar protein 14: It plays a role in ribosomal biogenesis and nuclear export of ribosomal subunit.	[2]
**Q9BRP8**	PYM1	Cytoplasm, Nucleus	8.00	Partner of Y14 and mago: A component of protein assembly required to regulate different post-transcriptional processes, e.g., mRNA export or RNA decay etc.	[2]
**Q06587**	RING1	Cytoplasm, Nucleus	7.98	E3 ubiquitin-protein ligase RING1: A components of E3 ubiquitin ligase that ubiquitinates the lysine residue of histone protein and plays a role in gene regulation.	[2]
**Q9UBK8**	MTRR	Cytoplasm	7.98	Methionine synthase reductase: An enzyme responsible for methionine homeostasis.	[2]
**O60784**	TOM1	Cytoplasm, Endosome membrane	7.97	Target of Myb1 membrane trafficking protein: An adaptor protein playing a role in membrane trafficking of ubiquitinated proteins.	[2]
**Q5R3I4**	TTC38	Extracellular exosome	7.96	Tetratricopeptide repeat protein 38: It plays a role in cell cycle.	[2]
**Q96G23**	CERS2	ER membrane	7.96	Ceramide synthase 2: Catalyzing the transfer of acyl chain from acyl-COA to spingoid bases.	[2, 101]
**Q7L2J0**	MEPCE	Nucleus	7.95	7SK snRNA methyl phosphate capping enzyme: A methyl transferase, which helps add methyl phosphate cap at the 5’ end of mRNA.	[2]
**Q9GZZ9**	UBA5	Cytoplasm, ER membrane	7.89	Ubiquitin-like modifier-activating enzyme 5: It interacts with other proteins to regulate the protein function.	[2, 102]
**Q01433**	AMPD2	Cytoplasm	7.87	AMP deaminase 2: It plays a role in nucleotide metabolism.	[2]
**Q96T60**	PNKP	Membrane, mitochondria	7.86	Bifunctional polynucleotide phosphatase/kinase: It plays a role in DNA repair pathway.	[2]

**O43264**	ZW10	ER membrane	7.86	Centromere/kinetochore protein zw10 homolog: Involved in protein trafficking between ER and Golgi.	[2]
**Q9H9Q2**	COPS7B	Cytoplasm, Nucleus	7.85	COP9 signalosome complex subunit 7b: A component of COP9 signalosome complex, and thus involved in different signaling pathway. It regulates ubiquitin conjugation pathway.	[2]
**Q9BZF1**	OSBPL8	ER membrane	7.84	Oxysterol-binding protein-related protein 8: Involved in lipid transport between ER and plasma membrane.	[2]
**Q8NB90**	SPAT5	Cytoplasm, cytoskeleton	7.83	Ribosome biogenesis protein SPATA5: An ATP dependent chaperon protein, which helps in cytoplasmic maturation of ribosomal complexes, and thus regulates late step of ribosomal assembly.	[2, 103]
**P15151**	PVR	Cell membrane	7.82	Poliovirus receptor: It plays a role in transport of endocytic vesicles containing poliovirus viron, and thus acts as receptor for poliovirus.	[2]
**Q14562**	DHX8	Nucleus	7.82	ATP-dependent RNA helicase DHX8: A component of spliceosome complex involved in mRNA splicing.	[2]
**Q14691**	GINS1	Nucleus	7.81	DNA replication complex GINS protein PSF1: Involved in DNA replication.	[2]
**P49902**	NT5C2	Cytoplasm	7.81	Cytosolic purine 5'-nucleotidase: A 5'-nucleotidase that dephosphorylates 6-hydroxypurine nucleoside 5'-monophosphates.	[2]
**Q86X76**	NIT1	Cytoplasm, Mitochondria	7.80	Deaminated glutathione amidase: A protein in carbon-nitrogen hydrolase superfamily, which controls uncontrolled cell proliferation.	[2, 104]
**Q96K76**	USP47	Cytoplasm	7.80	Ubiquitin carboxyl-terminal hydrolase 47: A ubiquitin specific protease that deubiquitinates the DNA polymerase.	[2]
**Q9NP74**	PALMD	Cytoplasm, membrane	7.78	Palmdelphin: A membrane protein involved in plasma membrane-cytoskeleton interaction.	[2, 105]
**Q96BP3**	PPWD1	Nucleus	7.74	Peptidylprolyl isomerase domain and WD repeat-containing protein 1: A PPIase protein that is involved in cis-trans isomerization and regulates protein folding.	[2, 106]
**Q99755**	PIP5K1A	Cell membrane, Cytoplasm	7.72	Phosphatidylinositol 4-phosphate 5-kinase type-1 alpha: It catalyzes the phosphorylation of phosphatidylinositol-4-phosphate to form phosphatidylinositol 4,5-biphosphate, which is an important secondary messenger, and regulates protein trafficking.	[2]

**P23434**	GCSH	Mitochondria	7.72	Glycine cleavage system H protein, mitochondrial: A component of glycine cleavage system.	2
**Q9H3P2**	NELFA	Nucleus	7.71	Negative elongation factor A: A component of negative elongation factor that impacts the transcription.	2
**P21359**	NF1	Nucleus, Cell membrane	7.70	Neurofibromin: A cytoplasmic GAP-like protein, which is involved in the regulation of cell growth.	2, 107
**Q15054**	POLD3	Cytoplasm, Nucleus	7.69	DNA polymerase delta subunit 3: A component of DNA polymerase complex, which regulates DNA synthesis.	2
**Q4V328**	GRIPAP1	Early endosome, recycling endosome	7.68	GRIP1-associated protein 1: It regulates endosomal recycling to the plasma membrane. It is involved in endocytosis of AMPA-type glutamate receptor (AMPAR). Gangliosides are reported to interact with other proteins, which are also involved in AMPAR trafficking.	2, 108, 109
**Q9Y4F1**	FARP1	Cell membrane, cytoskeleton	7.68	FERM, ARHGEF and pleckstrin domain-containing protein 1: Ot regulates Rac1 related signaling pathway by acting as a guanine nucleotide exchange factor for Rac1 protein.	2, 110
**P45985**	MAP2K4	Cytoplasm, Nucleus	7.67	Dual specificity mitogen-activated protein kinase 4: A protein kinase, which acts as essential component of MAP kinase signal transduction pathway.	2, 111
**Q14141**	SEPTIN6	Cytoplasm, cytoskeleton	7.67	Septin-6: A cytoskeletal GTP-binding protein required for organization of cytoskeleton.	2, 112
**Q9NXH9**	TRMT1	Nucleus	7.66	tRNA (guanine (26)-N (2))-dimethyltransferase: It acts as a methylation agent for tRNA molecule.	2
**P49903**	SEPHS1	Cytoplasm	7.65	Selenide, water dikinase 1: It synthesizes selenophosphate.	2
**Q12933**	TRAF2	Cytoplasm	7.65	TNF receptor-associated factor 2: It plays a role in activation of NF-kappa-B, and thus regulates cellular apoptosis and cell survival.	2
**Q9NQW6**	ANLN	actin cytoskeleton	7.62	Anillin: Actin binding protein is involved in cell cycle and cell division.	2
**Q8WXI9**	GATAD2B	Nucleus	7.61	Transcriptional repressor p66-beta: It acts as a transcriptional repressor.	2
**Q9UKZ1**	CNOT11	Cytoplasm, Nucleus	7.59	CCR4-NOT transcription complex subunit 11: A component of CCR4-NOT complex, and it regulates cellular RNA related processes, e.g., RNA degradation, translational repression etc.	2

**P19388**	POLR2E	Nucleus	7.59	DNA-directed RNA polymerases I, II, and III subunit RPABC1: A DNA dependent RNA polymerase, which catalyzes the transcription.	[2]
**Q8NCF5**	NFATC2IP	Nucleus, cytoplasm	7.58	NFATC2-interacting protein: It regulates the transcription of specific subset of cytokine.	[2]
**Q9BWS9**	CHID1	Secreted vesicles, lysosome	7.55	Chitinase domain-containing protein 1: A Lipo polysaccharide (LPS) binding protein, which acts as receptor for different pathogens.	[2, 113]
**Q9NZW5**	PALS2	Plasma membrane	7.54	Protein associated with LIN7 2: A scaffolding protein localized at lateral membrane of kidney cells. A component of mLin-7 complex, which interacts with glycosphingolipids or glycosylphosphatidylinositol to regulate the endocytosis of transmembrane proteins.	[2, 114, 115]
**Q9P0V3**	SH3BP4	Calthrin coated pit	7.53	SH3 domain-binding protein 4: A membrane protein participating in clathrin-mediated endocytosis. It is mainly involved in recognition of different substrates and down regulation of kinase activity.	[2, 116]
**Q6NYC1**	JMJD6	Cytoplasm, Nucleus	7.52	Bifunctional arginine demethylase and lysyl-hydroxylase JMJD6: A lysyl hydroxylase that catalyzes hydroxylation of lysine residue of target protein.	[2, 117]
**Q9H330**	TMEM245	Membrane		Transmembrane protein 245: A not well explored protein, known to regulate cell proliferation.	[2, 118]
**O15061**	SYNM	Cytoplasm, cytoskeleton	7.50	Synemin: Intermediate filament protein, which plays a role in cytoskeleton rearrangement.	[2, 119]
**P22570**	FDXR	Mitochondrial inner membrane	7.49	NADPH: adrenodoxin oxidoreductase: An electron transfer protein in mitochondrial P450 system, which regulates electron transport in cell.	[2]
**P19971**	TYMP	Cytoplasm	7.46	Thymidine phosphorylase: It catalyzes the reversible phosphorolysis of thymidine.	[2]
**Q9P253**	VPS18	Cytoplasmic vesicles, Endosome membrane	7.44	Vacuolar protein sorting-associated protein 18 homolog: An important component of endosomal sorting complex required for transport (ESCRT), which plays a role in vesicle-mediated protein trafficking.	[2, 120]
**Q13601**	KRR1	Cytoplasm, Nucleus	7.42	KRR1 small subunit processome component homolog: A protein involved in ribosomal RNA processing and assembly.	[2, 121]
**Q9BXS6**	NUSAP1	Cytoplasm, Nucleus	7.38	Nucleolar and spindle-associated protein 1: A microtubule associated protein, which helps in organization of mitotic spindle microtubule around chromosome.	[2, 122]

**Q13641**	TPBG	Cell membrane	7.37	Trophoblast glycoprotein: A transmembrane glycoprotein, mainly involved in cell adhesion.	[2, 123]
**Q99808**	SLC29A1	Cell membrane	7.37	Equilibrative nucleoside transporter 1: A multipass membrane protein, regulating cellular uptake of nucleosides.	[2, 124]
**O60443**	GSDME	Cytoplasm, Cell membrane	7.33	Gasdermin-E: A pore forming protein, which binds to the plasma membrane and form pore, triggering pyroptosis.	[2, 125]
**O96017**	CHEK2	Nucleus	7.33	Serine/threonine-protein kinase Chk2: A protein kinase, which regulates check point-mediated cell cycle arrest.	[2, 126]
**Q8NC60**	NOA1	Mitochondrial inner membrane	7.32	Nitric oxide-associated protein 1: Involved in mitochondrial protein translation. It also plays a role in mitochondria-mediated cell death.	[2, 127]
**A6NDU8**	RIMOC1	Cytoplasm	7.30	RAB7A-interacting MON1-CCZ1 complex subunit 1: It plays a role in removal of damaged mitochondria via mitophagy by stabilizing RAB7A.	[2, 128]
**Q8IWX8**	CHERP	Membrane, cytoplasm	7.29	Calcium homeostasis endoplasmic reticulum protein: Involved in calcium homeostasis, cell growth, and proliferation.	[2, 129]
**Q9NXV6**	CDKN2AIP	Nucleus	7.23	CDKN2A-interacting protein: A protein regulating DNA damage response by participating in different signaling pathways.	[2]
**Q14966**	ZNF638	Nucleus	7.18	Zinc finger protein 638: It acts as transcription factor that binds DNA in a sequence specific way and regulates the expression of different genes.	[2, 130]
**Q63HN8**	RNF213	Cytoplasm, lipid droplet	7.14	E3 ubiquitin-protein ligase RNF213: It catalyzes the ubiquitination of different lipids and proteins.	[2, 131]
**Q9Y676**	MRPS18B	Mitochondrial inner membrane	7.06	28S ribosomal protein S18b, mitochondrial: A mitochondrial ribosomal protein that binds to ribosomal subunit.	[2]
**Q53FT3**	HIKESHI	Cytoplasm, Nucleus	7.05	Protein Hikeshi: It acts as specific nuclear import carrier and participates in protein transport.	[2]
**Q9Y487**	ATP6V0A2	Cell membrane, Endosome membrane	7.04	V-type proton ATPase 116 kDa subunit a 2: A subunit of vaccuolar ATPase complex that regulates proton translocation through cell membrane.	[2, 132]
**Q9HA64**	FN3KRP	Cytoplasm	7.00	Ketosamine-3-kinase: A kinase, which shows ATP and nucleotide binding activity.	[2, 133]
**P83881**	RPL36A	Cytoplasm	6.95	60S ribosomal protein L36a: A component of ribonucleoprotein complex, which helps in protein biosynthesis.	[2, 134]

**Q92504**	SLC39A7	Endoplasmic reticulum membrane	6.94	Zinc transporter SLC39A7: It transports Zn^{2+} from ER or Golgi to the cytoplasm, and thus induce cell proliferation, cell migration etc.	[2, 135]
**Q13769**	THOC5	Nucleus, Cytoplasm	6.93	THO complex subunit 5 homolog: A component of THO subcomplex, which is involved in mRNA processing, nuclear export, and related events.	[2, 136]
**Q15654**	TRIP6	Cytoplasm, cytoskeleton	6.93	Thyroid receptor-interacting protein 6: It relays signal from cell surface to the nucleus. This protein is also involved in cytoskeletal organization.	[2, 137]
**Q99707**	MTR	Cytoplasm	6.93	Methionine synthase: It acts as a methyl transferase.	[2]
**Q69YN4**	VIRMA	Cytoplasm, Nucleus	6.90	Protein virilizer homolog: A component of WMM protein complex responsible for methylation of RNA and thus it plays a role in mRNA splicing and RNA processing.	[2, 138]
**Q99543**	DNAJC2	Cytoplasm, Nucleus	6.83	DnaJ homolog subfamily C member 2: It acts as chaperon proteins for ribosome-associated protein complex as well as chromatin regulator.	[2]
**Q9H9T3**	ELP3	Cytoplasm, Nucleus	6.69	Elongator complex protein 3: A subunit of catalytic tRNA acyl transferase subunit of elongator complex, which plays a role in tRNA modification.	[2]
**Q8N543**	OGFOD1	Cytoplasm	6.69	Prolyl 3-hydroxylase OGFOD1: It catalyzes the hydroxylation of proline residue and thus regulates protein translation.	[2]
**P16383**	GCFC2	Cytoplasm, Nucleus	6.66	Intron Large complex component GCFC2: Involved in mRNA splicing and mRNA processing.	[2, 139]
**Q96H55**	MYO19	Actin cytoskeleton	6.41	Unconventional myosin-XIX: An actin-based motor molecule that plays a role in mitochondrial dynamics and function.	[2, 140]
**Q96BQ5**	CCDC127	Nucleus	6.25	Coiled-coil domain-containing protein 1: A not well explored protein having functions in regulating chaperon proteins.	[2, 141]
**Q86TU7**	SETD3	Cytoplasm, Nucleus	6.20	Actin-histidine N-methyltransferase: A protein showing actin binding and methyl transferase activity.	[2, 142]
**Q9NRY5**	FAM114A2	Nucleus	6.16	Protein FAM114A2: A not well-known protein having a role in nucleotide binding.	[2]
**O95248**	SBF1	Cytoplasm	6.12	Myotubularin-related protein 5: It acts as guanine nucleotide exchange factor (GEF) for Rab28 protein, and thus regulates Rab protein related signaling pathway.	[2, 143]

**Q9Y232**	CDYL	Nucleus	6.07	Chromodomain Y-like protein: It ats as chromatin reader, recognizes methylation of Histone protein and thus regulates transcription.	[2, 144]
**Q96HP4**	OXNAD1	Plasma membrane	5.89	Oxidoreductase NAD-binding domain-containing protein 1: A not well explored proteins, showing oxidoreductase activity.	[2]
**Q9UHR4**	BAIAP2L1	Cytoplasm, Cytoskeleton	5.36	Brain-specific angiogenesis inhibitor 1-associated protein 2-like protein 1: An adapter protein playing a role in actin cytoskeleton organization.	[2]
**O14732**	IMPA2	Cytoplasm	5.19	Inositol monophosphatase 2: It shows hydrolase activity towards different lipid/sugar phosphates.	[2]
**Q5ST30**	VARS2	Mitochondria	4.96	Valine tRNA ligase, mitochondrial: An aminoacyl-tRNA synthetase, responsible for mitochondrial translation.	[2]
**Q96QU8**	XPO6	Nucleus, Cytoplasm	4.85	Exportin-6 Protein: It recognizes the transport signal in the proteins to transport them from nucleus to cytoplasm.	[2, 145]
**Q66K14**	TBC1D9B	Membrane	4.72	TBC1 domain family member 9B: A single pass membrane protein, which helps activate GTPase for Rab family of proteins.	[2, 146]
**Q9NWW5**	CLN6	Endoplasmic reticulum membrane	3.9	Ceroid-lipofuscinosis neuronal protein 6: It regulates protein transportation from ER to lysosome.	[2, 147]

Table S3. Significantly enriched proteins pulled down by **1** that are potentially related to GSLs.

Gene Accession	Uniprot ID	Literature reported functions potentially related to GSLs	Ref
Q07960<br>Q68EM7<br>Q8IXI1<br>P43487<br>P46060<br>P46940<br>P62826<br>Q13283<br>Q15382<br>P01111<br>Q8N6H7	ARHGAP1<br>ARHGAP17<br>RHOT2<br>RANBP1<br>RANGAP1<br>IQGAP1<br>RAN<br>G3BP1<br>RHEB<br>NRAS<br>ARFGAP2	*Ras-related GTPase activating proteins (GAPs)*: These proteins contain Ras or Ras related (e.g., Ran, Ras-related nuclear proteins; Rho, Ras homologous) GTPase activating proteins that participate in different signaling pathways related to cell proliferation, survival, migration, and cytoskeletal rearrangement. Ras is a monomeric G-protein attached to the inner leaflet of glycosphingolipid-enriched membrane microdomains. The activation of Ras proteins is a starting point for MAPK, PI3K, PLC, and many other pathways related to cell cycle regulation. Ras becomes activated when it binds to active GTP but becomes inactive when binds to GDP. The activity enforced by different Ras-GTPase helps to shuttle off Ras signaling by making it as GDP bound form. Different sphingolipids are believed to relay extracellular signal to the cytoplasm through Ras-GTPase proteins.	148-152
P04083<br>P07355<br>P08133<br>P08758<br>P09525<br>P12429<br>P20073<br>P50995	ANXA1<br>ANXA2<br>ANXA6<br>ANXA5<br>ANXA4<br>ANXA3<br>ANXA7<br>ANXA11	*Annexin proteins*: Annexins are a membrane scaffold protein superfamily, which interact with membrane lipids in a calcium dependent manner. They are involved in a wide variety of intra- and extracellular processes including membrane trafficking, membrane-cytoskeletal rearrangement, ion channel activity regulation, etc. Annexins bind to phosphatidylserine, which presents in the inner leaflet of the glycosphingolipid-enriched membrane microdomain, and thus initiate different signaling pathway by concomitant clustering of rafts and F-actin.	153-156
P28288<br>O75027<br>P61221<br>Q8NE71<br>Q9NRK6<br>Q9NUQ8<br>Q9UG63	ABCD3<br>ABCB7<br>ABCE1<br>ABCF1<br>ABCB10<br>ABCF3<br>ABCF2	*ATP-binding cassette (ABC) transporter proteins*: An ATP binding protein family. They transport phospholipids like glucosylceramide and glycosphingolipids, leading to membrane organization.	157-161
Q12907<br>O60763<br>Q8WUX9<br>Q9H444<br>Q9NZZ3<br>Q9UQN3	LMAN2<br>USO1<br>CHMP7<br>CHMP4B<br>CHMP5<br>CHMP2B	*Vesicular integral-membrane proteins (VIP) and charged multivesicular body proteins*: They are charged multivesicular body proteins acting as components of endosomal sorting complex required for transport (ESCRT), which is necesaary for membrane remodeling in eukaryotes.	
P18085<br>P61204<br>P62330	ARF4<br>ARF3<br>ARF6	*ADP-ribosylation factors (ARF)*: These are ADP-ribosylation factor family of small GTP-binding proteins, which play a role in lipid trafficking at the sites of contact between ER and other organelles. ARF proteins recruit other proteins to mediate transfer of	162

		sphingolipid precursors and sterols, which in turn helps regulate characteristic lipid environment of trans-Golgi, plasms membrane, and endosomal system.	
P20339<br>P20340<br>P29373<br>P31150<br>P50395<br>P51148<br>P51149<br>P51153<br>P61006<br>P61019<br>P61020<br>P61026<br>P61106<br>P62820<br>Q3YEC7<br>Q9H0U4<br>Q9H2M9<br>Q9NP72<br>Q9P260<br>Q9P2R3	RAB5A<br>RAB6A<br>CRABP2<br>GDI1<br>GDI2<br>RAB5C<br>RAB7A<br>RAB13<br>RAB8A<br>RAB2A<br>RAB5B<br>RAB10<br>RAB14<br>RAB1A<br>RABL6<br>RAB1B<br>RAB3GAP2<br>RAB18<br>RELCH<br>ANKFY1	*Rab related Proteins*: These are small GTPase proteins involved in vesicle trafficking of different proteins and lipids. Glycosphingolipids are internalized via a caveolae-dependent mechanism, which are then targeted to the Golgi apparatus by a process dependent on different Rab proteins.	163-165
O00425<br>P51858<br>P62993<br>Q969H8<br>Q9NZI8<br>Q9NZT2<br>Q9Y6B6	IGF2BP3<br>HDGF<br>GRB2<br>MYDGF<br>IGF2BP1<br>OGFR<br>SAR1B	*Different growth factor proteins*: In various signaling pathways, gangliosides control the activity of various receptor tyrosine kinases, including the fibroblast growth factor receptor, neurotrophins receptors, hepatocyte growth factor receptor, platelet-derived growth factor receptor, vascular endothelial growth factor receptor, and insulin receptor.	152, 166-168
Q9NR31	SAR1A	*GTP binding protein SAR1A*: A component of Ras superfamily proteins, which regulates intra-Golgi trafficking of sphingolipids.	169, 170
P50225	SULT1A1	*Sulfotransferase*: A metabolizing enzyme highly expressed in liver, which regulates metabolism and trafficking of different glycolipids and small molecules.	171
P53365	ARFIP2	*Arfaptin-2*: It plays a role in metalloproteinase secretion in the trans-Golgi network by binding to ceramides and the glucosylceramide backbone of different sphingolipids.	172

Table S4. The 105 unique proteins pulled down from HEK293 cells by **2** that were found in all three experiments.

Proteins	Genes	Cellular locations	Protein names and reported or predicted biological functions
**A0MZ66**	SHTN1	Microtubule cytoskeleton	Shootin-1: A cytoskeletal protein involved in cytoskeletal organization through the activation of CDC42 and RAC1 proteins.[2]
**A6NIH7**	UNC119B	Cell projection	Protein unc-119 homolog B: Long chain fatty acid binding protein that acts as cargo adapter for trafficking of membrane proteins.[173]
**A8MXV4**	NUDT19	Peroxisome	Acyl-coenzyme A diphosphatase NUDT19: It acts as a diphosphatase enzyme for fatty acyl-coenzyme A (CoA) and thus regulates fatty acid catabolic processes.[2, 174]
**O00161**	SNAP23	Cell membrane	Synaptosomal-associated protein 23: It regulates membrane fusion machinery and vesicular transport, fusion, and phagosome formation/maturation.[2, 175]
**O00165**	HAX1	Actin cytoskeleton	HCLS1-associated protein X-1: It recruits Arp2/3 complex to the cellular cortex and thus is involved in the organization of actin cytoskeleton to regulate clathrin-mediated endocytosis.[2, 176]
**O00186**	STXBP3	Plasma membrane	Syntaxin-binding protein 3: Involved in SNARE protein complex formation to regulate the vesicle formation and fusion processes at the membrane.[2, 177]
**O00233**	PSMD9	Cytoplasm	26S proteasome non-ATPase regulatory subunit 9: It acts as a chaperone protein and thus regulates the assembly of 26S proteasome complex.[2, 178]
**O00267**	SUPT5H	Nucleus	Transcription elongation factor SPT5: It regulates mRNA processing and RNA polymerase activity.[2, 179]
**O00330**	PDHX	Mitochondrial matrix	Pyruvate dehydrogenase protein X component, mitochondrial: An E3-binding protein that regulates cellular respiration.[2, 180]
**O14656**	TOR1A	Cytoskeleton	Torsin-1A: A protein with chaperon functions, which helps in proper protein folding and localization.[2]
**O14949**	UQCRQ	Mitochondrial inner membrane	Cytochrome b-c1 complex subunit 8: A component of cytochrome C oxidoreductase to play a role in mitochondrial electron transport chain.[2]
**O14975**	SLC27A2	ER lumen	Long-chain fatty acid transport protein 2: It mediates the cell membrane localization of long chain fatty acid across the cell membrane and thus fatty acid metabolism.[2, 181]
**O15031**	PLXNB2	Plasma membrane	Plexin-B2: A cell surface receptor, which plays an important role in cell-cell communication by regulating the activity of CDC42 and RAC1 [2, 182]
**O15091**	PRORP	Mitochondrial matrix	Mitochondrial ribonuclease P catalytic subunit: A catalytic ribonuclease component of mitochondrial ribonuclease P, which plays a role in RNA processing-related functions.[2, 183]

**O15160**	POLR1C	Cytoplasm	DNA-directed RNA polymerases I and III subunit RPAC1: It acts as DNA-dependent RNA polymerase, which catalyzes the transcription of DNA into RNA.[2]
**O15228**	GNPAT	Cell membrane	Dihydroxyacetone phosphate acyltransferase: An acyltransferase responsible for lipid biosynthesis and thus membrane organization.[2]
**O15460**	P4HA2	ER lumen	Prolyl 4-hydroxylase subunit alpha-2: It catalyzes the post-translational modification of proline residue for different ER-resident proteins and thus regulates their cellular localization.[2]
**O43290**	SART1	Cytoplasm	U4 tri-snRNP-associated protein 1: It plays a role in mRNA splicing as a component of spliceosome complex.[2, 184]
**O43432**	EIF4G3	Cytoplasm	Eukaryotic translation initiation factor 4 gamma 3: A component of translation factor showing RNA binding activity.[2, 185]
**O43464**	HTRA2	Plasma membrane-adjacent cytoskeleton	Serine protease HTRA2, mitochondrial: A serine protease that induces cell death.[2, 186]
**O43583**	DENR	Nucleus	Density-regulated protein: A protein involved in ribosome disassembly and translation-related activity.[2]
**O43663**	PRC1	Cytoplasm, cytoskeleton	Protein regulator of cytokinesis 1: It regulates the microtubule-related organization in the cytoplasm.[2]
**O43681**	GET3	ER membrane	ATPase GET3: An ATPase required for post-translational delivery of lipid-anchored protein to the ER.[2]
**O43760**	SYNGR2	Cell membrane	Synaptogyrin-2: It plays a role in cellular exocytosis.[2]
**O43813**	LANCL1	Cytoplasm, Cell membrane	Glutathione S-transferase LANCL1: It regulates G-protein coupled receptor (GPCR)-related activities.[2, 187]
**O43815**	STRN	Cell-cell tight junction	Striatin: A Calmodulin-binding protein, which functions in Ca^{2+}-related signaling proteins.[2]
**O43818**	RRP9	Nucleus	U3 small nucleolar RNA-interacting protein 2: A component of small ribonucleoprotein that participates in modification of ribosomal RNA.[2, 188]
**O43819**	SCO2	Mitochondrial membrane	Protein SCO2 homolog, mitochondrial: A metallochaperone protein required for cytochrome C-related function.[2, 189]
**O60437**	PPL	Cell membrane	Periplakin: Involved in AKT signaling pathways.[2, 190]
**O60488**	ACSL4	Mitochondrial outer membrane	Long-chain-fatty-acid CoA ligase 4: It catalyzes the conversion of long-chain fatty acid for the biosynthesis and regulation of cellular lipids.[2, 191]
**O60518**	RANBP6	Cytoplasm	Ran-binding protein 6: It plays a key role in transport mechanism of nuclear proteins.[2]

**O75122**	CLASP2	Cytoskeleton	CLIP-associating protein 2: Involved in the stabilization of microtubule assembly.[2, 192]
**O75127**	PTCD1	Mitochondrial matrix	Pentatricopeptide repeat-containing protein 1, mitochondrial: A mitochondrial protein involved in regulation of leucine tRNA level.[2, 193]
**O75381**	PEX14	Peroxisome membrane	Peroxisomal membrane protein PEX14: A component of translocon channel protein, which regulates the transportation of peroxisomal cargo proteins.[2, 194]
**O75530**	EED	Nucleus	Polycomb protein EED: Involved in DNA methyltransferase activities.[2, 195]
**O75569**	PRKRA	Cytoplasm	Interferon-inducible double-stranded RNA-dependent protein kinase activator A: It regulates translation-related cellular activities.[2, 196]
**O75608**	LYPLA1	Cell membrane, Cytoplasm	Acyl-protein thioesterase 1: It acts as protein thioesterase.[2, 197]
**O75794**	CDC123	Cytoplasm	Cell division cycle protein 123 homolog: It regulates cell cycle-related process.[2]
**O94903**	PLPBP	Cytoplasm	Pyridoxal phosphate homeostasis protein: It regulates pyridoxal phosphatase homeostasis-related function.[2]
**O94925**	GLS	Mitochondrial matrix	Glutaminase kidney isoform, mitochondrial: It plays a role in regulating the neurotransmitter activity at the brain cells.[2]
**O94927**	HAUS5	Cytoplasm, Cytoskeleton	HAUS augmin-like complex subunit 5: It plays a key role in mitotic spindle assembly and thus regulates the cell division.[2]
**O95251**	KAT7	Cytoplasm	Histone acetyltransferase KAT7: It regulates DNA replication, DNA damage-related processes.[2]
**O95299**	NDUFA10	Mitochondrial matrix	NADH dehydrogenase [ubiquinone] 1 alpha subcomplex subunit 10, mitochondrial: Involved in electron transport chain.[2, 198]
**O95376**	ARIH2	Cytoplasm	E3 ubiquitin-protein ligase ARIH2: Involved in ubiquitination of the target protein in the cytoplasm.[2, 199]
**O95721**	SNAP29	Cytoplasm, Golgi membrane	Synaptosomal-associated protein 29: A SNARE protein, which regulates the vesicle fusion with the lysosomal membrane.[2, 200]
**O95817**	BAG3	Nucleus, Cytoplasm	BAG family molecular chaperone regulator 3: It acts as chaperon for different chaperon proteins and thus regulates the signaling pathways.[2, 201]
**O96005**	CLPTM1	Plasma membrane	Putative lipid scramblase CLPTM1: A multi-pass membrane protein, which is required for GPI anchor biosynthesis in ER.[2, 202]
**P00813**	ADA	Cell surface	Adenosine deaminase: It catalyzes the metabolism and thus regulates the homeostasis of different nucleotides in cells.[2]

**P02538**	KRT6A	Cytoplasm	Keratin, type II cytoskeletal 6A: It regulates epithelial cell migration.[2]
**P02765**	AHSG	Secreted proteins	Alpha-2-HS-glycoprotein: It regulates cellular endocytosis.[2]
**P05166**	PCCB	Cytoplasm, Mitochondrial matrix	Propionyl-CoA carboxylase beta chain, mitochondrial: Involved in catabolism of odd-chain fatty acid.[2, 203]
**P06396**	GSN	Cytoplasm, Cytoskeleton	Gelsolin: It regulates actin organization at the membrane-adjacent cytoskeleton and thus regulates different signaling pathways.[2, 204]
**P08729**	KRT7	Cytoplasm	Keratin, type II cytoskeletal 7: It regulates DNA synthesis in the cells.[2]
**P08754**	GNAI3	Cytoplasm, membrane	Guanine nucleotide-binding protein G(i) subunit alpha-3: A G protein present in the cell membrane that acts as a downstream signal transducer of GPCR proteins.[2, 205]
**P29992**	GNA11	Plasma membrane	Guanine nucleotide-binding protein (G protein) subunit alpha-11: A G protein, which is involved as a modulator for various transmembrane signaling pathways.[2]
**P10253**	GAA	Lysosomal membrane	Lysosomal alpha-glucosidase: It plays a role in degradation of glycogen in lysosome.[2, 206]
**P10619**	CTSA	Lysosome	Lysosomal protective protein: It regulates the activity of beta-galactosidase and neuraminidase.[2, 207]
**P11441**	UBL4A	Cytoplasm, Nucleus	Ubiquitin-like protein 4A: It plays a role in cytoplasmic quality control mechanism.[2, 208]
**P14174**	MIF	Cytoplasm	Macrophage migration inhibitory factor: A inflammatory cytokine protein involved in innate immune response for bacterial pathogen.[2, 209]
**P15291**	B4GALT1	Golgi plasma membrane	Beta-1,4-galactosyltransferase 1: It catalyzes the biosynthesis of complex oligosaccharide at Golgi for protein or lipid post-translational modification.[2, 210]
**P18859**	ATP5PF	Mitochondrial inner membrane	ATP synthase-coupling factor 6, mitochondrial: It regulates proton-mediated ATP synthesis across the mitochondrial membrane.[2]
**P19784**	CSNK2A2	Cytoplasm	Casein kinase II subunit alpha: A serine/threonine protein kinase that regulates the intracellular signaling pathways by phosphorylation of the target proteins.[2, 211]
**P20338**	RAB4A	Plasma membrane	Ras-related protein Rab-4A: A small GTPase involved in vesicular transport of cellular proteins.[2, 212]
**P20645**	M6PR	Trans-Golgi network	Cation-dependent mannose-6-phosphate receptor: It plays a role in the transport of cell membrane proteins to the lysosome.[2, 213]
**P26440**	IVD	Mitochondrial matrix	Isovaleryl-CoA dehydrogenase, mitochondrial: It regulates the catabolic pathways of leucine amino acid.[2, 214]

**P27338**	MAOB	Mitochondrial outer membrane	Amine oxidase [flavin-containing] B: It catalyzes the oxidative deamination of small molecules.[2]
**P27361**	MAPK3	Cytoskeleton	Mitogen-activated protein kinase 3: A serine/threonine protein kinase, which is involved in MAPK kinase signal transduction pathway.[2, 215]
**P29372**	MPG	Cytoplasm, mitochondrial matrix	DNA-3-methyladenine glycosylase: It mediates the hydrolysis of deoxyribose N-glycosidic bond, and thus regulates different signaling pathways.[2]
**P30043**	BLVRB	Plasma membrane	Flavin reductase (NADPH): An oxidoreductase that catalyzes NADPH-dependent reduction of variety of flavin proteins.[2, 216]
**P32456**	GBP2	Cytoplasm	Guanylate-binding protein 2: A cell membrane-adjacent GTPase, which regulates different signaling pathways.[2, 217]
**P33527**	ABCC1	Cell membrane	Multidrug resistance-associated protein 1: A multipass membrane protein that regulates the export of small molecules and ions from the cytoplasm.[2, 218]
**P36405**	ARL3	Golgi membrane	ADP-ribosylation factor-like protein 3: A GTP-binding protein, which is involve in different GTPase-protein activation-mediated signaling pathways.[2, 219]
**P38435**	GGCX	ER membrane	Vitamin K-dependent gamma-carboxylase: It catalyzes the gamma-carboxylation of different signaling proteins.[2, 220]
**P40424**	PBX1	Cytoplasm	Pre-B-cell leukemia transcription factor 1: It acts as a transcription factor.[2]
**P41223**	BUD31	Nucleus	Protein BUD31 homolog: Involved in mRNA splicing process.[2]
**P42566**	EPS15	Plasma membrane	Epidermal growth factor receptor substrate 15: Involved in receptor tyrosine kinase (RTK)-mediated signaling pathways by regulating the endocytosis of RTK receptor.[2]
**P42574**	CASP3	Cytoplasm	Caspase-3: A thiol protease that regulates caspase-related signaling pathways.[2, 221]
**P42694**	HELZ	Membrane	Probable helicase with zinc finger domain: A helicase protein that is involved in RNA metabolism.[2]
**P45954**	ACADSB	Mitochondrial matrix	Short/branched chain specific acyl-CoA dehydrogenase, mitochondrial: It catalyzes the dehydrogenation of fatty acyl-CoA thioester.[2]
**P46063**	RECQL	Plasma membrane	ATP-dependent DNA helicase Q1: Involved in DNA repair mechanism.[2]
**P46734**	MAP2K3	Cytoplasm, membrane	Dual specificity mitogen-activated protein kinase 3: A kinase that regulates phosphorylation of serine/threonine residue of different proteins and different signaling pathways.[2, 222]
**P46939**	UTRN	Cytoskeleton	Utrophin: It plays a role in attachment of cytoskeleton to the plasma membrane and thus regulates the signaling mechanism involving cytoskeleton rearrangement.[2]

**P48730**	CSNK1D	ER-Golgi intermediate compartment	Casein kinase I isoform delta: A serine/threonine protein kinase that regulates different signaling pathways by mediating protein phosphorylation.[2, 223]
**P49750**	YLPM1	Nucleus	YLP motif-containing protein 1: It plays a role in transcription-related processes.[2]
**P49821**	NDUFV1	Mitochondrial inner membrane	NADH dehydrogenase [ubiquinone] flavoprotein 1, mitochondrial: It plays a role in mitochondrial electron transport chain.[2]
**P49841**	GSK3B	Cytoplasm	Glycogen synthase kinase-3 beta: A protein kinase involved in variety of signaling pathways.[2, 224]
**P51809**	VAMP7	Cytoplasm, ER membrane	Vesicle-associated membrane protein 7: Involved in vesicular fusion of proteins in their early secretory pathways.[2]
**P51946**	CCNH	Nucleus	Cyclin-H: It regulates the activation of cyclin associated kinase via the phosphorylation of different proteins.[225]
**P52758**	RIDA	Mitochondrial matrix	2-iminobutanoate/2-iminopropanoate deaminase: It catalyzes the hydrolytic deamination of different metabolic precursor.[2, 226]
**P52895**	AKR1C2	Cytoplasm	Aldo-keto reductase family 1 member C2: A keto reductase involved in NADH- or NADPH-dependent reduction mechanism in different signaling pathways.[2]
**P55081**	MFAP1	Centrosome	Microfibrillar-associated protein 1: Involved in mRNA splicing mechanism.[2]
**P56182**	RRP1	Nucleus	Ribosomal RNA processing protein 1 homolog A: Involved in rRNA biosynthesis and related signaling pathways.[2, 227]
**P56385**	ATP5ME	Mitochondrial inner membrane	ATP synthase subunit e, mitochondrial: A mitochondrial membrane ATP synthase, which is involved in electron transport mechanism.[2]
**P60468**	SEC61B	ER membrane	Protein transport protein Sec61 subunit beta: A channel forming protein involved in protein transport and translocation.[2, 228]
**P60903**	S100A10	Cell surface	Protein S100-A10: Involved in regulation of protein phosphorylation.[2]
**P60983**	GMFB	not known	Glia maturation factor beta: Involved in cell proliferation.[2]
**P61966**	AP1S1	Clathrin-coated pits, early endosome	AP-1 complex subunit sigma-1A: Involved in protein transport mechanism at the trans-Golgi network or early endosome.[2]
**P78310**	CXADR	Plasma membrane	Coxsackievirus and adenovirus receptor: Involved in epithelial cell migration and cell adhesion-related processes.[2]
**P82673**	MRPS35	Mitochondrial inner membrane	Small ribosomal subunit protein mS35: Involved in mitochondrial translation.[2]

**P98194**	ATP2C1	ER, Cis-Golgi network	Calcium-transporting ATPase type 2C member 1: An ATP-driven ion pump that regulates the ion balance to Golgi.[2, 229]
**Q03164**	KMT2A	Nucleus	Histone-lysine N-methyltransferase 2A: A methyltransferase involved in protein binding and thus involved in chromatin regulation.[2]
**Q03518**	TAP1	ER membrane	Antigen peptide transporter 1: A transport protein involved in antigen or protein transport from the cytoplasm to the ER.[2, 230]
**Q05209**	PTPN12	Cytoplasm, nucleus	Tyrosine-protein phosphatase non-receptor type 12: It dephosphorylates a variety of proteins and thus regulates different signaling pathways.[2, 231]
**Q06265**	EXOSC9	RNA-exosome complex	Exosome complex component RRP45: A component of the RNA-exosome complex, which is involved in RNA processing.[2]
**Q12756**	KIF1A	Microtubule	Kinesin-like protein KIF1A: Involved in calcium ion-dependent vesicle transport of scaffold proteins.[2, 232]

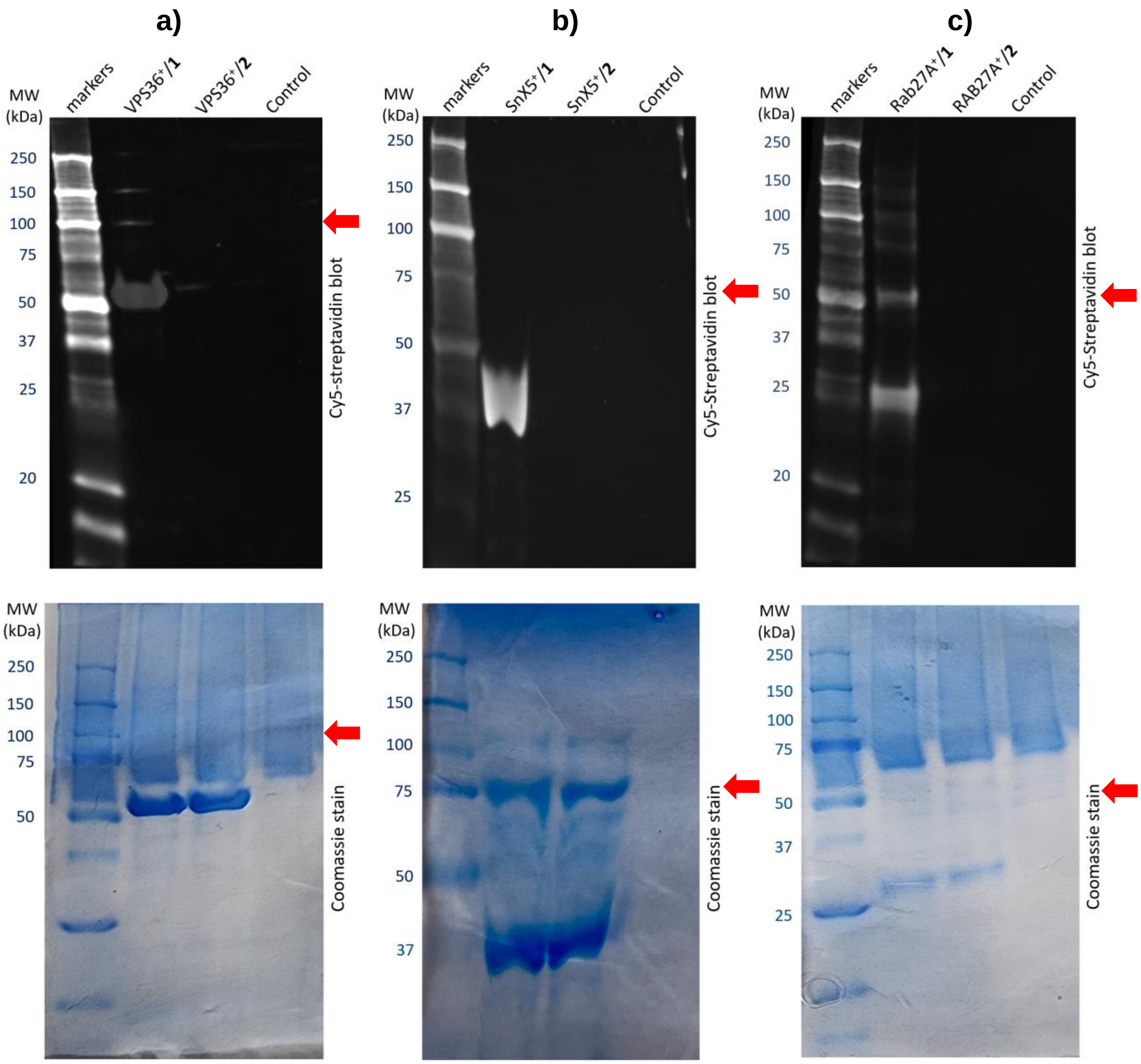

Figure S16: Cy5-streptavidin blot images (top) showing the anti-FLAG resin-isolated proteins that are labeled by **1** or **2**, from **a**) FLAG-VPS36, **b**) FLAG-SNX5, and **c**) Rab2A overexpressing HEK293 cells incubated with **1** (protein$^+$/**1**) and **2** (protein$^+$/**2**) or from wild-type cells (control) after UV light-mediated protein crosslink and click-mediated biotinylation, as well as the corresponding Coomassie stain images (bottom) revealing all proteins, including proteins that are not labeled by **1** or **2** but have nonspecific binding to the resin, which only serves as validation that similar amounts of proteins are applied to different lanes on each gel, while one lane (+**1**) is blotted by Cy5-streptavidin but not the other lane (+**2**). (Red arrows indicate the positions of the dimers of target proteins.) Figure 5b in the main text and Figure S16 here represent the same data, but Figure 5b shows only the relevant sections of the whole gel images.

II. NMR and MS Spectra of All New Compounds

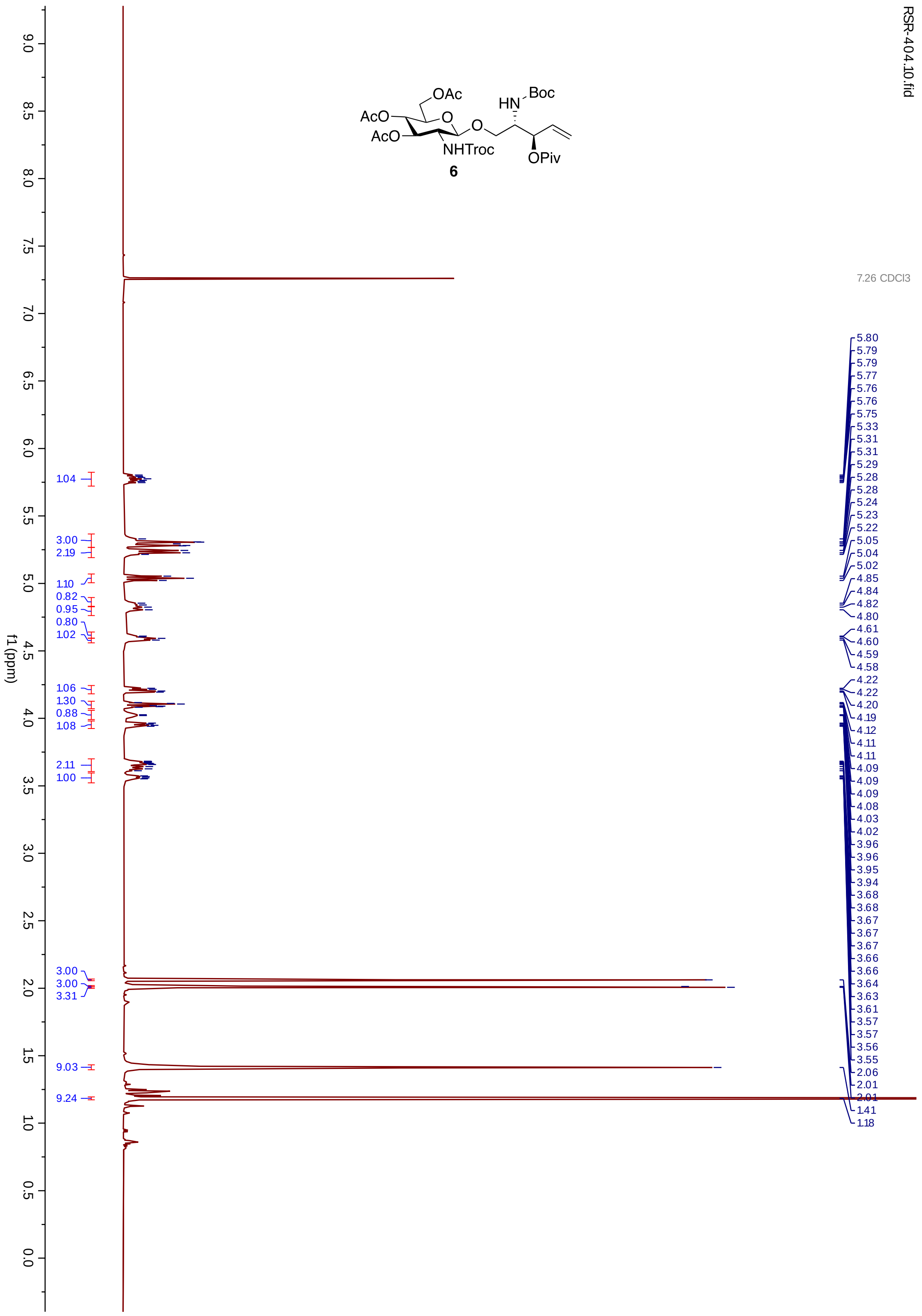

Figure 17: ^{1}H NMR of compound **6** (600 MHz, $CDCl_3$).

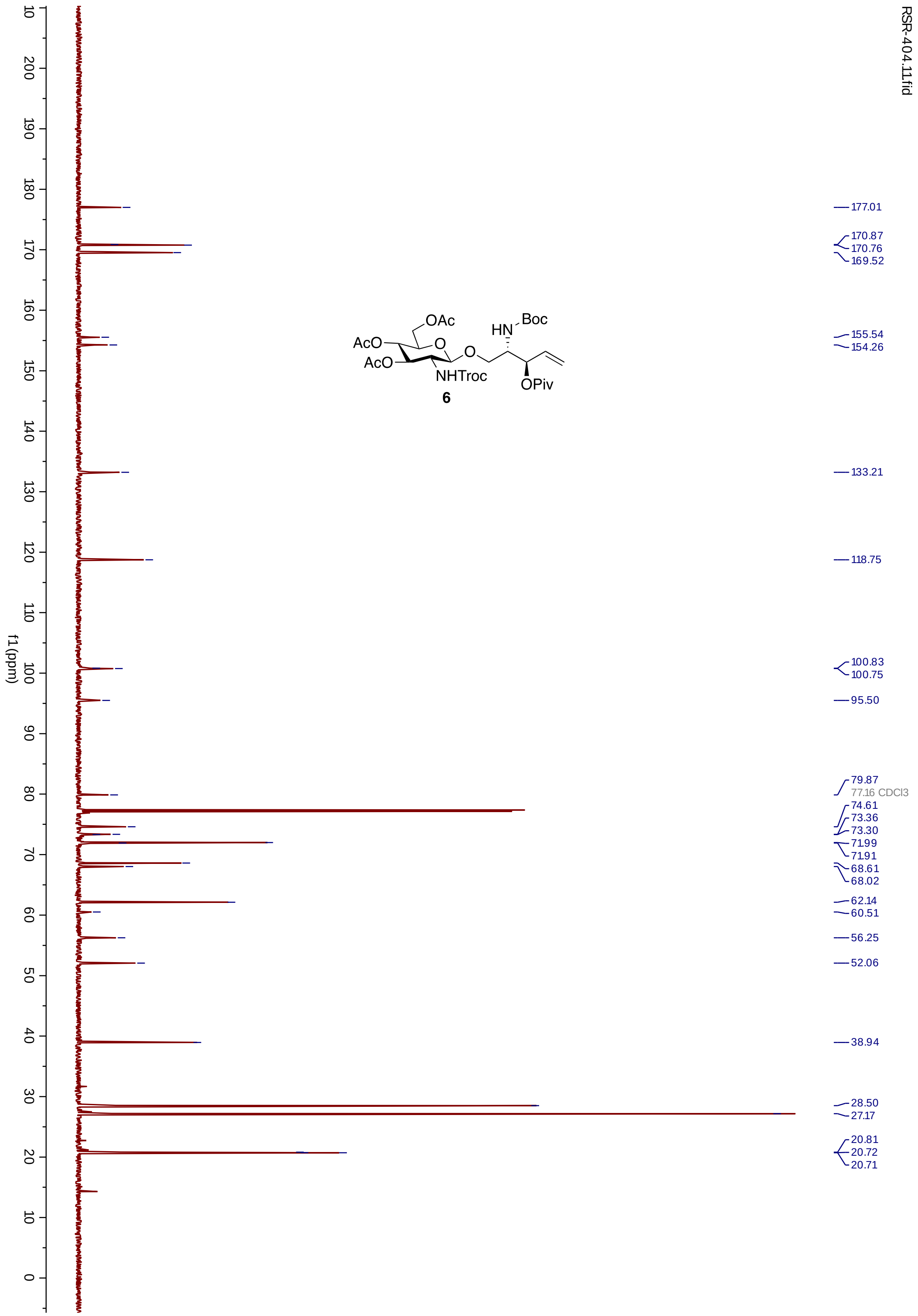

Figure 18: $^{13}C\{^{1}H\}$ NMR of compound **6** (150 MHz, $CDCl_3$).

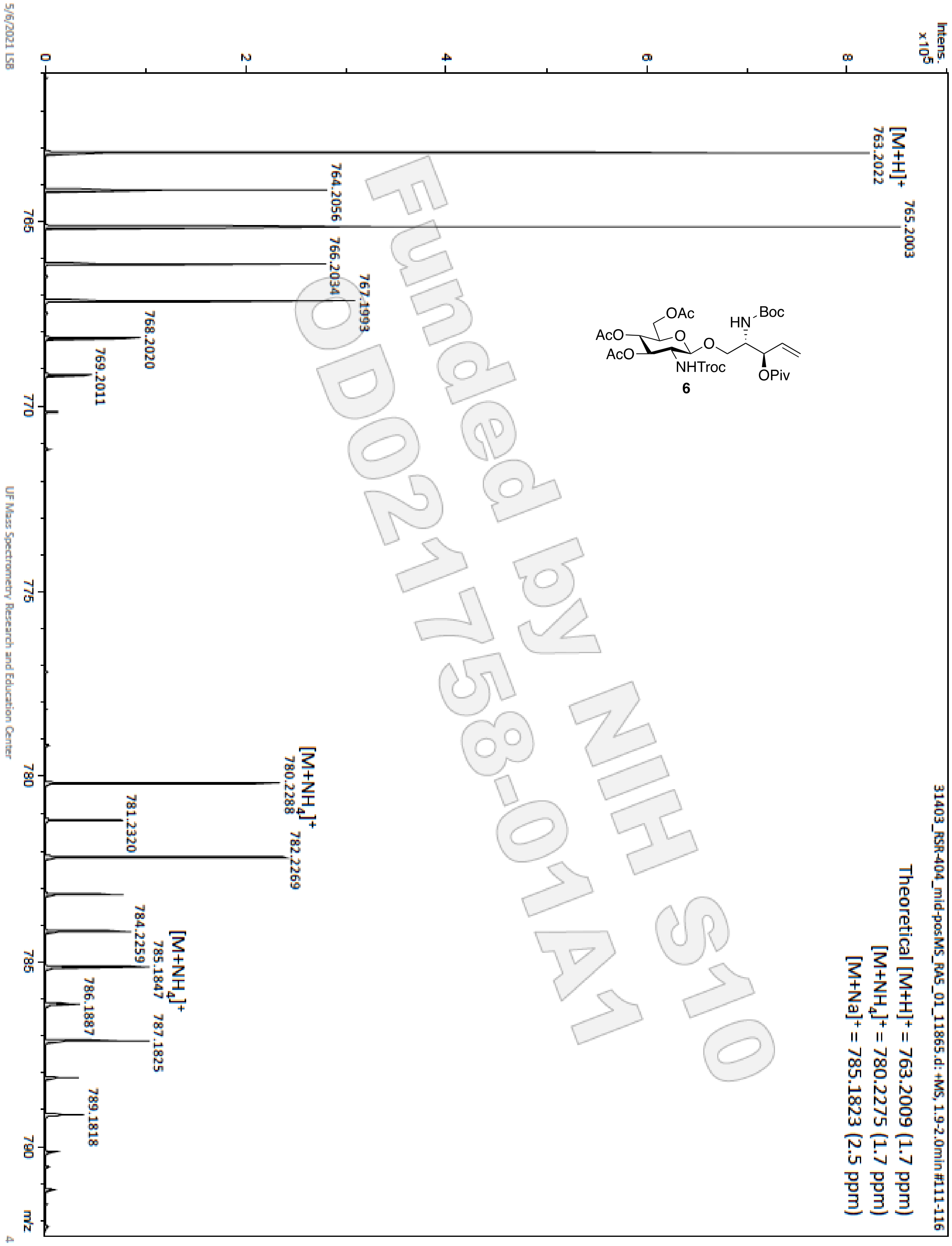

Figure 19: HRMS ESI-TOF of compound **6.**

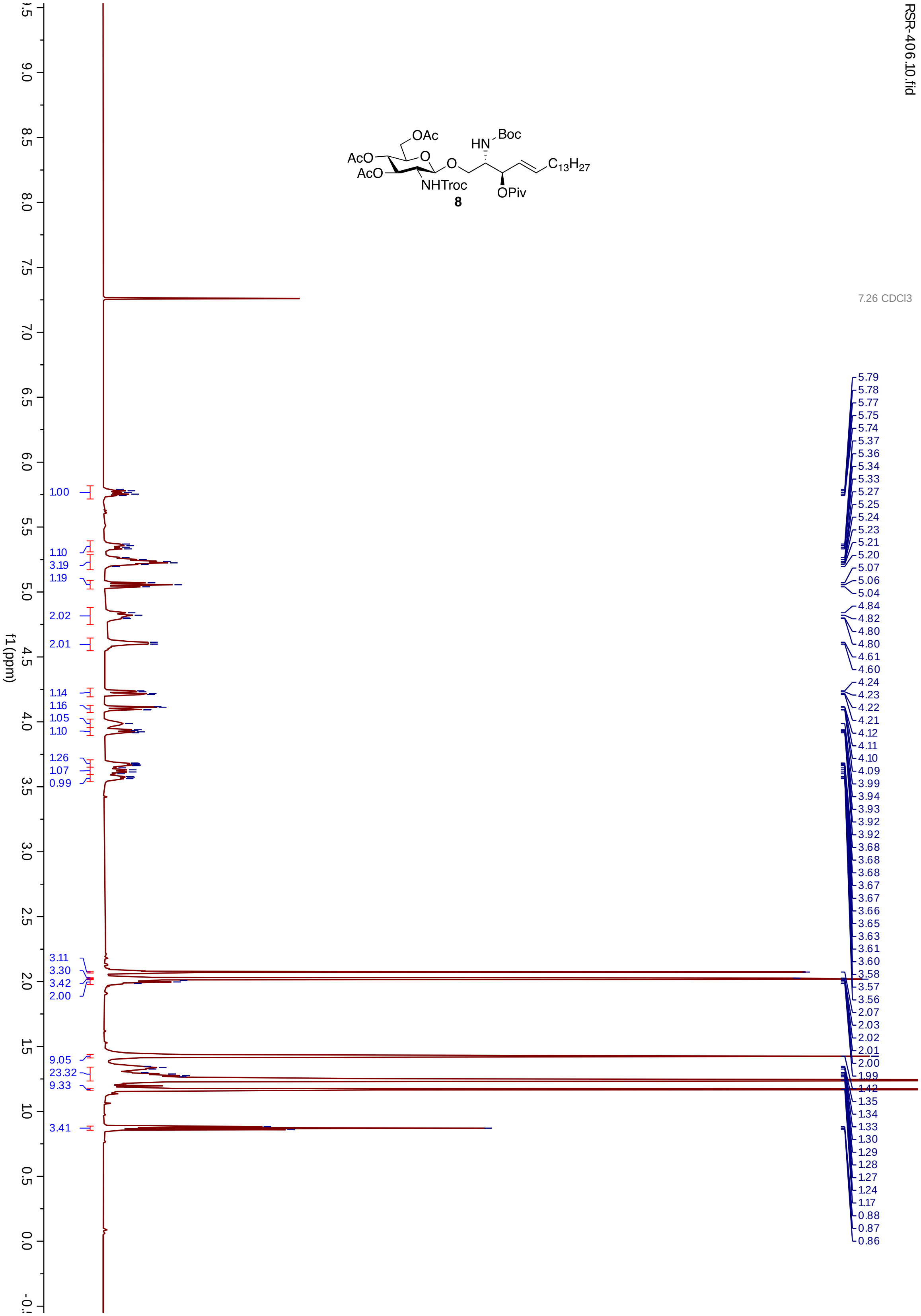

Figure 20: ^{1}H NMR of compound **8** (600 MHz, $CDCl_3$).

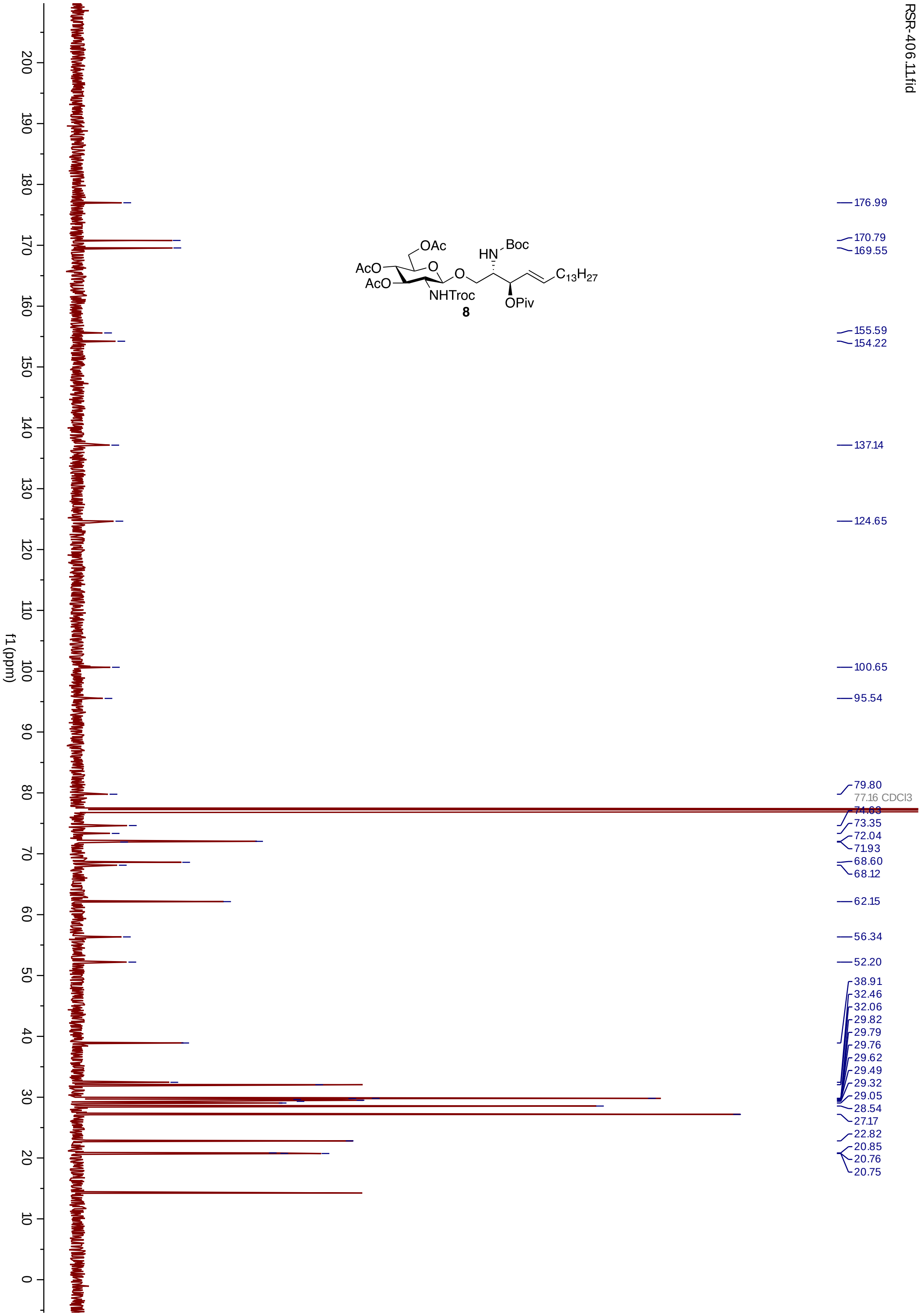

Figure 21: $^{13}C\{^{1}H\}$ NMR of compound **8** (150 MHz, $CDCl_3$).

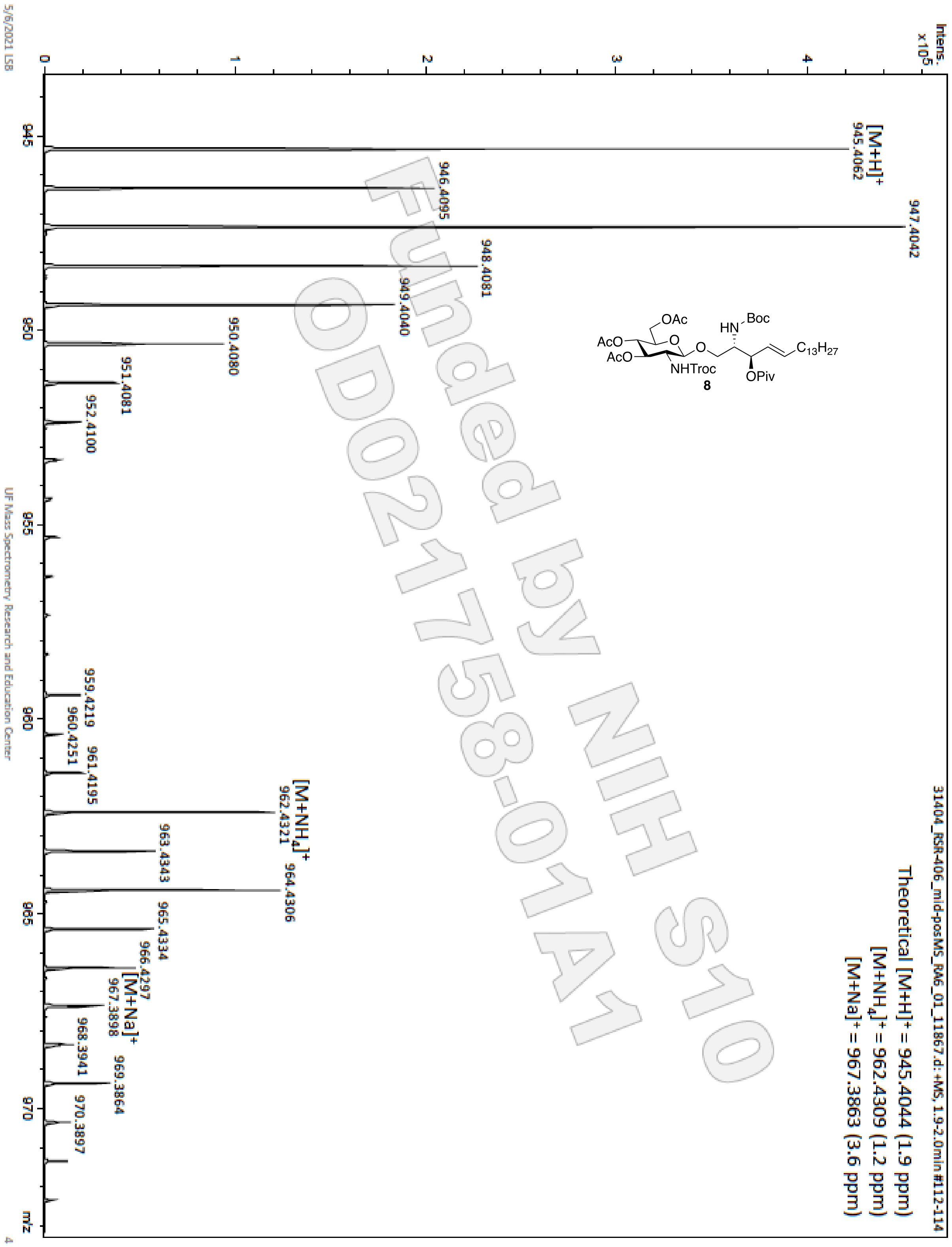

Figure 22: HRMS ESI-TOF of compound **8**.

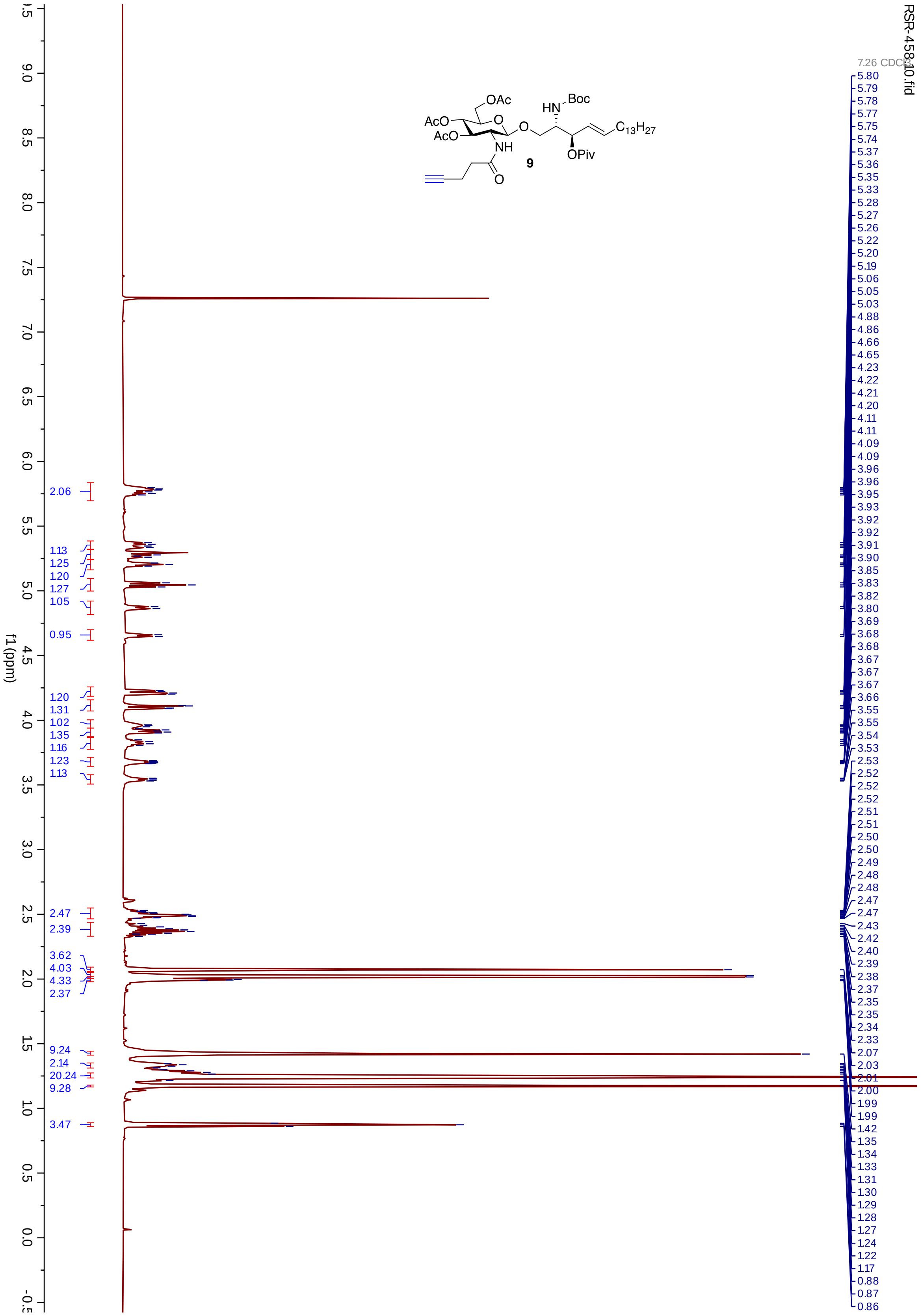

Figure 23: ^{1}H NMR of compound **9** (600 MHz, $CDCl_3$).

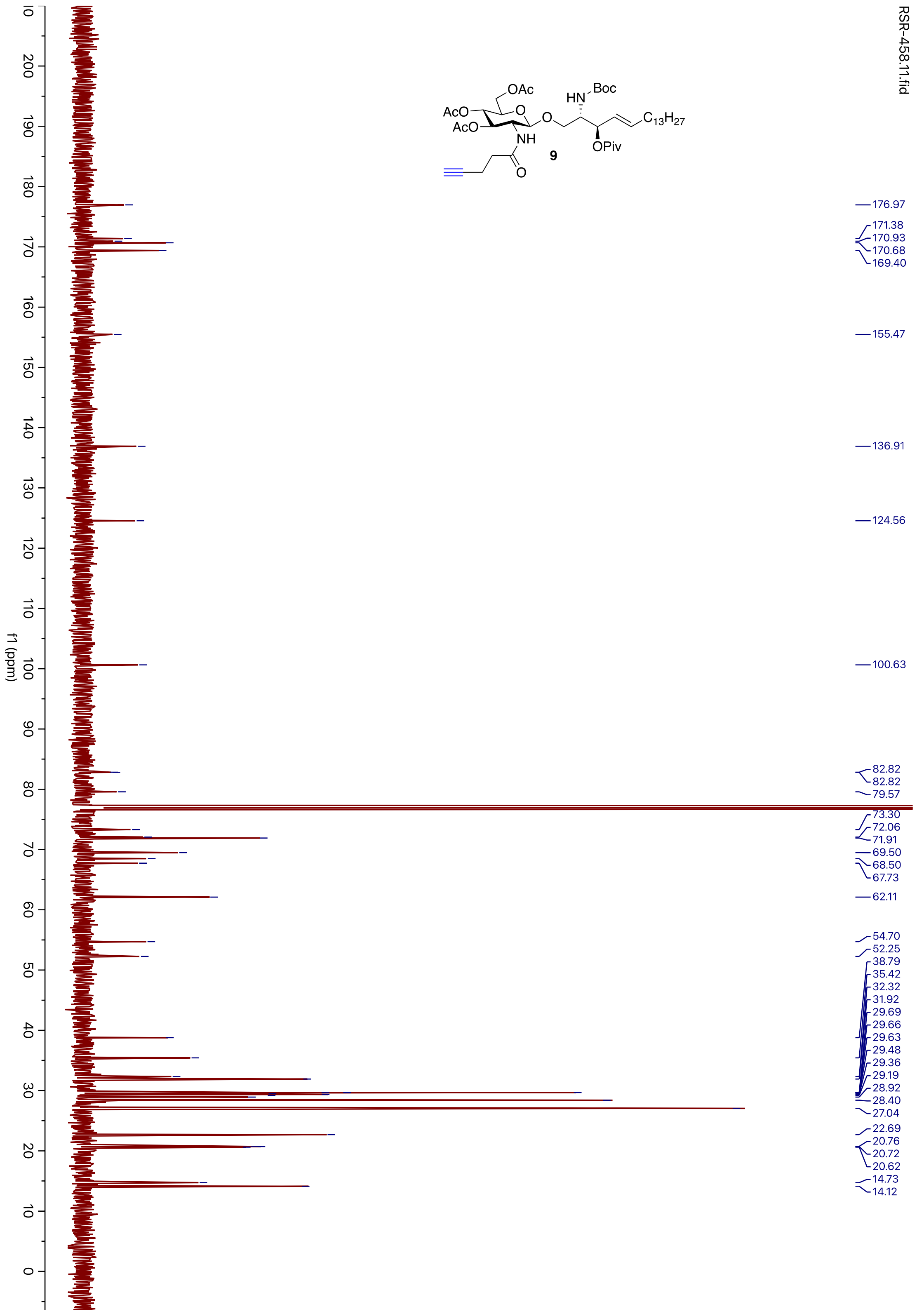

Figure 24: $^{13}C\{^{1}H\}$ NMR of compound **9** (150 MHz, $CDCl_3$).

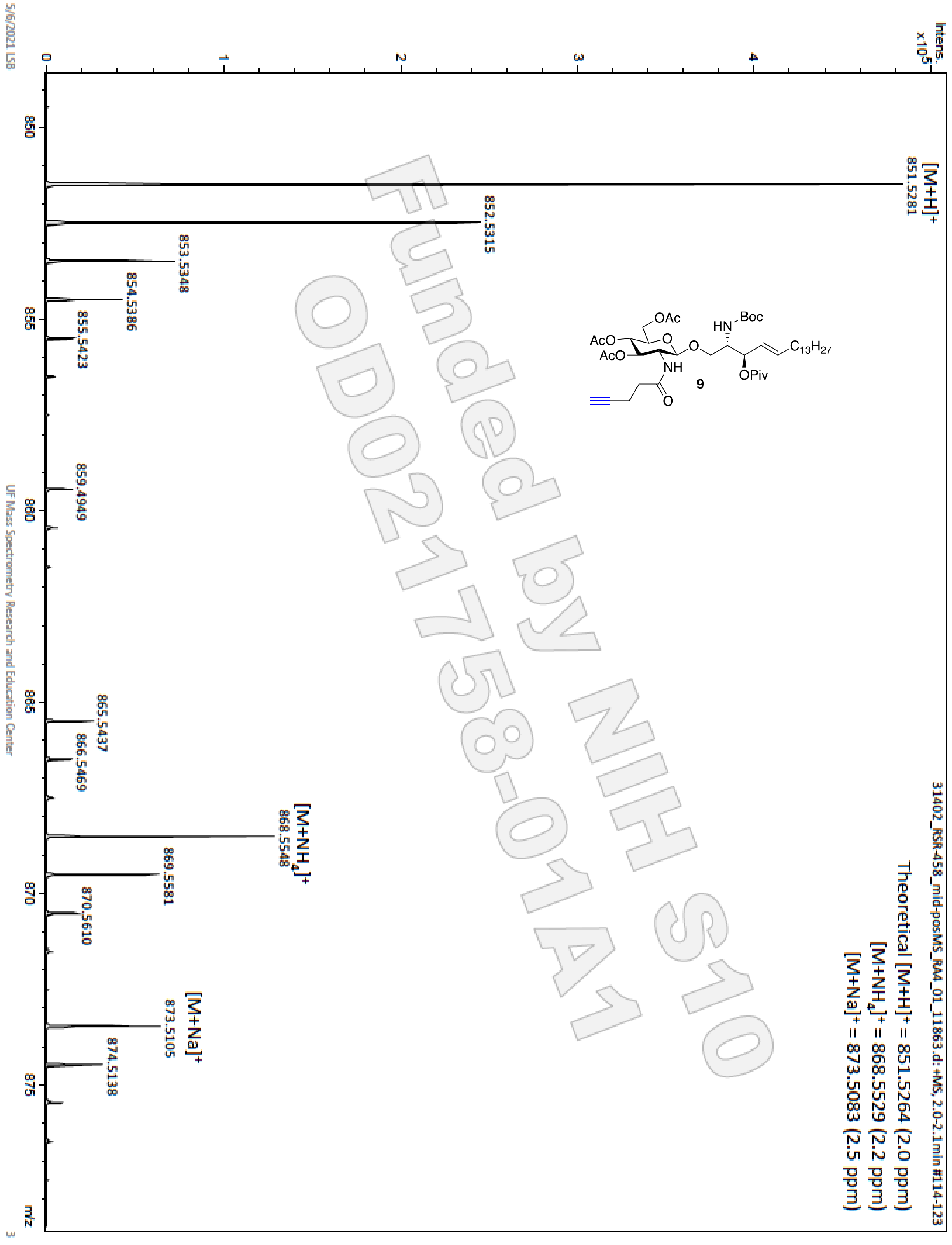

Figure 25: HRMS ESI-TOF of compound **9**.

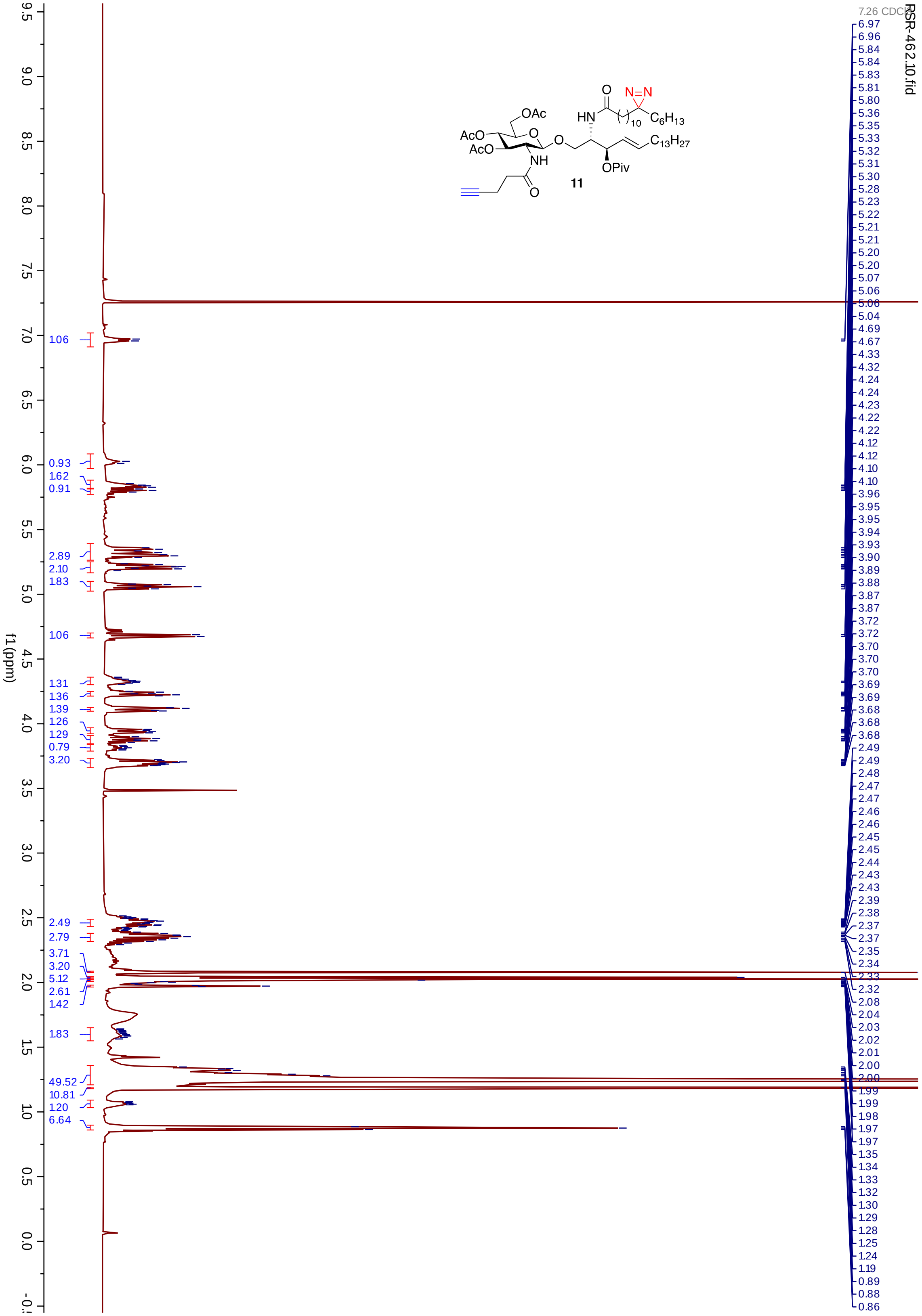

Figure 26: ^{1}H NMR of compound **11** (600 MHz, $CDCl_3$).

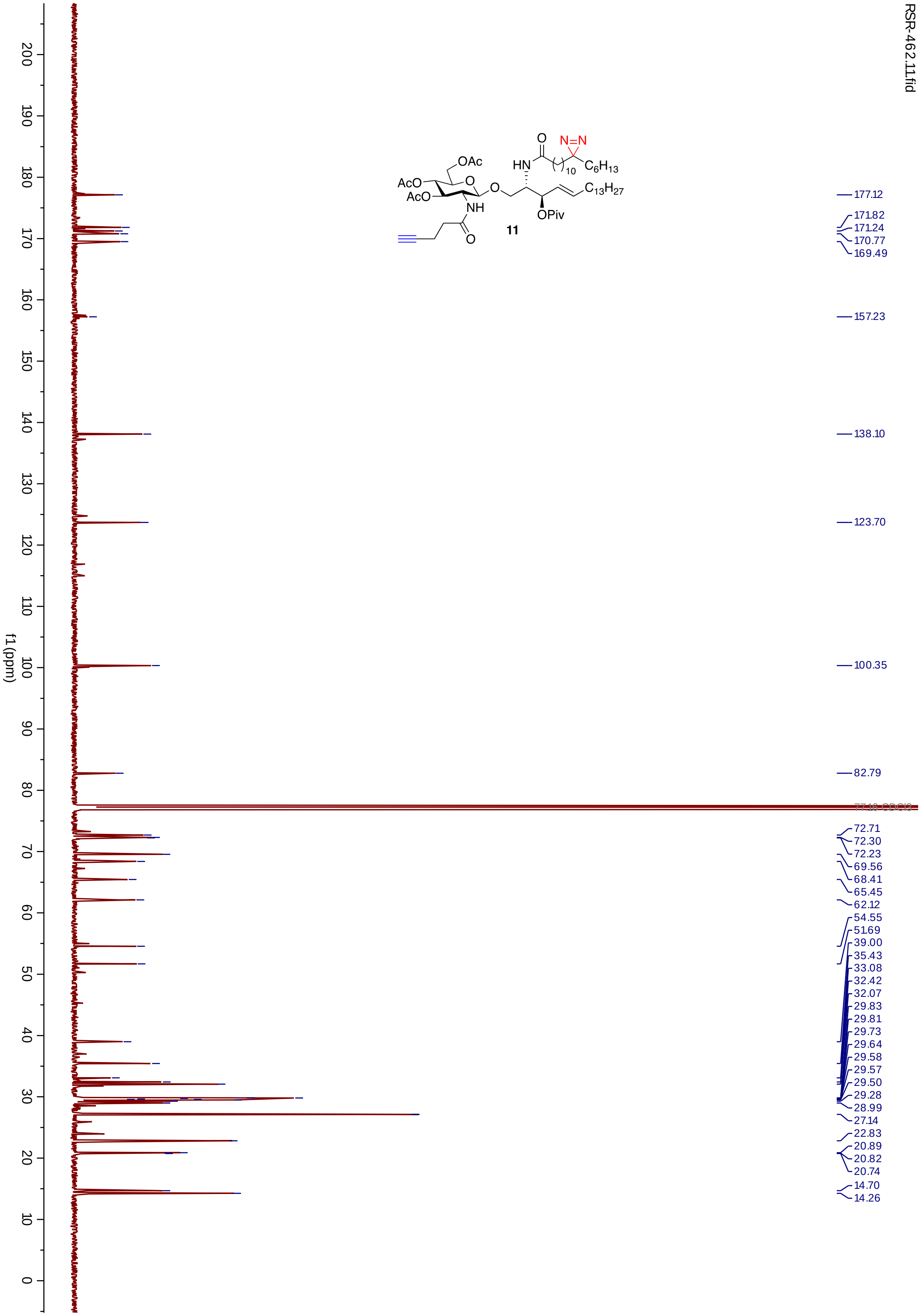

Figure 27: $^{13}C\{^1H\}$ NMR of compound **11** (150 MHz, $CDCl_3$).

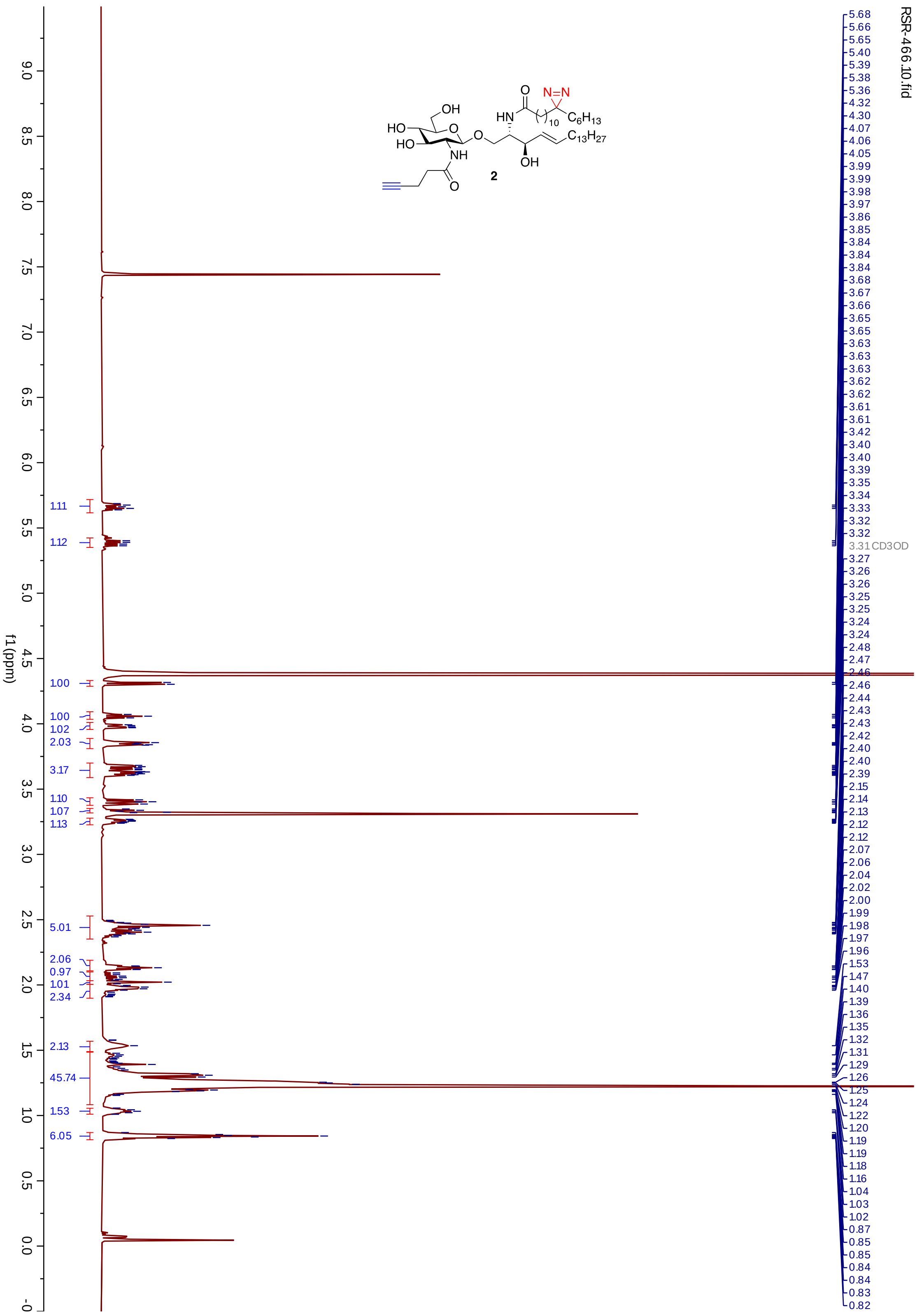

Figure 28: ^{1}H NMR of compound **2** (600 MHz, MeOD:CDCl$_3$ 1:3).

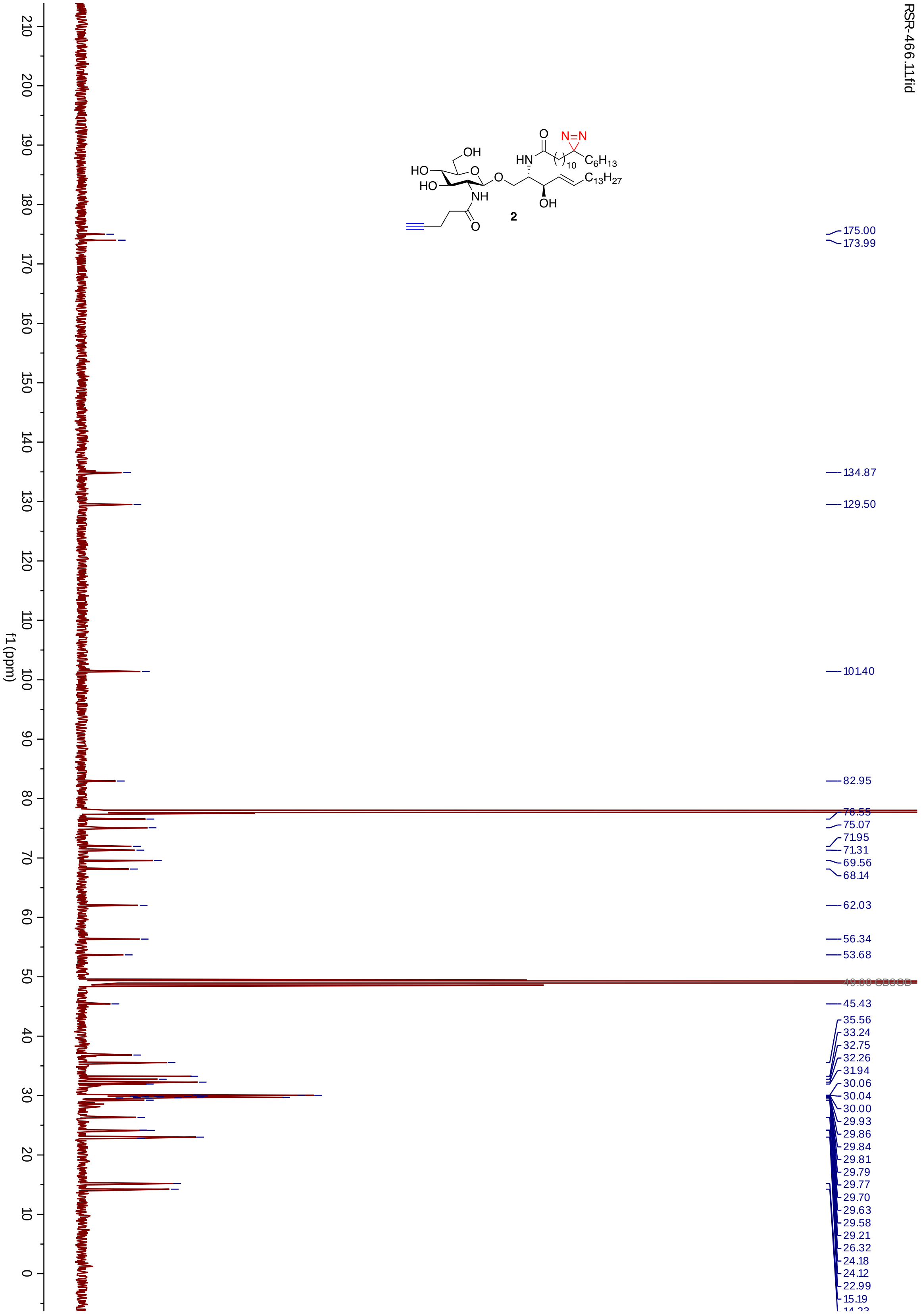

Figure 29: $^{13}C\{^{1}H\}$ NMR of compound **2** (150 MHz, MeOD:$CDCl_3$ 1:3).

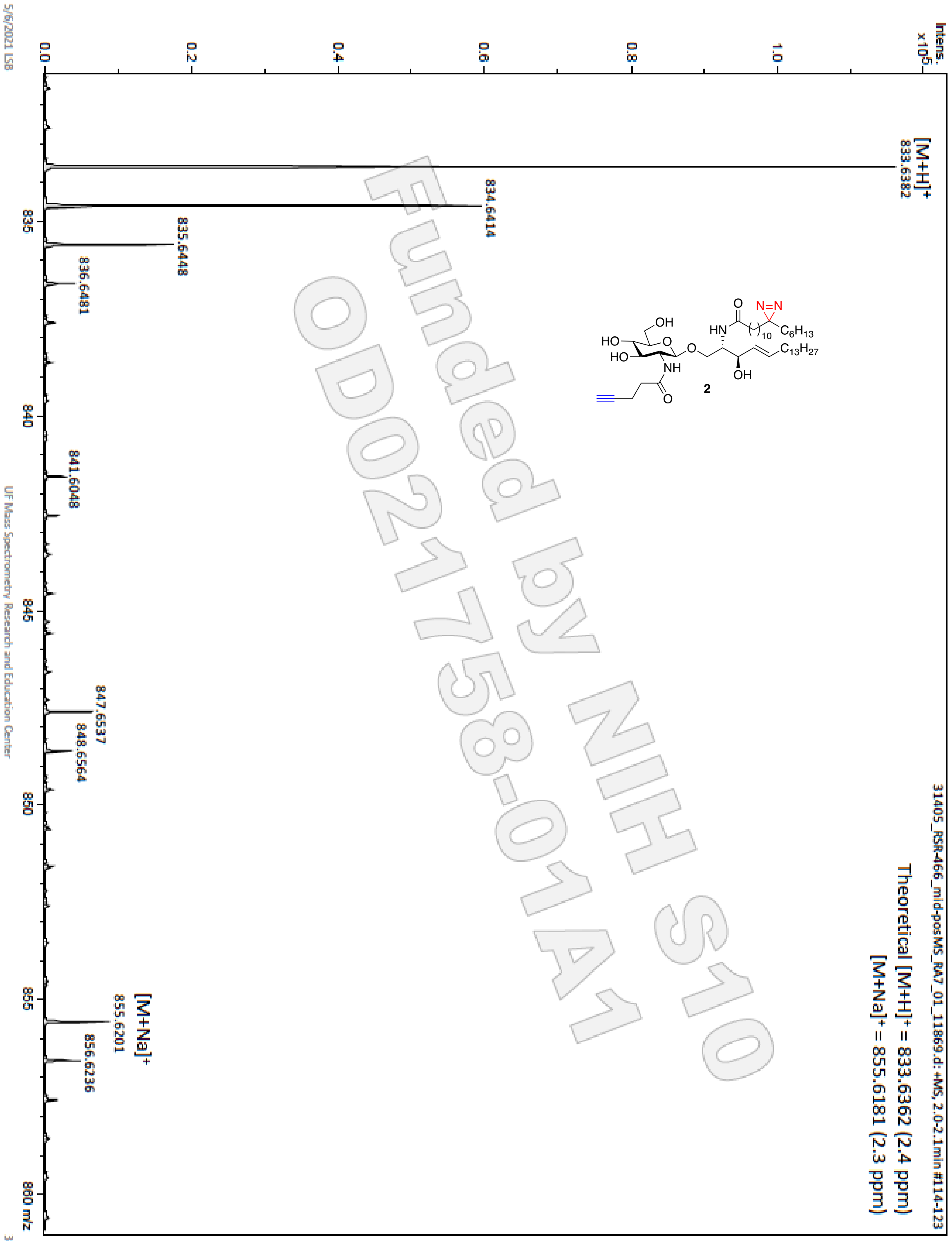

Figure 30: HRMS ESI-TOF of compound **2.**

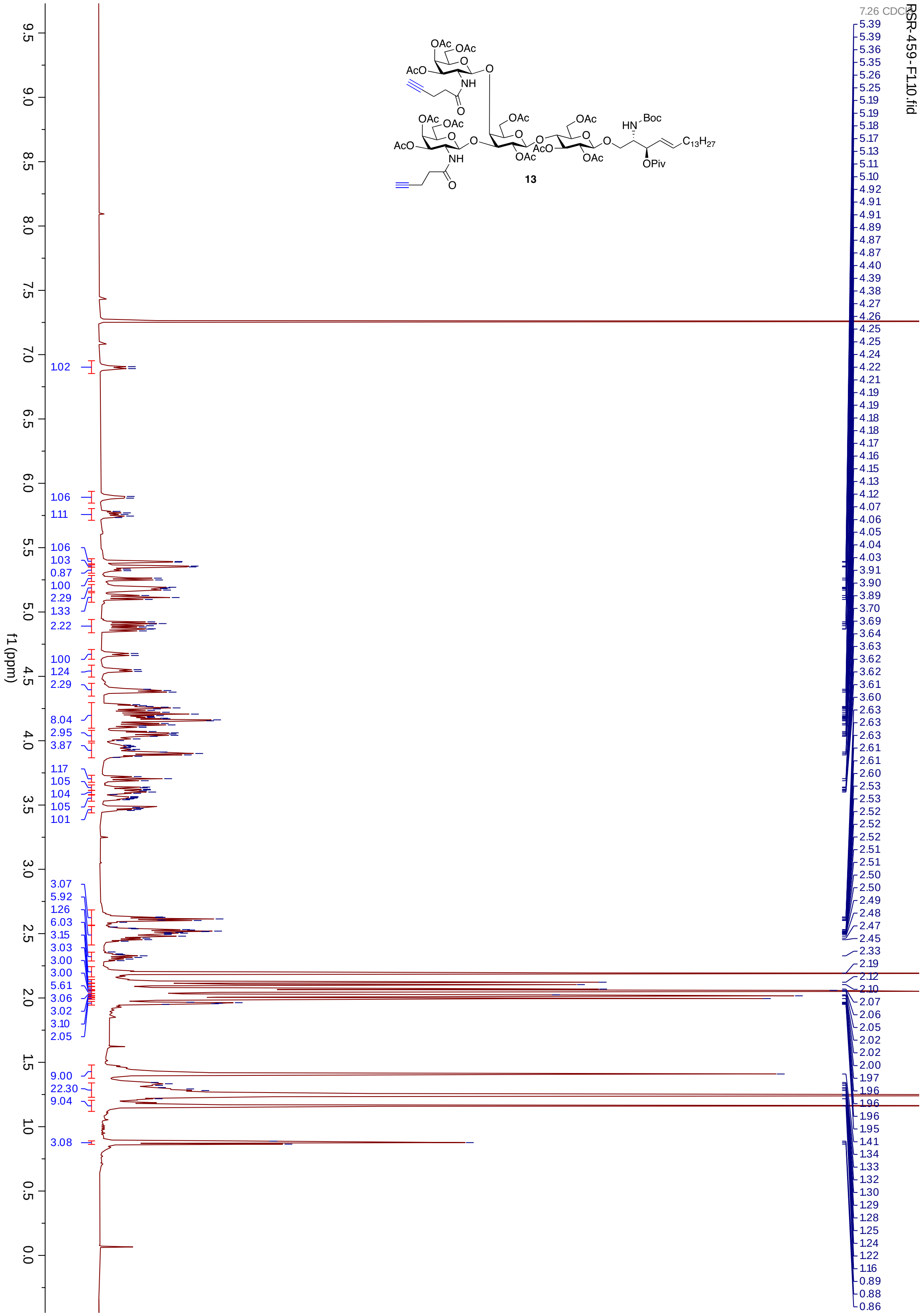

Figure 31: ^{1}H NMR of compound **13** (600 MHz, $CDCl_3$).

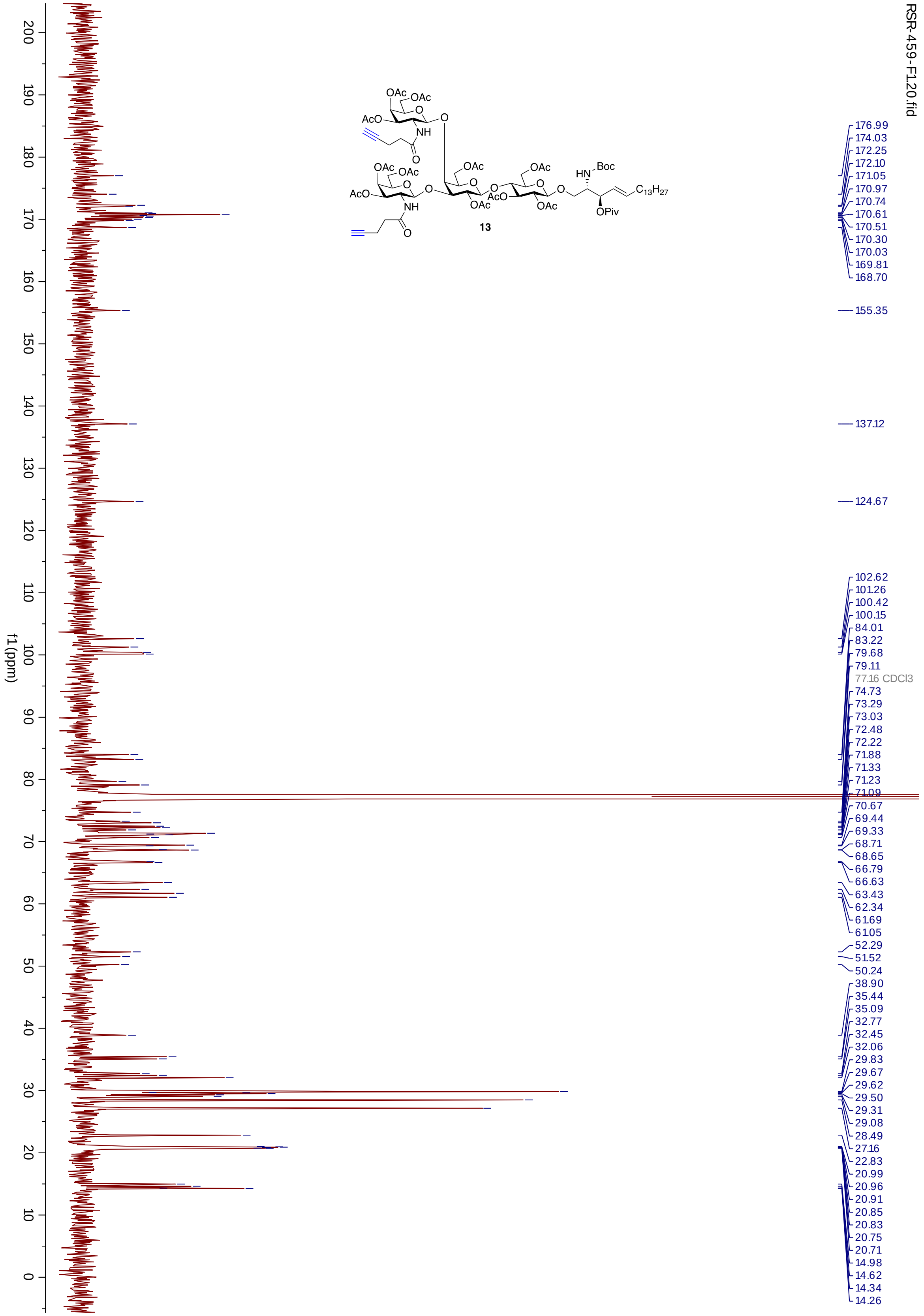

Figure 32: $^{13}C\{^{1}H\}$ NMR of compound **13** (150 MHz, $CDCl_3$).

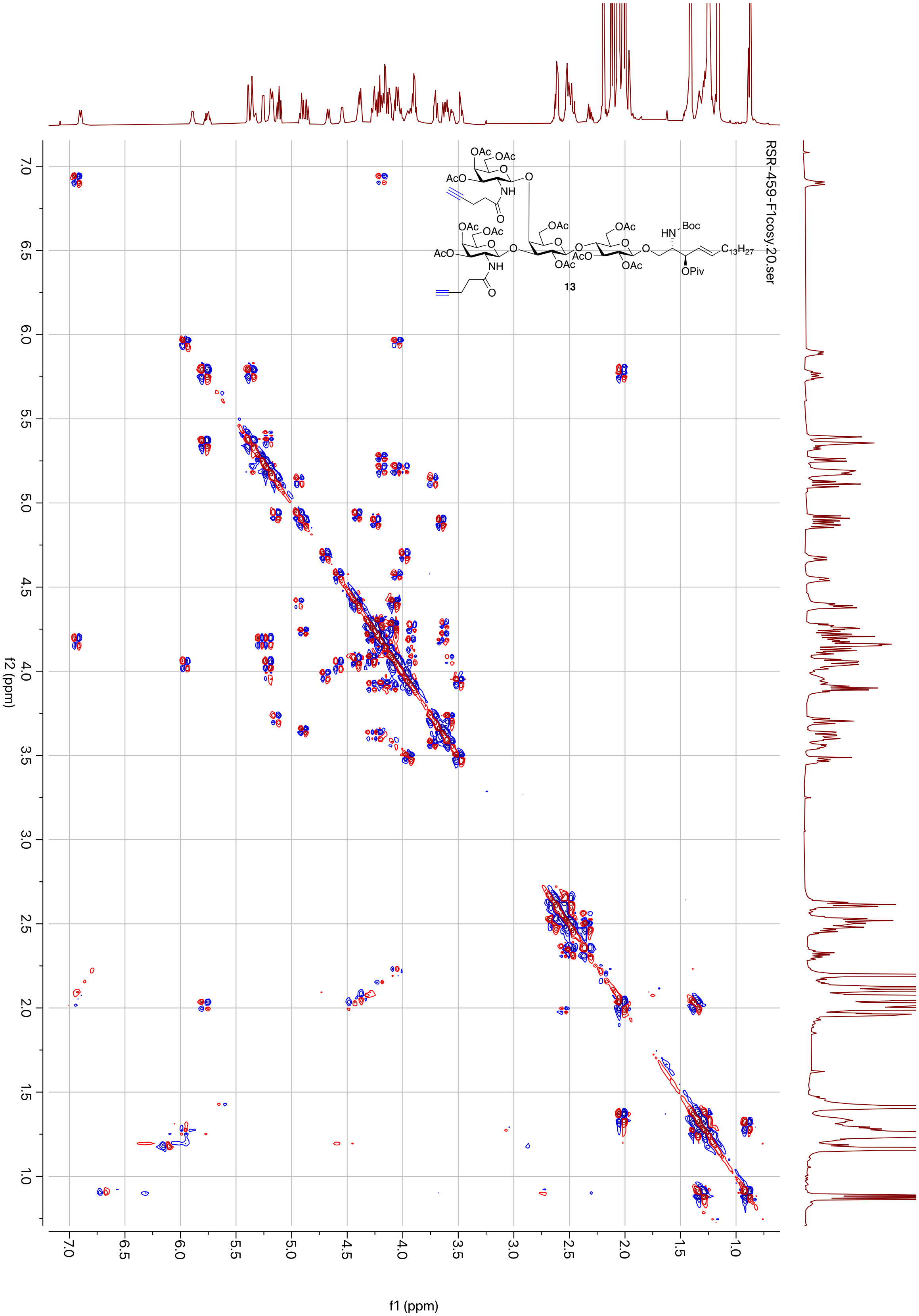

Figure 33. ^{1}H-^{1}H COSY NMR of compound **13** (600 MHz, $CDCl_3$).

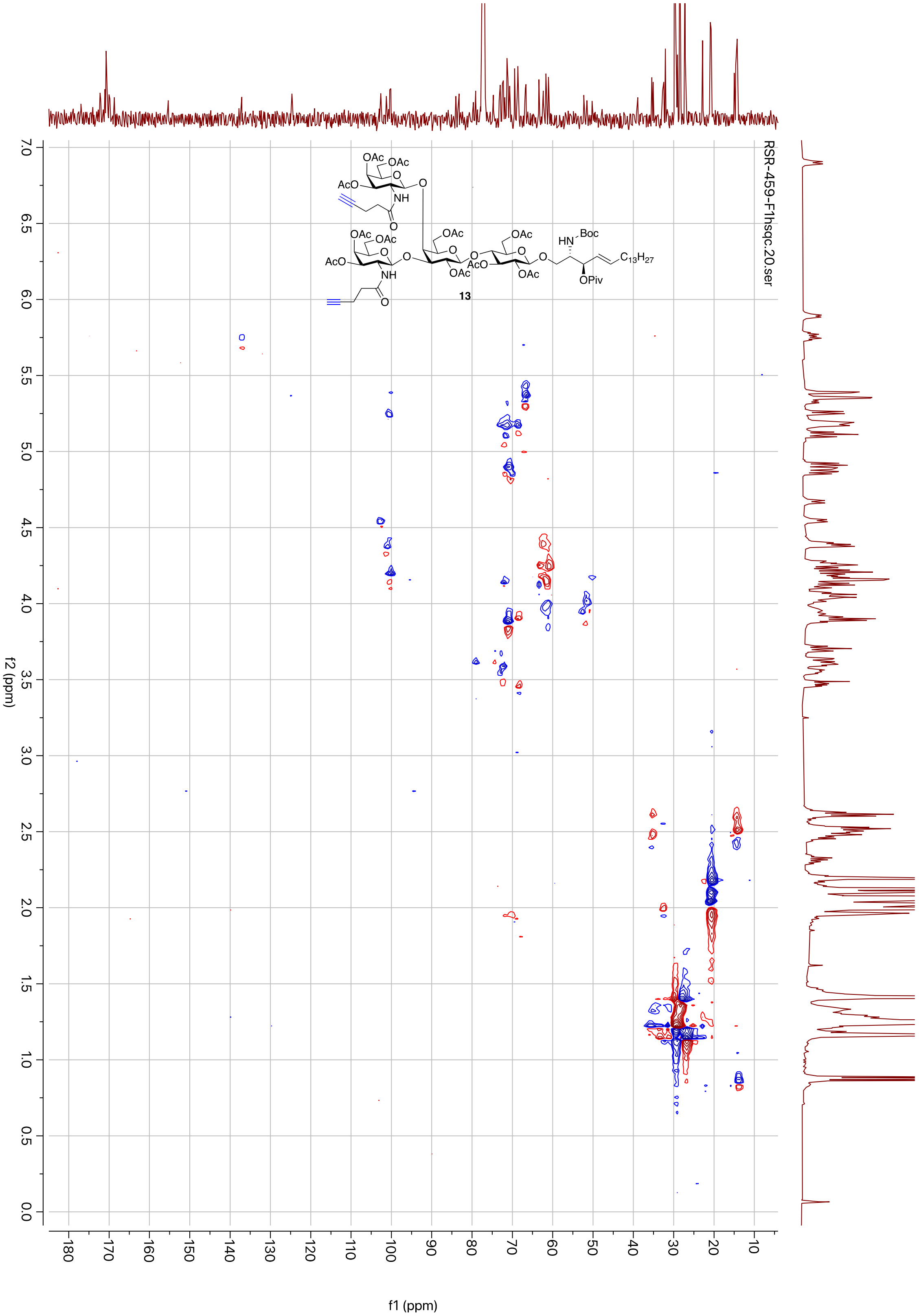

Figure 34. ^{1}H-^{13}C HSQC NMR of compound **13** (600/150 MHz, $CDCl_3$).

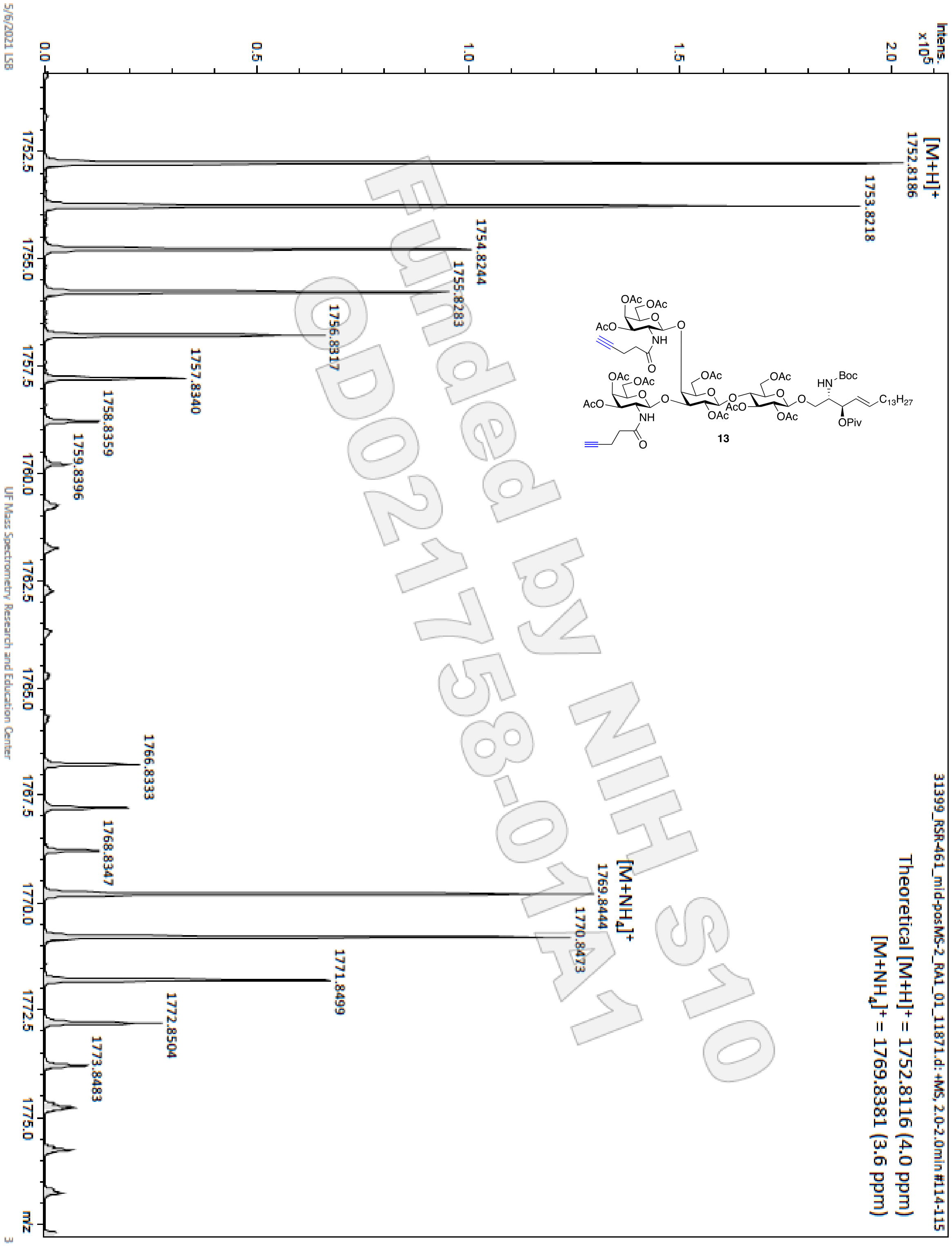

Figure 35: HRMS ESI-TOF of compound **13**.

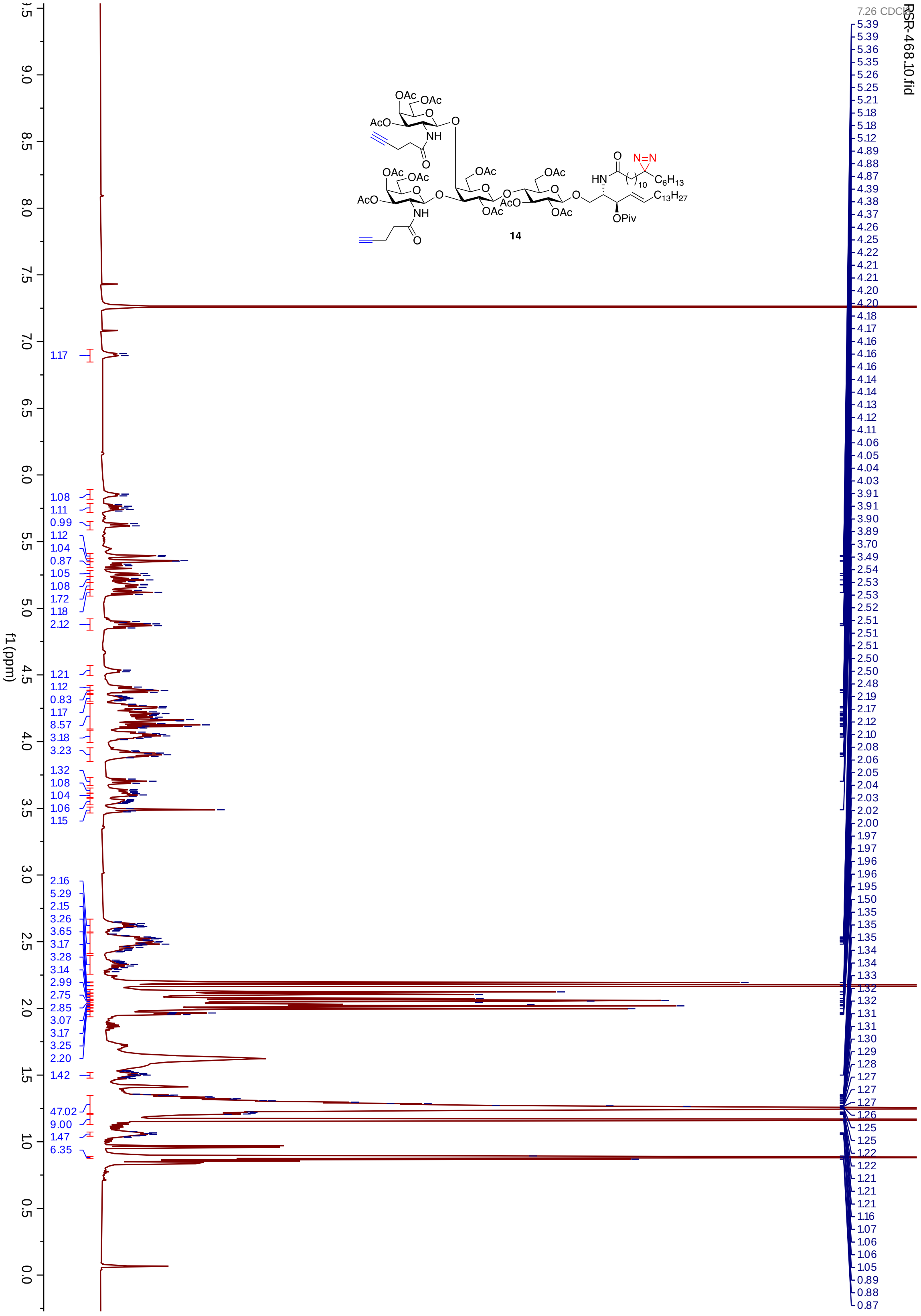

Figure 36: ^{1}H NMR of compound **14** (600 MHz, $CDCl_3$).

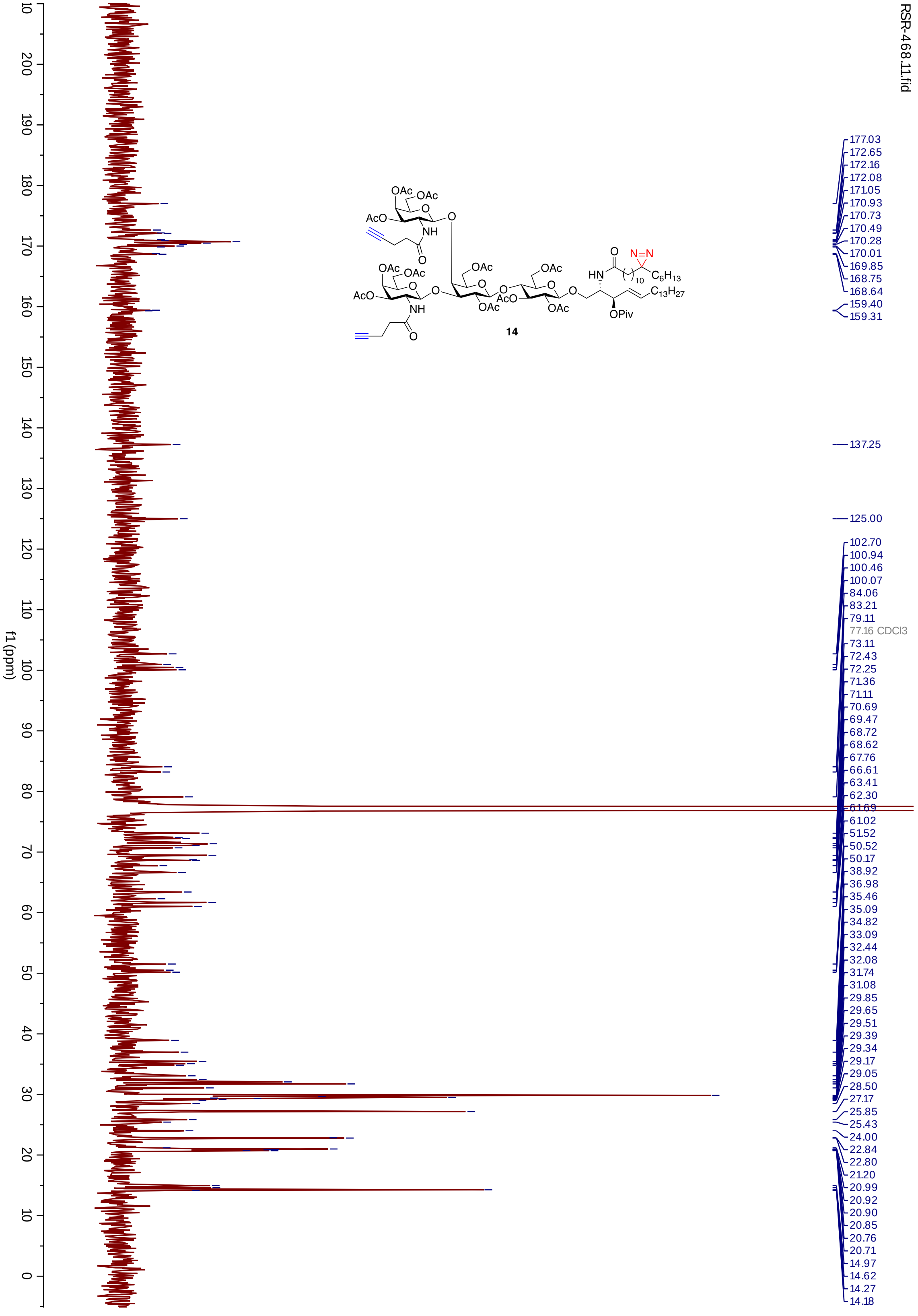

Figure 37: $^{13}C\{^{1}H\}$ NMR of compound **14** (150 MHz, $CDCl_3$).

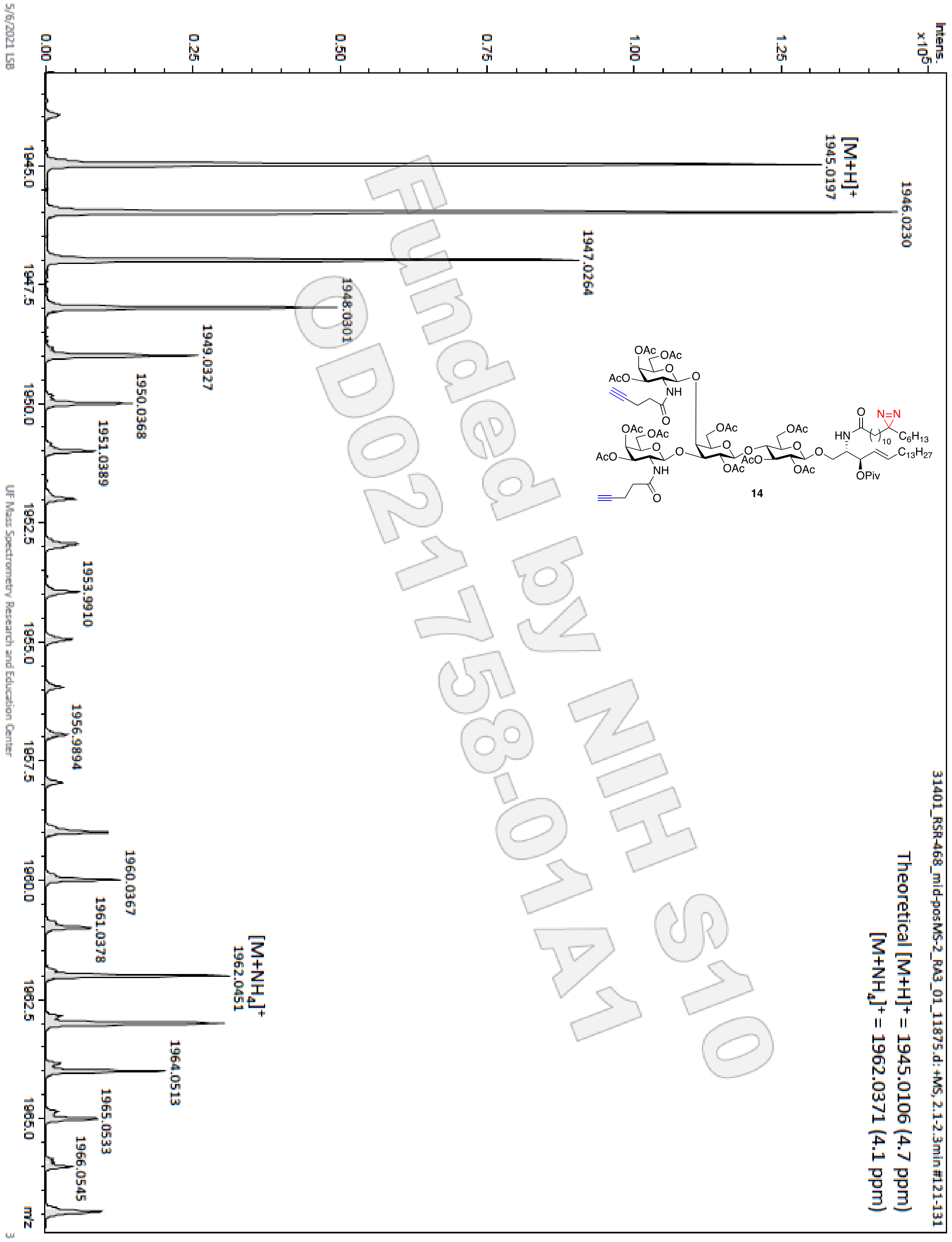

Funded by NIH S10 OD021758-01A1

Figure 38: HRMS ESI-TOF of compound **14**.

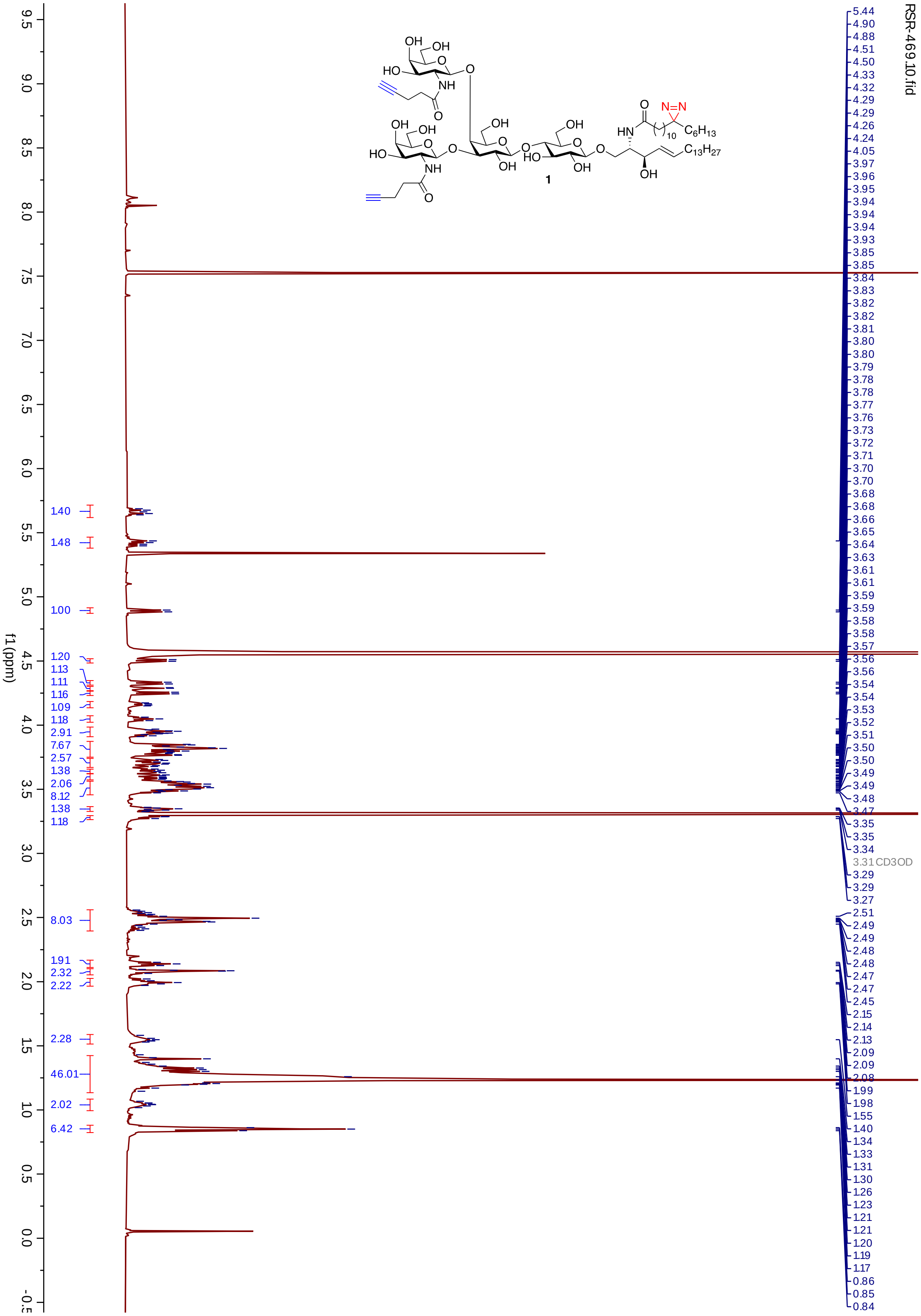

Figure 39: ^{1}H NMR of compound **1** (600 MHz, MeOD:$CDCl_3$ 1:1).

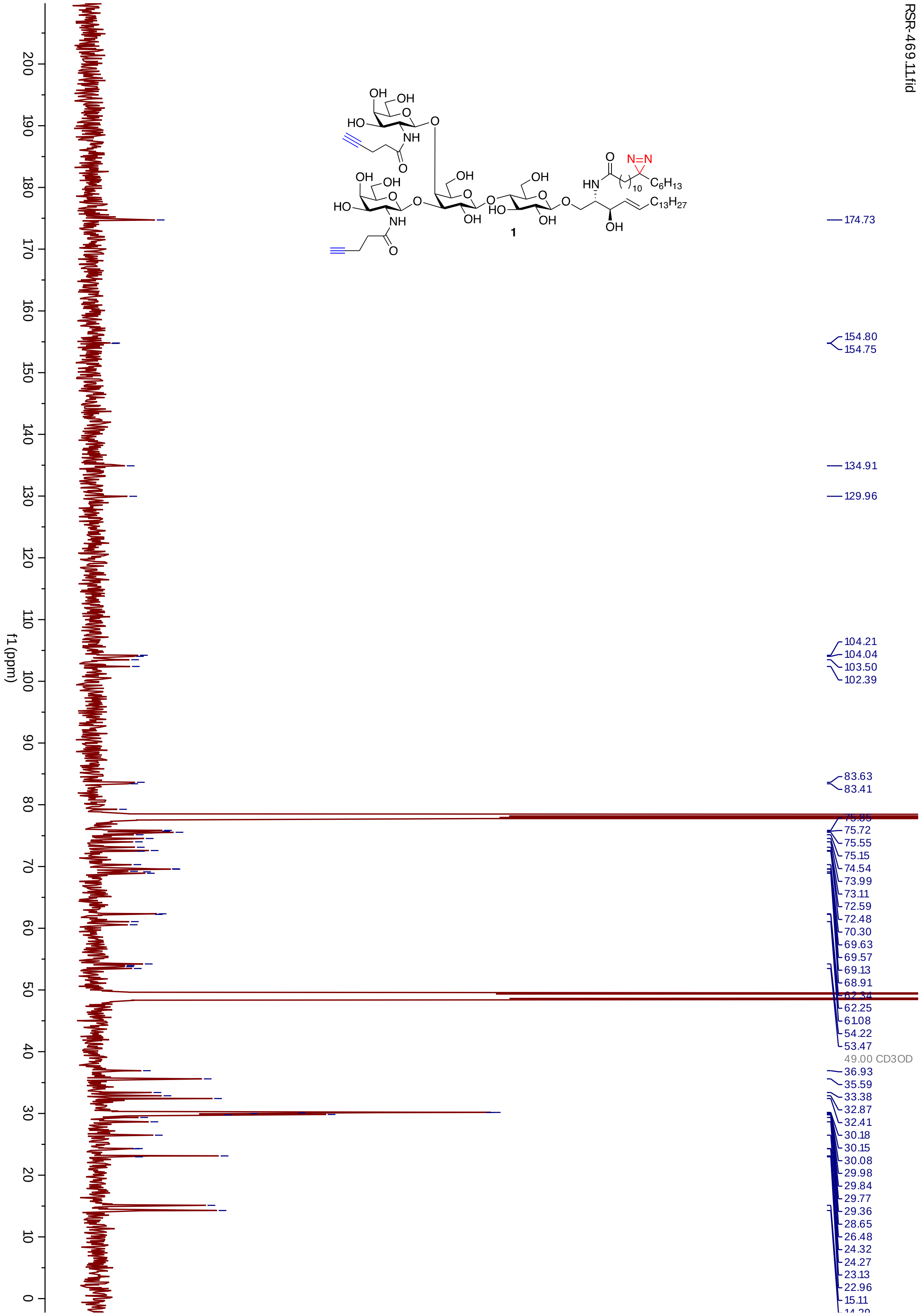

Figure 40: $^{13}C\{^{1}H\}$ NMR of compound **1** (150 MHz, MeOD:$CDCl_3$ 1:1).

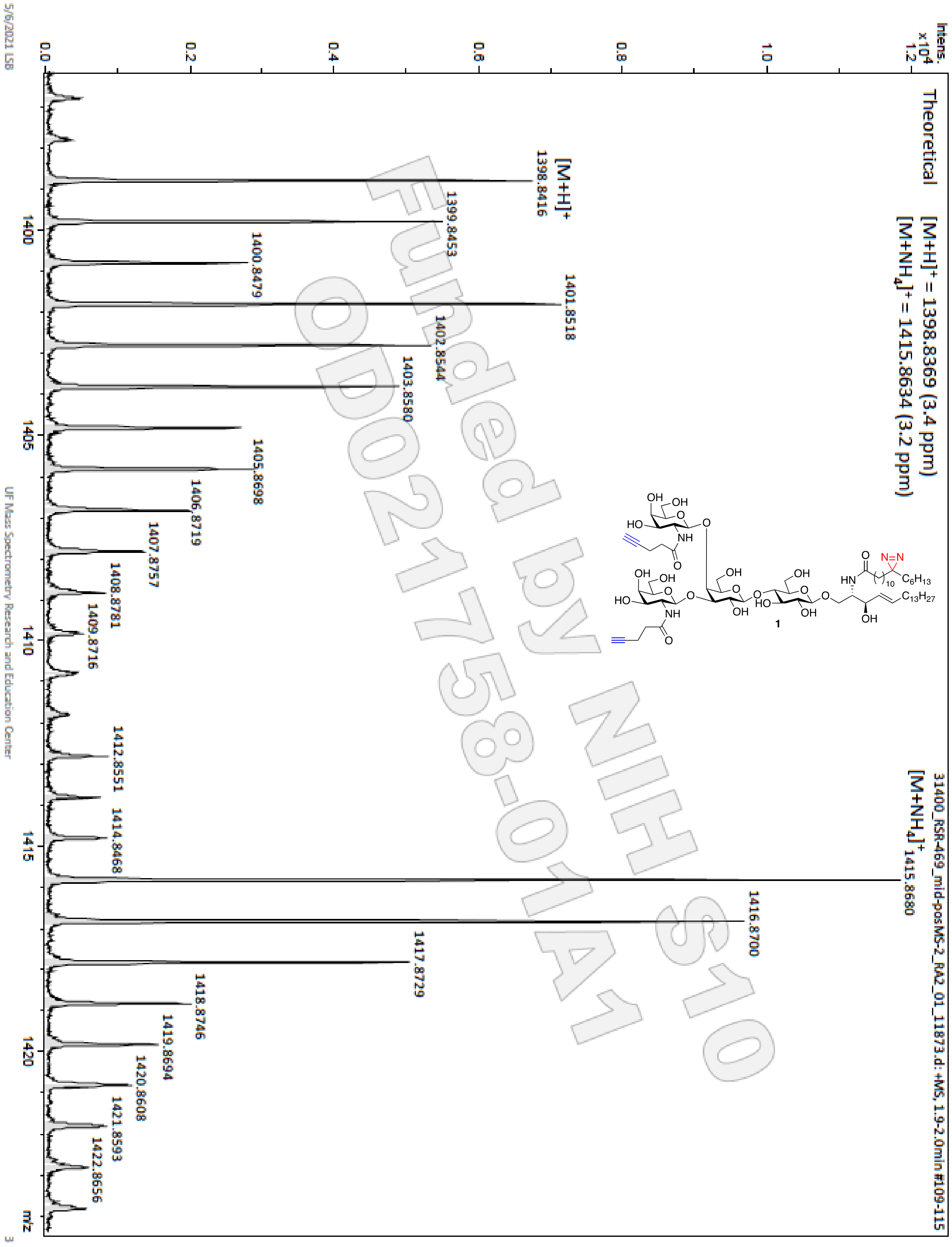

Figure 41: HRMS ESI-TOF of compound **1**.

References:

1. Kim, J. H.; Yang, C. K.; Heo, K.; Roeder, R. G.; An, W.; Stallcup, M. R., CCAR1, a key regulator of mediator complex recruitment to nuclear receptor transcription complexes. *Mol Cell* 2008, *31* (4), 510-9.
2. Consortium, U., UniProt: the universal protein knowledgebase in 2021. *Nucleic Acids Res* 2021, *49* (D1), D480-9.
3. Möller, I.; Beatrix, B.; Kreibich, G.; Sakai, H.; Lauring, B.; Wiedmann, M., Unregulated exposure of the ribosomal M-site caused by NAC depletion results in delivery of non-secretory polypeptides to the Sec61 complex. *FEBS Lett* 1998, *441* (1), 1-5.
4. Yotov, W. V.; St-Arnaud, R., Differential splicing-in of a proline-rich exon converts alphaNAC into a muscle-specific transcription factor. *Genes Dev* 1996, *10* (14), 1763-72.
5. Kovtun, O.; Tillu, V. A.; Ariotti, N.; Parton, R. G.; Collins, B. M., Cavin family proteins and the assembly of caveolae. *J Cell Sci* 2015, *128* (7), 1269-78.
6. Syrovatkina, V.; Alegre, K. O.; Dey, R.; Huang, X. Y., Regulation, Signaling, and Physiological Functions of G-Proteins. *J Mol Biol* 2016, *428* (19), 3850-68.
7. Liu, C. C.; Liu, Y. Y.; Zhou, J. F.; Chen, X.; Chen, H.; Hu, J. H.; Chen, J.; Zhang, J.; Sun, R. C.; Wei, J. C.; Go, Y. Y.; Morita, E.; Zhou, B., Cellular ESCRT components are recruited to regulate the endocytic trafficking and RNA replication compartment assembly during classical swine fever virus infection. *PLoS Pathog* 2022, *18* (2), e1010294.
8. Kabe, Y.; Goto, M.; Shima, D.; Imai, T.; Wada, T.; Morohashi, K.; Shirakawa, M.; Hirose, S.; Handa, H., The role of human MBF1 as a transcriptional coactivator. *J Biol Chem* 1999, *274* (48), 34196-202.
9. Prieto-Sánchez, R. M.; Berenjeno, I. M.; Bustelo, X. R., Involvement of the Rho/Rac family member RhoG in caveolar endocytosis. *Oncogene* 2006, *25* (21), 2961-73.
10. Zou, Q.; Qi, H., Deletion of ribosomal paralogs Rpl39 and Rpl39l compromises cell proliferation via protein synthesis and mitochondrial activity. *Int J Biochem Cell Biol* 2021, *139*, 106070.
11. Hubbard, C.; Singleton, D.; Rauch, M.; Jayasinghe, S.; Cafiso, D.; Castle, D., The secretory carrier membrane protein family: structure and membrane topology. *Mol Biol Cell* 2000, *11* (9), 2933-47.
12. Hata, S.; Sorimachi, H.; Nakagawa, K.; Maeda, T.; Abe, K.; Suzuki, K., Domain II of m-calpain is a Ca(2+)-dependent cysteine protease. *FEBS Lett* 2001, *501* (2-3), 111-4.
13. Merino-Trigo, A.; Kerr, M. C.; Houghton, F.; Lindberg, A.; Mitchell, C.; Teasdale, R. D.; Gleeson, P. A., Sorting nexin 5 is localized to a subdomain of the early endosomes and is recruited to the plasma membrane following EGF stimulation. *J Cell Sci* 2004, *117* (Pt 26), 6413-24.
14. Sampson, J.; Richards, M. W.; Choi, J.; Fry, A. M.; Bayliss, R., Phase-separated foci of EML4-ALK facilitate signalling and depend upon an active kinase conformation. *EMBO Rep* 2021, *22* (12), e53693.
15. Gustafsson Sheppard, N.; Jarl, L.; Mahadessian, D.; Strittmatter, L.; Schmidt, A.; Madhusudan, N.; Tegnér, J.; Lundberg, E. K.; Asplund, A.; Jain, M.; Nilsson, R., The folate-coupled enzyme MTHFD2 is a nuclear protein and promotes cell proliferation. *Sci Rep* 2015, *5*, 15029.
16. Espinha, G.; Osaki, J. H.; Magalhaes, Y. T.; Forti, F. L., Rac1 GTPase-deficient HeLa cells present reduced DNA repair, proliferation, and survival under UV or gamma irradiation. *Mol Cell Biochem* 2015, *404* (1-2), 281-97.

17. Illigmann, A.; Thoma, Y.; Pan, S.; Reinhardt, L.; Brötz-Oesterhelt, H., Contribution of the Clp Protease to Bacterial Survival and Mitochondrial Homoeostasis. *Microb Physiol* 2021, *31* (3), 260-79.
18. Coelho, A. R.; Oliveira, P. J., Dihydroorotate dehydrogenase inhibitors in SARS-CoV-2 infection. *Eur J Clin Invest* 2020, *50* (10), e13366.
19. Conti, M., Phosphodiesterases and cyclic nucleotide signaling in endocrine cells. *Mol Endocrinol* 2000, *14* (9), 1317-27.
20. Berger, K.; Lindh, R.; Wierup, N.; Zmuda-Trzebiatowska, E.; Lindqvist, A.; Manganiello, V. C.; Degerman, E., Phosphodiesterase 3B is localized in caveolae and smooth ER in mouse hepatocytes and is important in the regulation of glucose and lipid metabolism. *PLoS One* 2009, *4* (3), e4671.
21. Tulin, E. E.; Onoda, N.; Hasegawa, M.; Nosaka, T.; Nomura, H.; Kitamura, T., Genetic approach and phenotype-based complementation screening for identification of stroma cell-derived proteins involved in cell proliferation. *Exp Cell Res* 2002, *272* (1), 23-31.
22. Donaldson, J. G.; Jackson, C. L., ARF family G proteins and their regulators: roles in membrane transport, development and disease. *Nat Rev Mol Cell Biol* 2011, *12* (6), 362-75.
23. Sato, M.; Sato, K.; Fonarev, P.; Huang, C. J.; Liou, W.; Grant, B. D., Caenorhabditis elegans RME-6 is a novel regulator of RAB-5 at the clathrin-coated pit. *Nat Cell Biol* 2005, *7* (6), 559-69.
24. Renard, H. F.; Tyckaert, F.; Lo Giudice, C.; Hirsch, T.; Valades-Cruz, C. A.; Lemaigre, C.; Shafaq-Zadah, M.; Wunder, C.; Wattiez, R.; Johannes, L.; van der Bruggen, P.; Alsteens, D.; Morsomme, P., Endophilin-A3 and Galectin-8 control the clathrin-independent endocytosis of CD166. *Nat Commun* 2020, *11* (1), 1457.
25. Vainberg, I. E.; Lewis, S. A.; Rommelaere, H.; Ampe, C.; Vandekerckhove, J.; Klein, H. L.; Cowan, N. J., Prefoldin, a chaperone that delivers unfolded proteins to cytosolic chaperonin. *Cell* 1998, *93* (5), 863-73.
26. Cárcel-Trullols, J.; Kovács, A. D.; Pearce, D. A., Cell biology of the NCL proteins: What they do and don't do. *Biochim Biophys Acta* 2015, *1852* (10 Pt B), 2242-55.
27. Fujisawa, K.; Terai, S.; Takami, T.; Yamamoto, N.; Yamasaki, T.; Matsumoto, T.; Yamaguchi, K.; Owada, Y.; Nishina, H.; Noma, T.; Sakaida, I., Modulation of anti-cancer drug sensitivity through the regulation of mitochondrial activity by adenylate kinase 4. *J Exp Clin Cancer Res* 2016, *35*, 48.
28. Rojas, A. M.; Fuentes, G.; Rausell, A.; Valencia, A., The Ras protein superfamily: evolutionary tree and role of conserved amino acids. *J Cell Biol* 2012, *196* (2), 189-201.
29. Lata, S.; Schoehn, G.; Jain, A.; Pires, R.; Piehler, J.; Gottlinger, H. G.; Weissenhorn, W., Helical structures of ESCRT-III are disassembled by VPS4. *Science* 2008, *321* (5894), 1354-7.
30. Dermody, J. L.; Buratowski, S., Leo1 subunit of the yeast paf1 complex binds RNA and contributes to complex recruitment. *J Biol Chem* 2010, *285* (44), 33671-9.
31. Angrisani, A.; Di Fiore, A.; De Smaele, E.; Moretti, M., The emerging role of the KCTD proteins in cancer. *Cell Commun Signal* 2021, *19* (1), 56.
32. Gundelfinger, E. D.; Reissner, C.; Garner, C. C., Role of Bassoon and Piccolo in Assembly and Molecular Organization of the Active Zone. *Front Synaptic Neurosci* 2015, *7*, 19.
33. Magaña-Acosta, M.; Valadez-Graham, V., Chromatin Remodelers in the 3D Nuclear Compartment. *Front Genet* 2020, *11*, 600615.

34. Zhan, X.; Yan, C.; Zhang, X.; Lei, J.; Shi, Y., Structure of a human catalytic step I spliceosome. *Science* 2018, *359* (6375), 537-45.
35. Cai, M.; Li, H.; Chen, R.; Zhou, X., MRPL13 Promotes Tumor Cell Proliferation, Migration and EMT Process in Breast Cancer Through the PI3K-AKT-mTOR Pathway. *Cancer Manag Res* 2021, *13*, 2009-24.
36. Esposti, M. D., The roles of Bid. *Apoptosis* 2002, *7* (5), 433-40.
37. Takagi, M.; Sueishi, M.; Saiwaki, T.; Kametaka, A.; Yoneda, Y., A novel nucleolar protein, NIFK, interacts with the forkhead associated domain of Ki-67 antigen in mitosis. *J Biol Chem* 2001, *276* (27), 25386-91.
38. Li, H.; Byeon, I. J.; Ju, Y.; Tsai, M. D., Structure of human Ki67 FHA domain and its binding to a phosphoprotein fragment from hNIFK reveal unique recognition sites and new views to the structural basis of FHA domain functions. *J Mol Biol* 2004, *335* (1), 371-81.
39. Schapira, M.; Tyers, M.; Torrent, M.; Arrowsmith, C. H., WD40 repeat domain proteins: a novel target class? *Nat Rev Drug Discov* 2017, *16* (11), 773-86.
40. Fraga de Andrade, I.; Mehta, C.; Bresnick, E. H., Post-transcriptional control of cellular differentiation by the RNA exosome complex. *Nucleic Acids Res* 2020, *48* (21), 11913-28.
41. Ramalho-Oliveira, R.; Oliveira-Vieira, B.; Viola, J. P. B., IRF2BP2: A new player in the regulation of cell homeostasis. *J Leukoc Biol* 2019, *106* (3), 717-23.
42. Xu, F.; Du, W.; Zou, Q.; Wang, Y.; Zhang, X.; Xing, X.; Li, Y.; Zhang, D.; Wang, H.; Zhang, W.; Hu, X.; Liu, X.; Zhang, S.; Yu, J.; Fang, J.; Li, F.; Zhou, Y.; Yue, T.; Mi, N.; Deng, H.; Zou, P.; Chen, X.; Yang, X.; Yu, L., COPII mitigates ER stress by promoting formation of ER whorls. *Cell Res* 2021, *31* (2), 141-56.
43. Rao, J.; Wu, X.; Zhou, X.; Deng, R.; Ma, Y., TMEM205 Is an Independent Prognostic Factor and Is Associated With Immune Cell Infiltrates in Hepatocellular Carcinoma. *Front Genet* 2020, *11*, 575776.
44. Abella, J. V.; Galloni, C.; Pernier, J.; Barry, D. J.; Kjær, S.; Carlier, M. F.; Way, M., Isoform diversity in the Arp2/3 complex determines actin filament dynamics. *Nat Cell Biol* 2016, *18* (1), 76-86.
45. Takahashi, M.; Kobayashi, T., Cholesterol regulation of rab-mediated sphingolipid endocytosis. *Glycoconj J* 2009, *26* (6), 705-10.
46. Marks, D. L.; Pagano, R. E., Endocytosis and sorting of glycosphingolipids in sphingolipid storage disease. *Trends Cell Biol* 2002, *12* (12), 605-13.
47. D'Souza-Schorey, C.; Chavrier, P., ARF proteins: roles in membrane traffic and beyond. *Nat Rev Mol Cell Biol* 2006, *7* (5), 347-58.
48. Geldner, N., The plant endosomal system--its structure and role in signal transduction and plant development. *Planta* 2004, *219* (4), 547-60.
49. Sivá, M.; Svoboda, M.; Veverka, V.; Trempe, J. F.; Hofmann, K.; Kožíšek, M.; Hexnerová, R.; Sedlák, F.; Belza, J.; Brynda, J.; Šácha, P.; Hubálek, M.; Starková, J.; Flaisigová, I.; Konvalinka, J.; Šašková, K. G., Human DNA-Damage-Inducible 2 Protein Is Structurally and Functionally Distinct from Its Yeast Ortholog. *Sci Rep* 2016, *6*, 30443.
50. Huang, S.; Dong, X.; Wang, J.; Ding, J.; Li, Y.; Li, D.; Lin, H.; Wang, W.; Zhao, M.; Chang, Q.; Zhou, N.; Cui, W.; Huang, C., Overexpression of the Ubiquilin-4 (UBQLN4) is Associated with Cell Cycle Arrest and Apoptosis in Human Normal Gastric Epithelial Cell Lines GES-1 Cells by Activation of the ERK Signaling Pathway. *Med Sci Monit* 2018, *24*, 3564-70.
51. Lee, J. H.; Jomaa, A.; Chung, S.; Hwang Fu, Y. H.; Qian, R.; Sun, X.; Hsieh, H. H.; Chandrasekar, S.; Bi, X.; Mattei, S.; Boehringer, D.; Weiss, S.; Ban, N.; Shan, S. O.,

Receptor compaction and GTPase rearrangement drive SRP-mediated cotranslational protein translocation into the ER. *Sci Adv* 2021, *7* (21), eabg0942.

52. Levine, A. J., p53, the cellular gatekeeper for growth and division. *Cell* 1997, *88* (3), 323-31.
53. Samper-Martín, B.; Sarrias, A.; Lázaro, B.; Pérez-Montero, M.; Rodríguez-Rodríguez, R.; Ribeiro, M. P. C.; Bañón, A.; Wolfgeher, D.; Jessen, H. J.; Alsina, B.; Clotet, J.; Kron, S. J.; Saiardi, A.; Jiménez, J.; Bru, S., Polyphosphate degradation by Nudt3-Zn. *Cell Rep* 2021, *37* (7), 110004.
54. Kavanagh, K. L.; Jörnvall, H.; Persson, B.; Oppermann, U., Medium- and short-chain dehydrogenase/reductase gene and protein families : the SDR superfamily: functional and structural diversity within a family of metabolic and regulatory enzymes. *Cell Mol Life Sci* 2008, *65* (24), 3895-906.
55. Maeda, Y.; Tanaka, S.; Hino, J.; Kangawa, K.; Kinoshita, T., Human dolichol-phosphate-mannose synthase consists of three subunits, DPM1, DPM2 and DPM3. *EMBO J* 2000, *19* (11), 2475-82.
56. Timchenko, N. A.; Cai, Z. J.; Welm, A. L.; Reddy, S.; Ashizawa, T.; Timchenko, L. T., RNA CUG repeats sequester CUGBP1 and alter protein levels and activity of CUGBP1. *J Biol Chem* 2001, *276* (11), 7820-6.
57. Rochman, M.; Malicet, C.; Bustin, M., HMGN5/NSBP1: a new member of the HMGN protein family that affects chromatin structure and function. *Biochim Biophys Acta* 2010, *1799* (1-2), 86-92.
58. Jin, S. B.; Zhao, J.; Bjork, P.; Schmekel, K.; Ljungdahl, P. O.; Wieslander, L., Mrd1p is required for processing of pre-rRNA and for maintenance of steady-state levels of 40 S ribosomal subunits in yeast. *J Biol Chem* 2002, *277* (21), 18431-9.
59. Snider, J.; Kittanakom, S.; Damjanovic, D.; Curak, J.; Wong, V.; Stagljar, I., Detecting interactions with membrane proteins using a membrane two-hybrid assay in yeast. *Nat Protoc* 2010, *5* (7), 1281-93.
60. Russo, L. C.; Farias, J. O.; Ferruzo, P. Y. M.; Monteiro, L. F.; Forti, F. L., Revisiting the roles of VHR/DUSP3 phosphatase in human diseases. *Clinics (Sao Paulo)* 2018, *73* (suppl 1), e466s.
61. Wendeler, M. W.; Paccaud, J. P.; Hauri, H. P., Role of Sec24 isoforms in selective export of membrane proteins from the endoplasmic reticulum. *EMBO Rep* 2007, *8* (3), 258-64.
62. Akiyama, H.; Fujisawa, N.; Tashiro, Y.; Takanabe, N.; Sugiyama, A.; Tashiro, F., The role of transcriptional corepressor Nif3l1 in early stage of neural differentiation via cooperation with Trip15/CSN2. *J Biol Chem* 2003, *278* (12), 10752-62.
63. Lu, X.; Ng, H. H.; Bubulya, P. A., The role of SON in splicing, development, and disease. *Wiley Interdiscip Rev RNA* 2014, *5* (5), 637-46.
64. Hu, H.; Wang, Z.; Li, M.; Zeng, F.; Wang, K.; Huang, R.; Wang, H.; Yang, F.; Liang, T.; Huang, H.; Jiang, T., Gene Expression and Methylation Analyses Suggest DCTD as a Prognostic Factor in Malignant Glioma. *Sci Rep* 2017, *7* (1), 11568.
65. Schou, K. B.; Mogensen, J. B.; Morthorst, S. K.; Nielsen, B. S.; Aleliunaite, A.; Serra-Marques, A.; Fürstenberg, N.; Saunier, S.; Bizet, A. A.; Veland, I. R.; Akhmanova, A.; Christensen, S. T.; Pedersen, L. B., KIF13B establishes a CAV1-enriched microdomain at the ciliary transition zone to promote Sonic hedgehog signalling. *Nat Commun* 2017, *8*, 14177.

66. Rachlin, A. S.; Otey, C. A., Identification of palladin isoforms and characterization of an isoform-specific interaction between Lasp-1 and palladin. *J Cell Sci* 2006, *119* (Pt 6), 995-1004.
67. Bend, R.; Cohen, L.; Carter, M. T.; Lyons, M. J.; Niyazov, D.; Mikati, M. A.; Rojas, S. K.; Person, R. E.; Si, Y.; Wentzensen, I. M.; Torti, E.; Lee, J. A.; Boycott, K. M.; Basel-Salmon, L.; Ferreira, C. R.; Gonzaga-Jauregui, C.; Center, R. G., Phenotype and mutation expansion of the PTPN23 associated disorder characterized by neurodevelopmental delay and structural brain abnormalities. *Eur J Hum Genet* 2020, *28* (1), 76-87.
68. Tanabe, K.; Kon, S.; Ichijo, N.; Funaki, T.; Natsume, W.; Watanabe, T.; Satake, M., A SMAP gene family encoding ARF GTPase-activating proteins and its implication in membrane trafficking. *Methods Enzymol* 2008, *438*, 155-70.
69. Brobeil, A.; Bobrich, M.; Tag, C.; Wimmer, M., PTPIP51 in protein interactions: regulation and in situ interacting partners. *Cell Biochem Biophys* 2012, *63* (3), 211-22.
70. Tu, Y.; Popov, S.; Slaughter, C.; Ross, E. M., Palmitoylation of a conserved cysteine in the regulator of G protein signaling (RGS) domain modulates the GTPase-activating activity of RGS4 and RGS10. *J Biol Chem* 1999, *274* (53), 38260-7.
71. Schwarz, R. I., Collagen I and the fibroblast: high protein expression requires a new paradigm of post-transcriptional, feedback regulation. *Biochem Biophys Rep* 2015, *3*, 38-44.
72. Kapoor, N.; Gupta, R.; Menon, S. T.; Folta-Stogniew, E.; Raleigh, D. P.; Sakmar, T. P., Nucleobindin 1 is a calcium-regulated guanine nucleotide dissociation inhibitor of G{alpha}i1. *J Biol Chem* 2010, *285* (41), 31647-60.
73. Kevenaar, J. T.; Bianchi, S.; van Spronsen, M.; Olieric, N.; Lipka, J.; Frias, C. P.; Mikhaylova, M.; Harterink, M.; Keijzer, N.; Wulf, P. S.; Hilbert, M.; Kapitein, L. C.; de Graaff, E.; Ahkmanova, A.; Steinmetz, M. O.; Hoogenraad, C. C., Kinesin-Binding Protein Controls Microtubule Dynamics and Cargo Trafficking by Regulating Kinesin Motor Activity. *Curr Biol* 2016, *26* (7), 849-61.
74. Herlihy, A. E.; Boeing, S.; Weems, J. C.; Walker, J.; Dirac-Svejstrup, A. B.; Lehner, M. H.; Conaway, R. C.; Conaway, J. W.; Svejstrup, J. Q., UBAP2/UBAP2L regulate UV-induced ubiquitylation of RNA polymerase II and are the human orthologues of yeast Def1. *DNA Repair (Amst)* 2022, *115*, 103343.
75. Cavdar Koc, E.; Burkhart, W.; Blackburn, K.; Moseley, A.; Spremulli, L. L., The small subunit of the mammalian mitochondrial ribosome. Identification of the full complement of ribosomal proteins present. *J Biol Chem* 2001, *276* (22), 19363-74.
76. Burns, R.; Majczenko, K.; Xu, J.; Peng, W.; Yapici, Z.; Dowling, J. J.; Li, J. Z.; Burmeister, M., Homozygous splice mutation in CWF19L1 in a Turkish family with recessive ataxia syndrome. *Neurology* 2014, *83* (23), 2175-82.
77. Hurley, J. H.; Emr, S. D., The ESCRT complexes: structure and mechanism of a membrane-trafficking network. *Annu Rev Biophys Biomol Struct* 2006, *35*, 277-98.
78. Rosing, M.; Ossendorf, E.; Rak, A.; Barnekow, A., Giantin interacts with both the small GTPase Rab6 and Rab1. *Exp Cell Res* 2007, *313* (11), 2318-25.
79. Guo, X.; Engel, J. L.; Xiao, J.; Tagliabracci, V. S.; Wang, X.; Huang, L.; Dixon, J. E., UBLCP1 is a 26S proteasome phosphatase that regulates nuclear proteasome activity. *Proc Natl Acad Sci U S A* 2011, *108* (46), 18649-54.
80. Boczonadi, V.; Müller, J. S.; Pyle, A.; Munkley, J.; Dor, T.; Quartararo, J.; Ferrero, I.; Karcagi, V.; Giunta, M.; Polvikoski, T.; Birchall, D.; Princzinger, A.; Cinnamon, Y.; Lützkendorf, S.; Piko, H.; Reza, M.; Florez, L.; Santibanez-Koref, M.; Griffin, H.;

Schuelke, M.; Elpeleg, O.; Kalaydjieva, L.; Lochmüller, H.; Elliott, D. J.; Chinnery, P. F.; Edvardson, S.; Horvath, R., EXOSC8 mutations alter mRNA metabolism and cause hypomyelination with spinal muscular atrophy and cerebellar hypoplasia. *Nat Commun* 2014, *5*, 4287.

81. Sbrissa, D.; Ikonomov, O. C.; Fu, Z.; Ijuin, T.; Gruenberg, J.; Takenawa, T.; Shisheva, A., Core protein machinery for mammalian phosphatidylinositol 3,5-bisphosphate synthesis and turnover that regulates the progression of endosomal transport. Novel Sac phosphatase joins the ArPIKfyve-PIKfyve complex. *J Biol Chem* 2007, *282* (33), 23878-91.
82. Goppelt, A.; Stelzer, G.; Lottspeich, F.; Meisterernst, M., A mechanism for repression of class II gene transcription through specific binding of NC2 to TBP-promoter complexes via heterodimeric histone fold domains. *EMBO J* 1996, *15* (12), 3105-16.
83. Damianov, A.; Kann, M.; Lane, W. S.; Bindereif, A., Human RBM28 protein is a specific nucleolar component of the spliceosomal snRNPs. *Biol Chem* 2006, *387* (10-11), 1455-60.
84. Ye, Y.; Shibata, Y.; Yun, C.; Ron, D.; Rapoport, T. A., A membrane protein complex mediates retro-translocation from the ER lumen into the cytosol. *Nature* 2004, *429* (6994), 841-7.
85. Vukotic, M.; Oeljeklaus, S.; Wiese, S.; Vögtle, F. N.; Meisinger, C.; Meyer, H. E.; Zieseniss, A.; Katschinski, D. M.; Jans, D. C.; Jakobs, S.; Warscheid, B.; Rehling, P.; Deckers, M., Rcf1 mediates cytochrome oxidase assembly and respirasome formation, revealing heterogeneity of the enzyme complex. *Cell Metab* 2012, *15* (3), 336-47.
86. Huang, G.; Shigesada, K.; Ito, K.; Wee, H. J.; Yokomizo, T.; Ito, Y., Dimerization with PEBP2beta protects RUNX1/AML1 from ubiquitin-proteasome-mediated degradation. *EMBO J* 2001, *20* (4), 723-33.
87. Burman, J. L.; Bourbonniere, L.; Philie, J.; Stroh, T.; Dejgaard, S. Y.; Presley, J. F.; McPherson, P. S., Scyl1, mutated in a recessive form of spinocerebellar neurodegeneration, regulates COPI-mediated retrograde traffic. *J Biol Chem* 2008, *283* (33), 22774-86.
88. Sarkar, A. A.; Zohn, I. E., Hectd1 regulates intracellular localization and secretion of Hsp90 to control cellular behavior of the cranial mesenchyme. *J Cell Biol* 2012, *196* (6), 789-800.
89. Adams, G. N.; LaRusch, G. A.; Stavrou, E.; Zhou, Y.; Nieman, M. T.; Jacobs, G. H.; Cui, Y.; Lu, Y.; Jain, M. K.; Mahdi, F.; Shariat-Madar, Z.; Okada, Y.; D'Alecy, L. G.; Schmaier, A. H., Murine prolylcarboxypeptidase depletion induces vascular dysfunction with hypertension and faster arterial thrombosis. *Blood* 2011, *117* (14), 3929-37.
90. Koc, E. C.; Burkhart, W.; Blackburn, K.; Moyer, M. B.; Schlatzer, D. M.; Moseley, A.; Spremulli, L. L., The large subunit of the mammalian mitochondrial ribosome. Analysis of the complement of ribosomal proteins present. *J Biol Chem* 2001, *276* (47), 43958-69.
91. Agrimi, G.; Russo, A.; Scarcia, P.; Palmieri, F., The human gene SLC25A17 encodes a peroxisomal transporter of coenzyme A, FAD and NAD+. *Biochem J* 2012, *443* (1), 241-7.
92. Barik, S.; Banerjee, A. K., Phosphorylation by cellular casein kinase II is essential for transcriptional activity of vesicular stomatitis virus phosphoprotein P. *Proc Natl Acad Sci U S A* 1992, *89* (14), 6570-4.
93. Kalousek, F.; Isaya, G.; Rosenberg, L. E., Rat liver mitochondrial intermediate peptidase (MIP): purification and initial characterization. *EMBO J* 1992, *11* (8), 2803-9.
94. Kondo, H.; Matsumura, T.; Kaneko, M.; Inoue, K.; Kosako, H.; Ikawa, M.; Takahama, Y.; Ohigashi, I., PITHD1 is a proteasome-interacting protein essential for male fertilization. *J Biol Chem* 2020, *295* (6), 1658-1672.

95. Vazquez-Sanchez, S.; Gonzalez-Lozano, M. A.; Walfenzao, A.; Li, K. W.; van Weering, J. R. T., The endosomal protein sorting nexin 4 is a synaptic protein. *Sci Rep* 2020, *10* (1), 18239.
96. Teng, F. Y.; Wang, Y.; Tang, B. L., The syntaxins. *Genome Biol* 2001, *2* (11), REVIEWS3012.
97. Ciccarelli, F. D.; Proukakis, C.; Patel, H.; Cross, H.; Azam, S.; Patton, M. A.; Bork, P.; Crosby, A. H., The identification of a conserved domain in both spartin and spastin, mutated in hereditary spastic paraplegia. *Genomics* 2003, *81* (4), 437-41.
98. Vagin, O.; Tokhtaeva, E.; Garay, P. E.; Souda, P.; Bassilian, S.; Whitelegge, J. P.; Lewis, R.; Sachs, G.; Wheeler, L.; Aoki, R.; Fernandez-Salas, E., Recruitment of septin cytoskeletal proteins by botulinum toxin A protease determines its remarkable stability. *J Cell Sci* 2014, *127* (Pt 15), 3294-308.
99. Keele, G. R.; Prokop, J. W.; He, H.; Holl, K.; Littrell, J.; Deal, A. W.; Kim, Y.; Kyle, P. B.; Attipoe, E.; Johnson, A. C.; Uhl, K. L.; Sirpilla, O. L.; Jahanbakhsh, S.; Robinson, M.; Levy, S.; Valdar, W.; Garrett, M. R.; Solberg Woods, L. C., Sept8/SEPTIN8 involvement in cellular structure and kidney damage is identified by genetic mapping and a novel human tubule hypoxic model. *Sci Rep* 2021, *11* (1), 2071.
100. Fürst, M.; Zhou, Y.; Merfort, J.; Müller, M., Involvement of PpiD in Sec-dependent protein translocation. *Biochim Biophys Acta Mol Cell Res* 2018, *1865* (2), 273-80.
101. Lahiri, S.; Lee, H.; Mesicek, J.; Fuks, Z.; Haimovitz-Friedman, A.; Kolesnick, R. N.; Futerman, A. H., Kinetic characterization of mammalian ceramide synthases: determination of K(m) values towards sphinganine. *FEBS Lett* 2007, *581* (27), 5289-94.
102. McNally, T.; Huang, Q.; Janis, R. S.; Liu, Z.; Olejniczak, E. T.; Reilly, R. M., Structural analysis of UBL5, a novel ubiquitin-like modifier. *Protein Sci* 2003, *12* (7), 1562-6.
103. Ni, C.; Schmitz, D. A.; Lee, J.; Pawłowski, K.; Wu, J.; Buszczak, M., Labeling of heterochronic ribosomes reveals C1ORF109 and SPATA5 control a late step in human ribosome assembly. *Cell Rep* 2022, *38* (13), 110597.
104. Lin, C.; Zhang, J.; Lu, Y.; Li, X.; Zhang, W.; Lin, W.; Zheng, L., NIT1 suppresses tumour proliferation by activating the TGFβ1-Smad2/3 signalling pathway in colorectal cancer. *Cell Death Dis* 2018, *9* (3), 263.
105. Kutzleb, C.; Sanders, G.; Yamamoto, R.; Wang, X.; Lichte, B.; Petrasch-Parwez, E.; Kilimann, M. W., Paralemmin, a prenyl-palmitoyl-anchored phosphoprotein abundant in neurons and implicated in plasma membrane dynamics and cell process formation. *J Cell Biol* 1998, *143* (3), 795-813.
106. Jurica, M. S.; Licklider, L. J.; Gygi, S. R.; Grigorieff, N.; Moore, M. J., Purification and characterization of native spliceosomes suitable for three-dimensional structural analysis. *RNA* 2002, *8* (4), 426-39.
107. Xu, G. F.; O'Connell, P.; Viskochil, D.; Cawthon, R.; Robertson, M.; Culver, M.; Dunn, D.; Stevens, J.; Gesteland, R.; White, R., The neurofibromatosis type 1 gene encodes a protein related to GAP. *Cell* 1990, *62* (3), 599-608.
108. Dong, H.; O'Brien, R. J.; Fung, E. T.; Lanahan, A. A.; Worley, P. F.; Huganir, R. L., GRIP: a synaptic PDZ domain-containing protein that interacts with AMPA receptors. *Nature* 1997, *386* (6622), 279-84.
109. Prendergast, J.; Umanah, G. K.; Yoo, S. W.; Lagerlöf, O.; Motari, M. G.; Cole, R. N.; Huganir, R. L.; Dawson, T. M.; Dawson, V. L.; Schnaar, R. L., Ganglioside regulation of AMPA receptor trafficking. *J Neurosci* 2014, *34* (39), 13246-58.

110. Cheadle, L.; Biederer, T., The novel synaptogenic protein Farp1 links postsynaptic cytoskeletal dynamics and transsynaptic organization. *J Cell Biol* 2012, *199* (6), 985-1001.
111. Lin, A.; Minden, A.; Martinetto, H.; Claret, F. X.; Lange-Carter, C.; Mercurio, F.; Johnson, G. L.; Karin, M., Identification of a dual specificity kinase that activates the Jun kinases and p38-Mpk2. *Science* 1995, *268* (5208), 286-90.
112. Kremer, B. E.; Haystead, T.; Macara, I. G., Mammalian septins regulate microtubule stability through interaction with the microtubule-binding protein MAP4. *Mol Biol Cell* 2005, *16* (10), 4648-59.
113. Meng, G.; Zhao, Y.; Bai, X.; Liu, Y.; Green, T. J.; Luo, M.; Zheng, X., Structure of human stabilin-1 interacting chitinase-like protein (SI-CLP) reveals a saccharide-binding cleft with lower sugar-binding selectivity. *J Biol Chem* 2010, *285* (51), 39898-904.
114. Straight, S. W.; Chen, L.; Karnak, D.; Margolis, B., Interaction with mLin-7 alters the targeting of endocytosed transmembrane proteins in mammalian epithelial cells. *Mol Biol Cell* 2001, *12* (5), 1329-40.
115. Kamberov, E.; Makarova, O.; Roh, M.; Liu, A.; Karnak, D.; Straight, S.; Margolis, B., Molecular cloning and characterization of Pals, proteins associated with mLin-7. *J Biol Chem* 2000, *275* (15), 11425-31.
116. McPherson, P. S., Regulatory role of SH3 domain-mediated protein-protein interactions in synaptic vesicle endocytosis. *Cell Signal* 1999, *11* (4), 229-38.
117. Poulard, C.; Rambaud, J.; Hussein, N.; Corbo, L.; Le Romancer, M., JMJD6 regulates ERα methylation on arginine. *PLoS One* 2014, *9* (2), e87982.
118. Wu, W.; Chen, Y.; Ye, S.; Yang, H.; Yang, J.; Quan, J., Transcription factor forkhead box K1 regulates miR-32 expression and enhances cell proliferation in colorectal cancer. *Oncol Lett* 2021, *21* (5), 407.
119. Sun, N.; Critchley, D. R.; Paulin, D.; Li, Z.; Robson, R. M., Human alpha-synemin interacts directly with vinculin and metavinculin. *Biochem J* 2008, *409* (3), 657-67.
120. Tu, C.; Ortega-Cava, C. F.; Winograd, P.; Stanton, M. J.; Reddi, A. L.; Dodge, I.; Arya, R.; Dimri, M.; Clubb, R. J.; Naramura, M.; Wagner, K. U.; Band, V.; Band, H., Endosomal-sorting complexes required for transport (ESCRT) pathway-dependent endosomal traffic regulates the localization of active Src at focal adhesions. *Proc Natl Acad Sci U S A* 2010, *107* (37), 16107-12.
121. You, K. T.; Park, J.; Kim, V. N., Role of the small subunit processome in the maintenance of pluripotent stem cells. *Genes Dev* 2015, *29* (19), 2004-9.
122. Raemaekers, T.; Ribbeck, K.; Beaudouin, J.; Annaert, W.; Van Camp, M.; Stockmans, I.; Smets, N.; Bouillon, R.; Ellenberg, J.; Carmeliet, G., NuSAP, a novel microtubule-associated protein involved in mitotic spindle organization. *J Cell Biol* 2003, *162* (6), 1017-29.
123. Carsberg, C. J.; Myers, K. A.; Stern, P. L., Metastasis-associated 5T4 antigen disrupts cell-cell contacts and induces cellular motility in epithelial cells. *Int J Cancer* 1996, *68* (1), 84-92.
124. Pastor-Anglada, M.; Pérez-Torras, S., Emerging Roles of Nucleoside Transporters. *Front Pharmacol* 2018, *9*, 606.
125. Ding, J.; Wang, K.; Liu, W.; She, Y.; Sun, Q.; Shi, J.; Sun, H.; Wang, D. C.; Shao, F., Pore-forming activity and structural autoinhibition of the gasdermin family. *Nature* 2016, *535* (7610), 111-6.

126. Falck, J.; Mailand, N.; Syljuåsen, R. G.; Bartek, J.; Lukas, J., The ATM-Chk2-Cdc25A checkpoint pathway guards against radioresistant DNA synthesis. *Nature* 2001, *410* (6830), 842-7.
127. Tang, T.; Zheng, B.; Chen, S. H.; Murphy, A. N.; Kudlicka, K.; Zhou, H.; Farquhar, M. G., hNOA1 interacts with complex I and DAP3 and regulates mitochondrial respiration and apoptosis. *J Biol Chem* 2009, *284* (8), 5414-24.
128. Yan, B. R.; Li, T.; Coyaud, E.; Laurent, E. M. N.; St-Germain, J.; Zhou, Y.; Kim, P. K.; Raught, B.; Brumell, J. H., C5orf51 is a component of the MON1-CCZ1 complex and controls RAB7A localization and stability during mitophagy. *Autophagy* 2022, *18* (4), 829-40.
129. Lin-Moshier, Y.; Sebastian, P. J.; Higgins, L.; Sampson, N. D.; Hewitt, J. E.; Marchant, J. S., Re-evaluation of the role of calcium homeostasis endoplasmic reticulum protein (CHERP) in cellular calcium signaling. *J Biol Chem* 2013, *288* (1), 355-67.
130. Laity, J. H.; Lee, B. M.; Wright, P. E., Zinc finger proteins: new insights into structural and functional diversity. *Curr Opin Struct Biol* 2001, *11* (1), 39-46.
131. Otten, E. G.; Werner, E.; Crespillo-Casado, A.; Boyle, K. B.; Dharamdasani, V.; Pathe, C.; Santhanam, B.; Randow, F., Ubiquitylation of lipopolysaccharide by RNF213 during bacterial infection. *Nature* 2021, *594* (7861), 111-16.
132. Hurtado-Lorenzo, A.; Skinner, M.; El Annan, J.; Futai, M.; Sun-Wada, G. H.; Bourgoin, S.; Casanova, J.; Wildeman, A.; Bechoua, S.; Ausiello, D. A.; Brown, D.; Marshansky, V., V-ATPase interacts with ARNO and Arf6 in early endosomes and regulates the protein degradative pathway. *Nat Cell Biol* 2006, *8* (2), 124-36.
133. Collard, F.; Delpierre, G.; Stroobant, V.; Matthijs, G.; Van Schaftingen, E., A mammalian protein homologous to fructosamine-3-kinase is a ketosamine-3-kinase acting on psicosamines and ribulosamines but not on fructosamines. *Diabetes* 2003, *52* (12), 2888-95.
134. Liang, X.; Zuo, M. Q.; Zhang, Y.; Li, N.; Ma, C.; Dong, M. Q.; Gao, N., Structural snapshots of human pre-60S ribosomal particles before and after nuclear export. *Nat Commun* 2020, *11* (1), 3542.
135. Adulcikas, J.; Norouzi, S.; Bretag, L.; Sohal, S. S.; Myers, S., The zinc transporter SLC39A7 (ZIP7) harbours a highly-conserved histidine-rich N-terminal region that potentially contributes to zinc homeostasis in the endoplasmic reticulum. *Comput Biol Med* 2018, *100*, 196-202.
136. Pühringer, T.; Hohmann, U.; Fin, L.; Pacheco-Fiallos, B.; Schellhaas, U.; Brennecke, J.; Plaschka, C., Structure of the human core transcription-export complex reveals a hub for multivalent interactions. *Elife* 2020, *9*, e61503.
137. Kassel, O.; Schneider, S.; Heilbock, C.; Litfin, M.; Göttlicher, M.; Herrlich, P., A nuclear isoform of the focal adhesion LIM-domain protein Trip6 integrates activating and repressing signals at AP-1- and NF-kappaB-regulated promoters. *Genes Dev* 2004, *18* (20), 2518-28.
138. Yue, Y.; Liu, J.; Cui, X.; Cao, J.; Luo, G.; Zhang, Z.; Cheng, T.; Gao, M.; Shu, X.; Ma, H.; Wang, F.; Wang, X.; Shen, B.; Wang, Y.; Feng, X.; He, C., VIRMA mediates preferential m. *Cell Discov* 2018, *4*, 10.
139. Yoshimoto, R.; Okawa, K.; Yoshida, M.; Ohno, M.; Kataoka, N., Identification of a novel component C2ORF3 in the lariat-intron complex: lack of C2ORF3 interferes with pre-mRNA splicing via intron turnover pathway. *Genes Cells* 2014, *19* (1), 78-87.

140. Sato, O.; Sakai, T.; Choo, Y. Y.; Ikebe, R.; Watanabe, T. M.; Ikebe, M., Mitochondria-associated myosin 19 processively transports mitochondria on actin tracks in living cells. *J Biol Chem* 2022, *298* (5), 101883.
141. Saito, Y.; Nakagawa, T.; Kakihana, A.; Nakamura, Y.; Nabika, T.; Kasai, M.; Takamori, M.; Yamagishi, N.; Kuga, T.; Hatayama, T.; Nakayama, Y., Yeast Two-Hybrid and One-Hybrid Screenings Identify Regulators of hsp70 Gene Expression. *J Cell Biochem* 2016, *117* (9), 2109-17.
142. Kwiatkowski, S.; Seliga, A. K.; Vertommen, D.; Terreri, M.; Ishikawa, T.; Grabowska, I.; Tiebe, M.; Teleman, A. A.; Jagielski, A. K.; Veiga-da-Cunha, M.; Drozak, J., SETD3 protein is the actin-specific histidine *N*-methyltransferase. *Elife* 2018, *7*, e37921.
143. Firestein, R.; Cleary, M. L., Pseudo-phosphatase Sbf1 contains an N-terminal GEF homology domain that modulates its growth regulatory properties. *J Cell Sci* 2001, *114* (Pt 16), 2921-7.
144. Caron, C.; Pivot-Pajot, C.; van Grunsven, L. A.; Col, E.; Lestrat, C.; Rousseaux, S.; Khochbin, S., Cdyl: a new transcriptional co-repressor. *EMBO Rep* 2003, *4* (9), 877-82.
145. Wu, K.; He, J.; Pu, W.; Peng, Y., The Role of Exportin-5 in MicroRNA Biogenesis and Cancer. *Genomics Proteomics Bioinformatics* 2018, *16* (2), 120-26.
146. Gallo, L. I.; Liao, Y.; Ruiz, W. G.; Clayton, D. R.; Li, M.; Liu, Y. J.; Jiang, Y.; Fukuda, M.; Apodaca, G.; Yin, X. M., TBC1D9B functions as a GTPase-activating protein for Rab11a in polarized MDCK cells. *Mol Biol Cell* 2014, *25* (23), 3779-97.
147. Bajaj, L.; Sharma, J.; di Ronza, A.; Zhang, P.; Eblimit, A.; Pal, R.; Roman, D.; Collette, J. R.; Booth, C.; Chang, K. T.; Sifers, R. N.; Jung, S. Y.; Weimer, J. M.; Chen, R.; Schekman, R. W.; Sardiello, M., A CLN6-CLN8 complex recruits lysosomal enzymes at the ER for Golgi transfer. *J Clin Invest* 2020, *130* (8), 4118-32.
148. Brdicka, T.; Pavlistová, D.; Leo, A.; Bruyns, E.; Korínek, V.; Angelisová, P.; Scherer, J.; Shevchenko, A.; Hilgert, I.; Cerný, J.; Drbal, K.; Kuramitsu, Y.; Kornacker, B.; Horejsí, V.; Schraven, B., Phosphoprotein associated with glycosphingolipid-enriched microdomains (PAG), a novel ubiquitously expressed transmembrane adaptor protein, binds the protein tyrosine kinase csk and is involved in regulation of T cell activation. *J Exp Med* 2000, *191* (9), 1591-604.
149. Ohtake, H.; Ichikawa, N.; Okada, M.; Yamashita, T., Cutting Edge: Transmembrane phosphoprotein Csk-binding protein/phosphoprotein associated with glycosphingolipid-enriched microdomains as a negative feedback regulator of mast cell signaling through the FcepsilonRI. *J Immunol* 2002, *168* (5), 2087-90.
150. Smida, M.; Posevitz-Fejfar, A.; Horejsi, V.; Schraven, B.; Lindquist, J. A., A novel negative regulatory function of the phosphoprotein associated with glycosphingolipid-enriched microdomains: blocking Ras activation. *Blood* 2007, *110* (2), 596-615.
151. Svec, A., Phosphoprotein associated with glycosphingolipid-enriched microdomains/Csk-binding protein: a protein that matters. *Pathol Res Pract* 2008, *204* (11), 785-92.
152. Yu, W.; Wang, Y.; Gong, M.; Pei, F.; Zheng, J., Phosphoprotein associated with glycosphingolipid microdomains 1 inhibits the proliferation and invasion of human prostate cancer cells in vitro through suppression of Ras activation. *Oncol Rep* 2012, *28* (2), 606-14.
153. Lizarbe, M. A.; Barrasa, J. I.; Olmo, N.; Gavilanes, F.; Turnay, J., Annexin-phospholipid interactions. Functional implications. *Int J Mol Sci* 2013, *14* (2), 2652-83.
154. Burns, A. L.; Magendzo, K.; Shirvan, A.; Srivastava, M.; Rojas, E.; Alijani, M. R.; Pollard, H. B., Calcium channel activity of purified human synexin and structure of the human synexin gene. *Proc Natl Acad Sci U S A* 1989, *86* (10), 3798-802.

155. Sheriff, A.; Gaipl, U. S.; Franz, S.; Heyder, P.; Voll, R. E.; Kalden, J. R.; Herrmann, M., Loss of GM1 surface expression precedes annexin V-phycoerythrin binding of neutrophils undergoing spontaneous apoptosis during in vitro aging. *Cytometry A* 2004, *62* (2), 75-80.
156. Benz, J.; Hofmann, A., Annexins: from structure to function. *Biol Chem* 1997, *378* (3-4), 177-83.
157. Sharom, F. J., Complex Interplay between the P-Glycoprotein Multidrug Efflux Pump and the Membrane: Its Role in Modulating Protein Function. *Front Oncol* 2014, *4*, 41.
158. Neumann, J.; Rose-Sperling, D.; Hellmich, U. A., Diverse relations between ABC transporters and lipids: An overview. *Biochim Biophys Acta Biomembr* 2017, *1859* (4), 605-18.
159. Tarling, E. J.; de Aguiar Vallim, T. Q.; Edwards, P. A., Role of ABC transporters in lipid transport and human disease. *Trends Endocrinol Metab* 2013, *24* (7), 342-50.
160. Quazi, F.; Molday, R. S., Differential phospholipid substrates and directional transport by ATP-binding cassette proteins ABCA1, ABCA7, and ABCA4 and disease-causing mutants. *J Biol Chem* 2013, *288* (48), 34414-26.
161. Wu, A.; Wojtowicz, K.; Savary, S.; Hamon, Y.; Trombik, T., Do ABC transporters regulate plasma membrane organization? *Cell Mol Biol Lett* 2020, *25*, 37.
162. Bigay, J.; Antonny, B., Curvature, lipid packing, and electrostatics of membrane organelles: defining cellular territories in determining specificity. *Dev Cell* 2012, *23* (5), 886-95.
163. Zerial, M.; McBride, H., Rab proteins as membrane organizers. *Nat Rev Mol Cell Biol* 2001, *2* (2), 107-17.
164. Choudhury, A.; Dominguez, M.; Puri, V.; Sharma, D. K.; Narita, K.; Wheatley, C. L.; Marks, D. L.; Pagano, R. E., Rab proteins mediate Golgi transport of caveola-internalized glycosphingolipids and correct lipid trafficking in Niemann-Pick C cells. *J Clin Invest* 2002, *109* (12), 1541-50.
165. Pagano, R. E.; Martin, O. C.; Kang, H. C.; Haugland, R. P., A novel fluorescent ceramide analogue for studying membrane traffic in animal cells: accumulation at the Golgi apparatus results in altered spectral properties of the sphingolipid precursor. *J Cell Biol* 1991, *113* (6), 1267-79.
166. Russo, D.; Parashuraman, S.; D'Angelo, G., Glycosphingolipid-Protein Interaction in Signal Transduction. *Int J Mol Sci* 2016, *17* (10), 1732.
167. Bremer, E. G.; Hakomori, S.; Bowen-Pope, D. F.; Raines, E.; Ross, R., Ganglioside-mediated modulation of cell growth, growth factor binding, and receptor phosphorylation. *J Biol Chem* 1984, *259* (11), 6818-25.
168. Tagami, S.; Inokuchi Ji, J.; Kabayama, K.; Yoshimura, H.; Kitamura, F.; Uemura, S.; Ogawa, C.; Ishii, A.; Saito, M.; Ohtsuka, Y.; Sakaue, S.; Igarashi, Y., Ganglioside GM3 participates in the pathological conditions of insulin resistance. *J Biol Chem* 2002, *277* (5), 3085-92.
169. Cevher-Keskin, B., ARF1 and SAR1 GTPases in endomembrane trafficking in plants. *Int J Mol Sci* 2013, *14* (9), 18181-99.
170. Zhang, N.; Zabotina, O. A., Critical Determinants in ER-Golgi Trafficking of Enzymes Involved in Glycosylation. *Plants (Basel)* 2022, *11* (3), 428.
171. Zoldoš, V.; Grgurević, S.; Lauc, G., Epigenetic regulation of protein glycosylation. *Biomol Concepts* 2010, *1* (3-4), 253-61.
172. Masone, M. C.; Morra, V.; Venditti, R., Illuminating the membrane contact sites between the endoplasmic reticulum and the trans-Golgi network. *FEBS Lett* 2019, *593* (22), 3135-48.

173. Wright, K. J.; Baye, L. M.; Olivier-Mason, A.; Mukhopadhyay, S.; Sang, L.; Kwong, M.; Wang, W.; Pretorius, P. R.; Sheffield, V. C.; Sengupta, P.; Slusarski, D. C.; Jackson, P. K., An ARL3-UNC119-RP2 GTPase cycle targets myristoylated NPHP3 to the primary cilium. *Genes Dev* 2011, *25* (22), 2347-60.
174. Shumar, S. A.; Kerr, E. W.; Geldenhuys, W. J.; Montgomery, G. E.; Fagone, P.; Thirawatananond, P.; Saavedra, H.; Gabelli, S. B.; Leonardi, R., Nudt19 is a renal CoA diphosphohydrolase with biochemical and regulatory properties that are distinct from the hepatic Nudt7 isoform. *J Biol Chem* 2018, *293* (11), 4134-48.
175. Hatsuzawa, K.; Sakurai, C., Regulatory Mechanism of SNAP23 in Phagosome Formation and Maturation. *Yonago Acta Med* 2020, *63* (3), 135-45.
176. Zhang, Y.; Zhang, X. F.; Fleming, M. R.; Amiri, A.; El-Hassar, L.; Surguchev, A. A.; Hyland, C.; Jenkins, D. P.; Desai, R.; Brown, M. R.; Gazula, V. R.; Waters, M. F.; Large, C. H.; Horvath, T. L.; Navaratnam, D.; Vaccarino, F. M.; Forscher, P.; Kaczmarek, L. K., Kv3.3 Channels Bind Hax-1 and Arp2/3 to Assemble a Stable Local Actin Network that Regulates Channel Gating. *Cell* 2016, *165* (2), 434-48.
177. Pons-Vizcarra, M.; Kurps, J.; Tawfik, B.; Sørensen, J. B.; van Weering, J. R. T.; Verhage, M., MUNC18-1 regulates the submembrane F-actin network, independently of syntaxin1 targeting, via hydrophobicity in β-sheet 10. *J Cell Sci* 2019, *132* (23), jcs234674.
178. Kaneko, T.; Hamazaki, J.; Iemura, S.; Sasaki, K.; Furuyama, K.; Natsume, T.; Tanaka, K.; Murata, S., Assembly pathway of the Mammalian proteasome base subcomplex is mediated by multiple specific chaperones. *Cell* 2009, *137* (5), 914-25.
179. Wen, Y.; Shatkin, A. J., Transcription elongation factor hSPT5 stimulates mRNA capping. *Genes Dev* 1999, *13* (14), 1774-9.
180. Zhang, Y.; Zhao, M.; Gao, H.; Yu, G.; Zhao, Y.; Yao, F.; Yang, W., MAPK signalling-induced phosphorylation and subcellular translocation of PDHE1α promotes tumour immune evasion. *Nat Metab* 2022, *4* (3), 374-88.
181. Falcon, A.; Doege, H.; Fluitt, A.; Tsang, B.; Watson, N.; Kay, M. A.; Stahl, A., FATP2 is a hepatic fatty acid transporter and peroxisomal very long-chain acyl-CoA synthetase. *Am J Physiol Endocrinol Metab* 2010, *299* (3), E384-93.
182. Conrotto, P.; Corso, S.; Gamberini, S.; Comoglio, P. M.; Giordano, S., Interplay between scatter factor receptors and B plexins controls invasive growth. *Oncogene* 2004, *23* (30), 5131-7.
183. Holzmann, J.; Frank, P.; Löffler, E.; Bennett, K. L.; Gerner, C.; Rossmanith, W., RNase P without RNA: identification and functional reconstitution of the human mitochondrial tRNA processing enzyme. *Cell* 2008, *135* (3), 462-74.
184. Makarova, O. V.; Makarov, E. M.; Lührmann, R., The 65 and 110 kDa SR-related proteins of the U4/U6.U5 tri-snRNP are essential for the assembly of mature spliceosomes. *EMBO J* 2001, *20* (10), 2553-63.
185. Gradi, A.; Imataka, H.; Svitkin, Y. V.; Rom, E.; Raught, B.; Morino, S.; Sonenberg, N., A novel functional human eukaryotic translation initiation factor 4G. *Mol Cell Biol* 1998, *18* (1), 334-42.
186. Bartke, T.; Pohl, C.; Pyrowolakis, G.; Jentsch, S., Dual role of BRUCE as an antiapoptotic IAP and a chimeric E2/E3 ubiquitin ligase. *Mol Cell* 2004, *14* (6), 801-11.
187. Zhang, W.; Wang, L.; Liu, Y.; Xu, J.; Zhu, G.; Cang, H.; Li, X.; Bartlam, M.; Hensley, K.; Li, G.; Rao, Z.; Zhang, X. C., Structure of human lanthionine synthetase C-like protein 1 and its interaction with Eps8 and glutathione. *Genes Dev* 2009, *23* (12), 1387-92.

188. Chen, S.; Blank, M. F.; Iyer, A.; Huang, B.; Wang, L.; Grummt, I.; Voit, R., SIRT7-dependent deacetylation of the U3-55k protein controls pre-rRNA processing. *Nat Commun* 2016, *7*, 10734.
189. Leary, S. C.; Kaufman, B. A.; Pellecchia, G.; Guercin, G. H.; Mattman, A.; Jaksch, M.; Shoubridge, E. A., Human SCO1 and SCO2 have independent, cooperative functions in copper delivery to cytochrome c oxidase. *Hum Mol Genet* 2004, *13* (17), 1839-48.
190. Ruhrberg, C.; Hajibagheri, M. A.; Parry, D. A.; Watt, F. M., Periplakin, a novel component of cornified envelopes and desmosomes that belongs to the plakin family and forms complexes with envoplakin. *J Cell Biol* 1997, *139* (7), 1835-49.
191. Ohkuni, A.; Ohno, Y.; Kihara, A., Identification of acyl-CoA synthetases involved in the mammalian sphingosine 1-phosphate metabolic pathway. *Biochem Biophys Res Commun* 2013, *442* (3-4), 195-201.
192. Maki, T.; Grimaldi, A. D.; Fuchigami, S.; Kaverina, I.; Hayashi, I., CLASP2 Has Two Distinct TOG Domains That Contribute Differently to Microtubule Dynamics. *J Mol Biol* 2015, *427* (14), 2379-95.
193. Sanchez, M. I.; Mercer, T. R.; Davies, S. M.; Shearwood, A. M.; Nygård, K. K.; Richman, T. R.; Mattick, J. S.; Rackham, O.; Filipovska, A., RNA processing in human mitochondria. *Cell Cycle* 2011, *10* (17), 2904-16.
194. Fransen, M.; Terlecky, S. R.; Subramani, S., Identification of a human PTS1 receptor docking protein directly required for peroxisomal protein import. *Proc Natl Acad Sci U S A* 1998, *95* (14), 8087-92.
195. Bracken, A. P.; Pasini, D.; Capra, M.; Prosperini, E.; Colli, E.; Helin, K., EZH2 is downstream of the pRB-E2F pathway, essential for proliferation and amplified in cancer. *EMBO J* 2003, *22* (20), 5323-35.
196. Ito, T.; Yang, M.; May, W. S., RAX, a cellular activator for double-stranded RNA-dependent protein kinase during stress signaling. *J Biol Chem* 1999, *274* (22), 15427-32.
197. Hirano, T.; Kishi, M.; Sugimoto, H.; Taguchi, R.; Obinata, H.; Ohshima, N.; Tatei, K.; Izumi, T., Thioesterase activity and subcellular localization of acylprotein thioesterase 1/lysophospholipase 1. *Biochim Biophys Acta* 2009, *1791* (8), 797-805.
198. Stroud, D. A.; Surgenor, E. E.; Formosa, L. E.; Reljic, B.; Frazier, A. E.; Dibley, M. G.; Osellame, L. D.; Stait, T.; Beilharz, T. H.; Thorburn, D. R.; Salim, A.; Ryan, M. T., Accessory subunits are integral for assembly and function of human mitochondrial complex I. *Nature* 2016, *538* (7623), 123-6.
199. Marteijn, J. A.; van der Meer, L. T.; van Emst, L.; van Reijmersdal, S.; Wissink, W.; de Witte, T.; Jansen, J. H.; Van der Reijden, B. A., Gfi1 ubiquitination and proteasomal degradation is inhibited by the ubiquitin ligase Triad1. *Blood* 2007, *110* (9), 3128-35.
200. Itakura, E.; Kishi-Itakura, C.; Mizushima, N., The hairpin-type tail-anchored SNARE syntaxin 17 targets to autophagosomes for fusion with endosomes/lysosomes. *Cell* 2012, *151* (6), 1256-69.
201. Rauch, J. N.; Zuiderweg, E. R.; Gestwicki, J. E., Non-canonical Interactions between Heat Shock Cognate Protein 70 (Hsc70) and Bcl2-associated Anthanogene (BAG) Co-Chaperones Are Important for Client Release. *J Biol Chem* 2016, *291* (38), 19848-57.
202. Liu, Y. S.; Guo, X. Y.; Hirata, T.; Rong, Y.; Motooka, D.; Kitajima, T.; Murakami, Y.; Gao, X. D.; Nakamura, S.; Kinoshita, T.; Fujita, M., -Glycan-dependent protein folding and endoplasmic reticulum retention regulate GPI-anchor processing. *J Cell Biol* 2018, *217* (2), 585-99.

203. Kalousek, F.; Darigo, M. D.; Rosenberg, L. E., Isolation and characterization of propionyl-CoA carboxylase from normal human liver. Evidence for a protomeric tetramer of nonidentical subunits. *J Biol Chem* 1980, *255* (1), 60-5.
204. Nag, S.; Ma, Q.; Wang, H.; Chumnarnsilpa, S.; Lee, W. L.; Larsson, M.; Kannan, B.; Hernandez-Valladares, M.; Burtnick, L. D.; Robinson, R. C., Ca2+ binding by domain 2 plays a critical role in the activation and stabilization of gelsolin. *Proc Natl Acad Sci U S A* 2009, *106* (33), 13713-8.
205. Hunt, T. W.; Fields, T. A.; Casey, P. J.; Peralta, E. G., RGS10 is a selective activator of G alpha i GTPase activity. *Nature* 1996, *383* (6596), 175-7.
206. Hermans, M. M.; Kroos, M. A.; van Beeumen, J.; Oostra, B. A.; Reuser, A. J., Human lysosomal alpha-glucosidase. Characterization of the catalytic site. *J Biol Chem* 1991, *266* (21), 13507-12.
207. Galjart, N. J.; Morreau, H.; Willemsen, R.; Gillemans, N.; Bonten, E. J.; d'Azzo, A., Human lysosomal protective protein has cathepsin A-like activity distinct from its protective function. *J Biol Chem* 1991, *266* (22), 14754-62.
208. Mariappan, M.; Li, X.; Stefanovic, S.; Sharma, A.; Mateja, A.; Keenan, R. J.; Hegde, R. S., A ribosome-associating factor chaperones tail-anchored membrane proteins. *Nature* 2010, *466* (7310), 1120-4.
209. Oddo, M.; Calandra, T.; Bucala, R.; Meylan, P. R., Macrophage migration inhibitory factor reduces the growth of virulent Mycobacterium tuberculosis in human macrophages. *Infect Immun* 2005, *73* (6), 3783-6.
210. Ramasamy, V.; Ramakrishnan, B.; Boeggeman, E.; Ratner, D. M.; Seeberger, P. H.; Qasba, P. K., Oligosaccharide preferences of beta1,4-galactosyltransferase-I: crystal structures of Met340His mutant of human beta1,4-galactosyltransferase-I with a pentasaccharide and trisaccharides of the N-glycan moiety. *J Mol Biol* 2005, *353* (1), 53-67.
211. Sayed, M.; Pelech, S.; Wong, C.; Marotta, A.; Salh, B., Protein kinase CK2 is involved in G2 arrest and apoptosis following spindle damage in epithelial cells. *Oncogene* 2001, *20* (48), 6994-7005.
212. Kofler, N.; Corti, F.; Rivera-Molina, F.; Deng, Y.; Toomre, D.; Simons, M., The Rab-effector protein RABEP2 regulates endosomal trafficking to mediate vascular endothelial growth factor receptor-2 (VEGFR2)-dependent signaling. *J Biol Chem* 2018, *293* (13), 4805-17.
213. Klier, H. J.; von Figura, K.; Pohlmann, R., Isolation and analysis of the human 46-kDa mannose 6-phosphate receptor gene. *Eur J Biochem* 1991, *197* (1), 23-8.
214. Mohsen, A. W.; Vockley, J., Identification of the active site catalytic residue in human isovaleryl-CoA dehydrogenase. *Biochemistry* 1995, *34* (32), 10146-52.
215. Zhu, G.; Herlyn, M.; Yang, X., TRIM15 and CYLD regulate ERK activation via lysine-63-linked polyubiquitination. *Nat Cell Biol* 2021, *23* (9), 978-91.
216. Cunningham, O.; Gore, M. G.; Mantle, T. J., Initial-rate kinetics of the flavin reductase reaction catalysed by human biliverdin-IXbeta reductase (BVR-B). *Biochem J* 2000, *345 Pt 2* (Pt 2), 393-9.
217. Braun, E.; Hotter, D.; Koepke, L.; Zech, F.; Groß, R.; Sparrer, K. M. J.; Müller, J. A.; Pfaller, C. K.; Heusinger, E.; Wombacher, R.; Sutter, K.; Dittmer, U.; Winkler, M.; Simmons, G.; Jakobsen, M. R.; Conzelmann, K. K.; Pöhlmann, S.; Münch, J.; Fackler, O. T.; Kirchhoff, F.; Sauter, D., Guanylate-Binding Proteins 2 and 5 Exert Broad Antiviral Activity by Inhibiting Furin-Mediated Processing of Viral Envelope Proteins. *Cell Rep* 2019, *27* (7), 2092-2104.e10.

218. Stride, B. D.; Grant, C. E.; Loe, D. W.; Hipfner, D. R.; Cole, S. P.; Deeley, R. G., Pharmacological characterization of the murine and human orthologs of multidrug-resistance protein in transfected human embryonic kidney cells. *Mol Pharmacol* 1997, *52* (3), 344-53.
219. Veltel, S.; Kravchenko, A.; Ismail, S.; Wittinghofer, A., Specificity of Arl2/Arl3 signaling is mediated by a ternary Arl3-effector-GAP complex. *FEBS Lett* 2008, *582* (17), 2501-7.
220. Rishavy, M. A.; Hallgren, K. W.; Yakubenko, A. V.; Shtofman, R. L.; Runge, K. W.; Berkner, K. L., Brønsted analysis reveals Lys218 as the carboxylase active site base that deprotonates vitamin K hydroquinone to initiate vitamin K-dependent protein carboxylation. *Biochemistry* 2006, *45* (44), 13239-48.
221. Nakatsumi, H.; Yonehara, S., Identification of functional regions defining different activity in caspase-3 and caspase-7 within cells. *J Biol Chem* 2010, *285* (33), 25418-25.
222. Raingeaud, J.; Whitmarsh, A. J.; Barrett, T.; Dérijard, B.; Davis, R. J., MKK3- and MKK6-regulated gene expression is mediated by the p38 mitogen-activated protein kinase signal transduction pathway. *Mol Cell Biol* 1996, *16* (3), 1247-55.
223. Dumaz, N.; Milne, D. M.; Meek, D. W., Protein kinase CK1 is a p53-threonine 18 kinase which requires prior phosphorylation of serine 15. *FEBS Lett* 1999, *463* (3), 312-6.
224. Beurel, E.; Grieco, S. F.; Jope, R. S., Glycogen synthase kinase-3 (GSK3): regulation, actions, and diseases. *Pharmacol Ther* 2015, *148*, 114-31.
225. Shiekhattar, R.; Mermelstein, F.; Fisher, R. P.; Drapkin, R.; Dynlacht, B.; Wessling, H. C.; Morgan, D. O.; Reinberg, D., Cdk-activating kinase complex is a component of human transcription factor TFIIH. *Nature* 1995, *374* (6519), 283-7.
226. Lambrecht, J. A.; Flynn, J. M.; Downs, D. M., Conserved YjgF protein family deaminates reactive enamine/imine intermediates of pyridoxal 5'-phosphate (PLP)-dependent enzyme reactions. *J Biol Chem* 2012, *287* (5), 3454-61.
227. Savino, T. M.; Bastos, R.; Jansen, E.; Hernandez-Verdun, D., The nucleolar antigen Nop52, the human homologue of the yeast ribosomal RNA processing RRP1, is recruited at late stages of nucleologenesis. *J Cell Sci* 1999, *112 (Pt 12)*, 1889-900.
228. Kelkar, A.; Dobberstein, B., Sec61beta, a subunit of the Sec61 protein translocation channel at the endoplasmic reticulum, is involved in the transport of Gurken to the plasma membrane. *BMC Cell Biol* 2009, *10*, 11.
229. Mukhopadhyay, S.; Linstedt, A. D., Identification of a gain-of-function mutation in a Golgi P-type ATPase that enhances Mn2+ efflux and protects against toxicity. *Proc Natl Acad Sci U S A* 2011, *108* (2), 858-63.
230. Grossmann, N.; Vakkasoglu, A. S.; Hulpke, S.; Abele, R.; Gaudet, R.; Tampé, R., Mechanistic determinants of the directionality and energetics of active export by a heterodimeric ABC transporter. *Nat Commun* 2014, *5*, 5419.
231. Taieb, D.; Roignot, J.; André, F.; Garcia, S.; Masson, B.; Pierres, A.; Iovanna, J. L.; Soubeyran, P., ArgBP2-dependent signaling regulates pancreatic cell migration, adhesion, and tumorigenicity. *Cancer Res* 2008, *68* (12), 4588-96.
232. Boyle, L.; Rao, L.; Kaur, S.; Fan, X.; Mebane, C.; Hamm, L.; Thornton, A.; Ahrendsen, J. T.; Anderson, M. P.; Christodoulou, J.; Gennerich, A.; Shen, Y.; Chung, W. K., Genotype and defects in microtubule-based motility correlate with clinical severity in. *HGG Adv* 2021, *2* (2), 100026.
